# Supplementary material for: Dynamical and combinatorial coding by MAPK p38 and NFκB in the inflammatory response of macrophages
Source: Mol Syst Biol. 2024 Jun 13;20(8):898–932. doi: 10.1038/s44320-024-00047-4 (PMC11297158; doi:10.1038/s44320-024-00047-4)
Supplement: Supplementary file 1 — Appendix [file 44320_2024_47_MOESM1_ESM.pdf]

<u>Appendix Table of Contents</u>

Appendix Figure S1. MAPK p38 and NFκB have stimulus-specific differential dose responses. Page 2

Appendix Figure S2. MAPK p38 does not improve dose distinction beyond that achieved by NFκB dynamics. Page 3

Appendix Figure S3. MAPK p38 does not improve dose distinction beyond that achieved by NFκB dynamics. Page 4

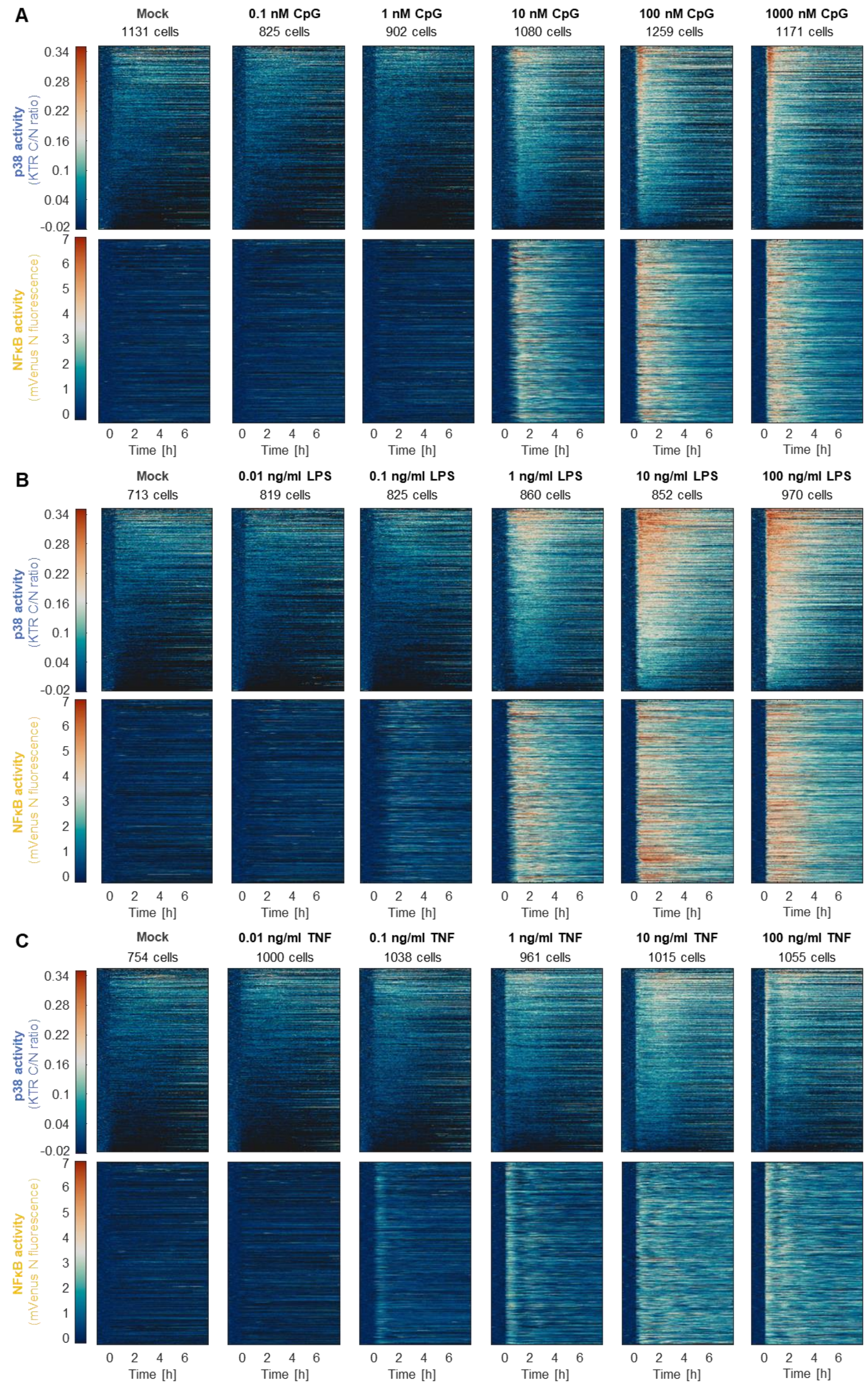

Appendix Figure S1. MAPK p38 and NFκB have stimulus-specific differential dose responses.

(A, B, C) p38 and NFκB activity dynamics in the same cells in response to 0, 0.1, 1, 10, 100, 1000 nM CpG (A), 0, 0.01, 0.1, 1, 10, 100 ng/ml LPS (B) and TNF (C) stimulation over app. 8 h measured by fluorescence microscopy of reporter hMPDMs. Each row of the heatmap represents the p38 or NFκB signaling trajectory of one cell. Trajectories are sorted by maximum amplitude of p38 activity. Data from two pooled biological replicates are depicted. Total # of cells as indicated above heatmaps. Data for high dose stimulations are also shown in Figure 2A, 3A.

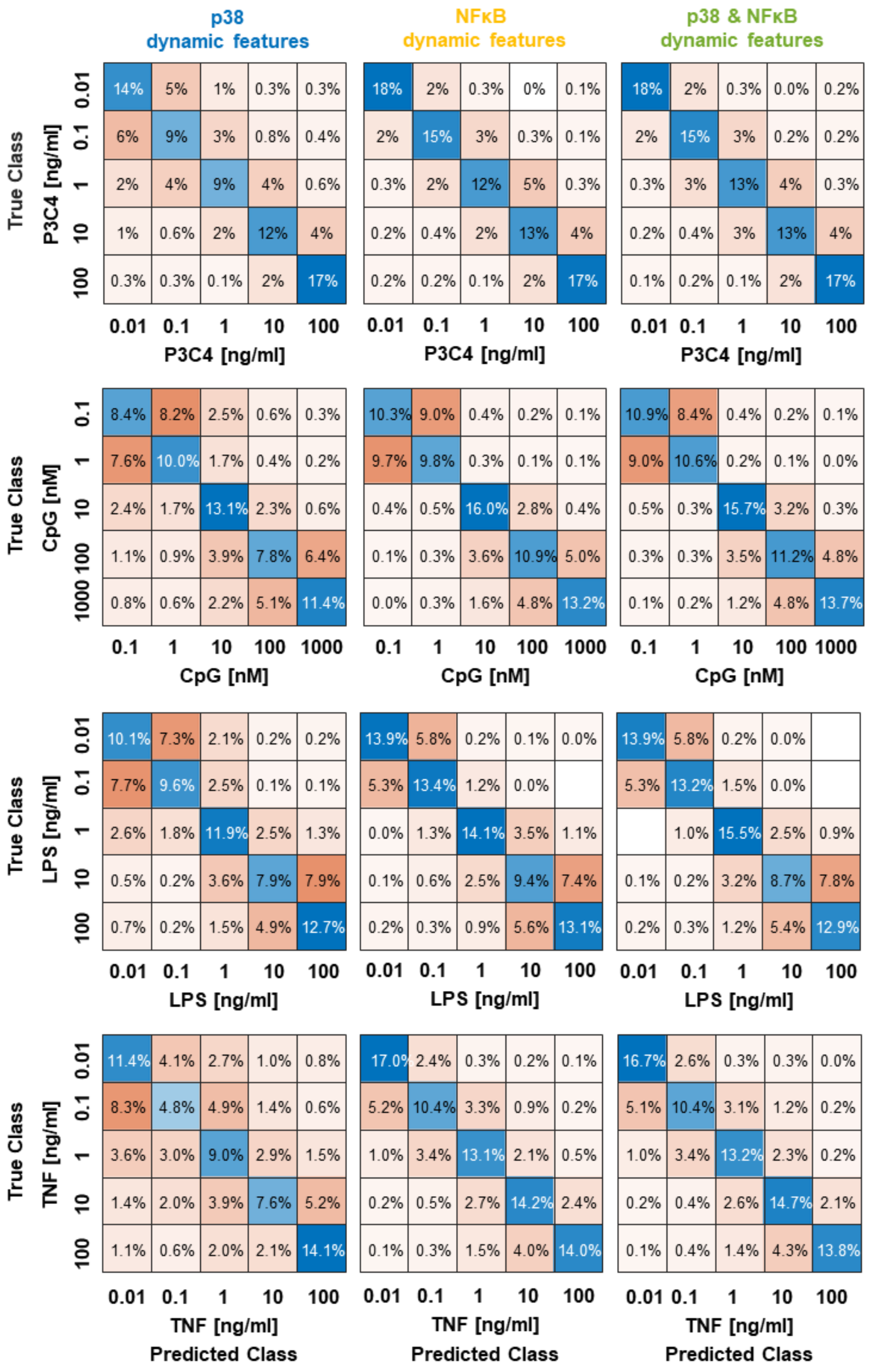

Appendix Figure S2. MAPK p38 does not improve dose distinction beyond that achieved by NFκB dynamics.

Confusion matrices for machine learning classifications of p38 only, NFκB only, or NFκB + p38 dynamic features in response to 5 doses of P3C4 (0.01 – 100 ng/ml), CpG (0.1 – 1000 nM), LPS (0.01 – 100 ng/ml), or TNF (0.01 – 100 ng/ml). Data from two pooled biological replicates are used.

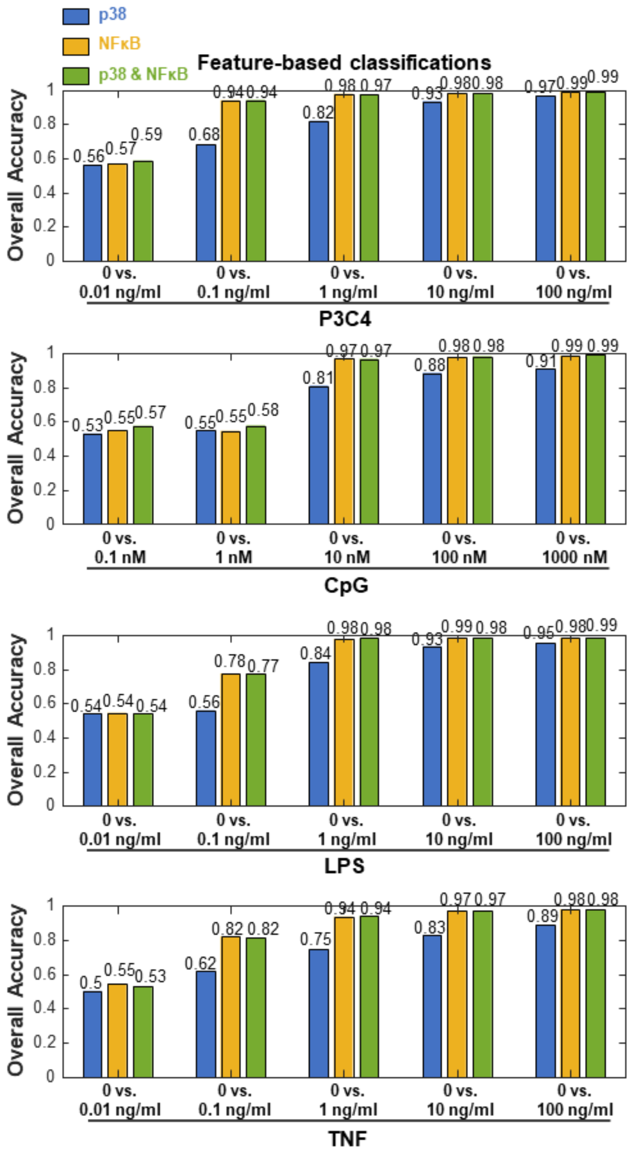

Appendix Figure S3. MAPK p38 does not improve dose distinction beyond that achieved by NFκB dynamics.

Overall classification accuracies of decision tree ensemble classifications using p38 only, NFκB only, or NFκB + p38 dynamic features in response to mock stimulation vs. stimulation with respective indicated dose of the indicated stimuli (P3C4, CpG, LPS, or TNF). Data from two pooled biological replicates are used.
