# Supplementary material for: Dynamical and combinatorial coding by MAPK p38 and NFκB in the inflammatory response of macrophages
Source: Mol Syst Biol. 2024 Jun 13;20(8):898–932. doi: 10.1038/s44320-024-00047-4 (PMC11297158; doi:10.1038/s44320-024-00047-4)
Supplement: Supplementary file 8 — Source data Fig. 1 [file 44320_2024_47_MOESM8_ESM.zip › Source Data for Figure 1/1B/1B WesternBlotsLoadingSchemes.pdf]

## With Figure 1B

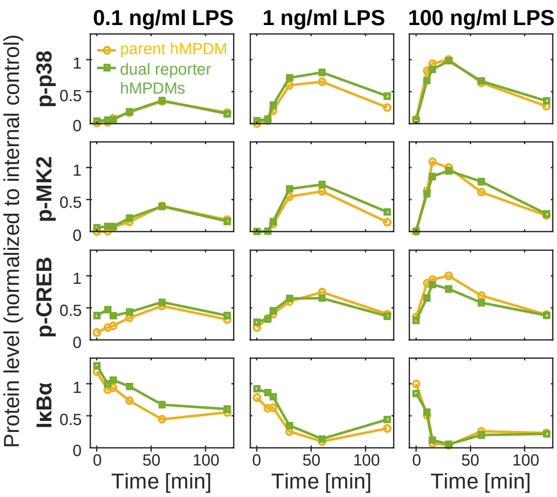


## With Figure EV1C

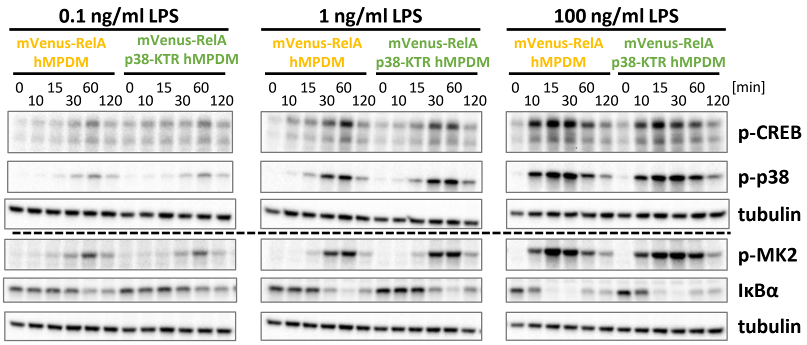


## Loading scheme

Gel 1

LPS stim. [ng/ml]: 0.1

Cell line: mVenus-Rela | mVenus-Rela P38-mCerulean

LPS stim. [min]: 0 10 15 30 60 120 | 0 10 15 30 60 120 | Empty | Int. ctrl: LPS 100 ng/ml 0 min | Int. ctrl: LPS 100 ng/ml 30 min

**p-CREB**

**p-p38**

**Tubulin B**

Gel 2

LPS stim. [ng/ml]: 1 | 100

Cell line: mVenus-Rela | mVenus-Rela P38-mCerulean | mVenus-Rela | mVenus-Rela P38-mCerulean

LPS stim. [min]: 0 10 15 30 60 120 | 0 10 15 30 60 120 | Empty | 0 10 15 30 60 120 | 0 10 15 30 60 120

**p-CREB**

**p-p38**

**Tubulin B**

Gel 1

LPS stim. [ng/ml]: 0.1

Cell line: mVenus-Rela | mVenus-Rela P38-mCerulean

LPS stim. [min]: 0 10 15 30 60 120 | 0 10 15 30 60 120 | Int. ctrl: LPS 100 ng/ml 0 min | Int. ctrl: LPS 100 ng/ml 30 min

**p-MK2**

**IκBα**

**Tubulin A**

Gel 2

LPS stim. [ng/ml]: 1 | 100

Cell line: mVenus-Rela | mVenus-Rela P38-mCerulean | mVenus-Rela | mVenus-Rela P38-mCerulean

LPS stim. [min]: 0 10 15 30 60 120 | 0 10 15 30 60 120 | 0 10 15 30 60 120 | 0 10 15 30 60 120

**p-MK2**

**IκBα**

**Tubulin A**

## Protein size marker

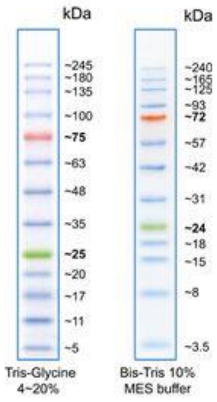


AccuRuler Plus RGB BroadRange Prestained (Biopioneer)
