# Supplementary material for: Dynamical and combinatorial coding by MAPK p38 and NFκB in the inflammatory response of macrophages
Source: Mol Syst Biol. 2024 Jun 13;20(8):898–932. doi: 10.1038/s44320-024-00047-4 (PMC11297158; doi:10.1038/s44320-024-00047-4)
Supplement: Supplementary file 8 — Source data Fig. 1 [file 44320_2024_47_MOESM8_ESM.zip › Source Data for Figure 1/1E/1E Western Blots.pdf]

With Figure 1E

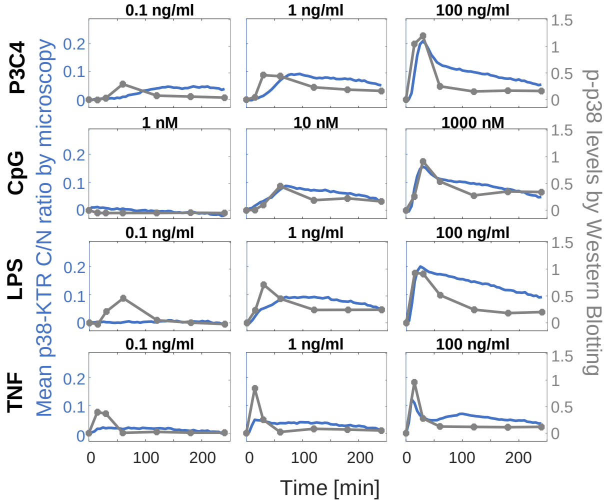

With Figure EV1D

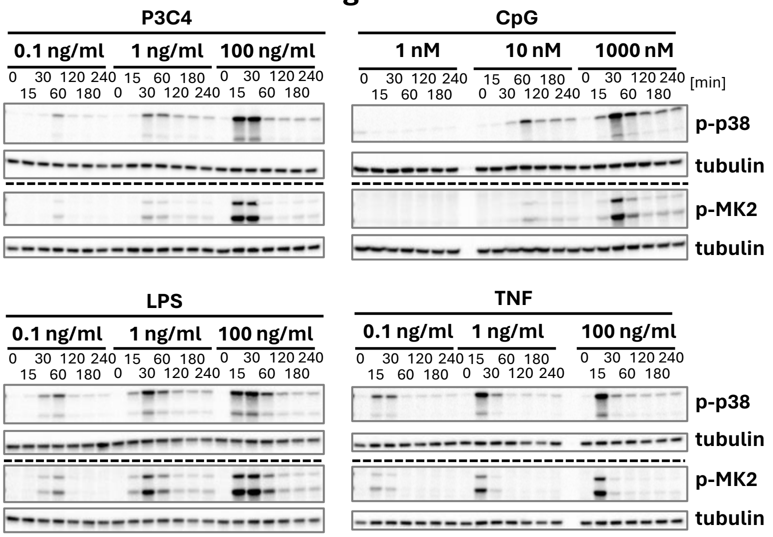

With Figure EV1E

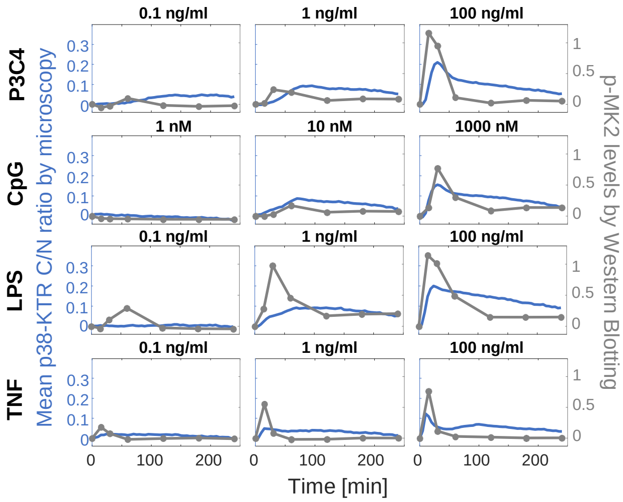

Protein size marker

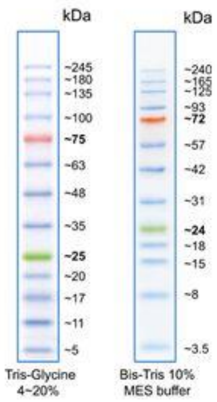

AccuRuler Plus RGB BroadRange Prestained (Biopioneer)

Loading scheme

Stimulation: P3C4
Dose: Int. ctrl LPS H 30 min | P3C4 0.1 ng/ml | P3C4 1 ng/ml | P3C4 100 ng/ml
[min] 0 15 30 60 120 180 240 | 0 15 30 60 120 180 240 | 0 15 30 60 120 180 240

'Gel1': p-p38, tubulin
'Gel2': p-MK2, tubulin

CpG
Int. ctrl LPS H 30 min | CpG 1 nM | empty | CpG 10 nM | CpG 1000 nM
0 15 30 60 120 180 240 | | 0 15 30 60 120 180 240 | 0 15 30 60 120 180 240

'Gel1': p-p38, tubulin
'Gel2': p-MK2, tubulin

Stimulation: LPS
Dose: Int. ctrl LPS H 30 min | LPS 0.1 ng/ml | LPS 1 ng/ml | LPS 100 ng/ml
[min] 0 15 30 60 120 180 240 | 0 15 30 60 120 180 240 | 0 15 30 60 120 180 240

'Gel1': p-p38, tubulin
'Gel2': p-MK2, tubulin

TNF
Int. ctrl LPS H 30 min | TNF 0.1 ng/ml | TNF 1 ng/ml | empty | TNF 100 ng/ml
0 15 30 60 120 180 240 | 0 15 30 60 120 180 240 | | 0 15 30 60 120 180 240

'Gel1': p-p38, tubulin
'Gel2': p-MK2, tubulin
