# Supplementary figures and images for: Dynamical and combinatorial coding by MAPK p38 and NFκB in the inflammatory response of macrophages
Source: Mol Syst Biol. 2024 Jun 13;20(8):898–932. doi: 10.1038/s44320-024-00047-4 (PMC11297158; doi:10.1038/s44320-024-00047-4)

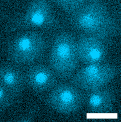

Supplement: Supplementary file 8 — Source data Fig. 1 [file 44320_2024_47_MOESM8_ESM.zip › Source Data for Figure 1/1C/p38i_LPS/LPS p38i_s1t013c3.tif]

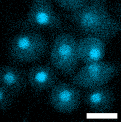

Supplement: Supplementary file 8 — Source data Fig. 1 [file 44320_2024_47_MOESM8_ESM.zip › Source Data for Figure 1/1C/p38i_LPS/LPS p38i_s1t007c3.tif]

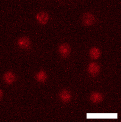

Supplement: Supplementary file 8 — Source data Fig. 1 [file 44320_2024_47_MOESM8_ESM.zip › Source Data for Figure 1/1C/p38i_LPS/LPS p38i_s1t007c1.tif]

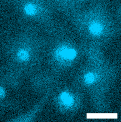

Supplement: Supplementary file 8 — Source data Fig. 1 [file 44320_2024_47_MOESM8_ESM.zip › Source Data for Figure 1/1C/p38i_LPS/LPS p38i_s1t079c3.tif]

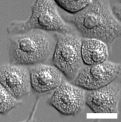

Supplement: Supplementary file 8 — Source data Fig. 1 [file 44320_2024_47_MOESM8_ESM.zip › Source Data for Figure 1/1C/p38i_LPS/LPS p38i_s1t007c4.tif]

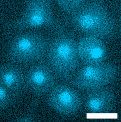

Supplement: Supplementary file 8 — Source data Fig. 1 [file 44320_2024_47_MOESM8_ESM.zip › Source Data for Figure 1/1C/p38i_LPS/LPS p38i_s1t019c3.tif]

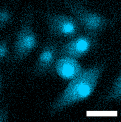

Supplement: Supplementary file 8 — Source data Fig. 1 [file 44320_2024_47_MOESM8_ESM.zip › Source Data for Figure 1/1C/DMSO_Mock/DMSO_t013c3.tif]

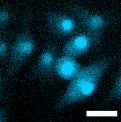

Supplement: Supplementary file 8 — Source data Fig. 1 [file 44320_2024_47_MOESM8_ESM.zip › Source Data for Figure 1/1C/DMSO_Mock/DMSO_t019c3.tif]

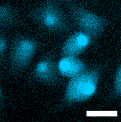

Supplement: Supplementary file 8 — Source data Fig. 1 [file 44320_2024_47_MOESM8_ESM.zip › Source Data for Figure 1/1C/DMSO_Mock/DMSO_t079c3.tif]

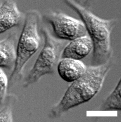

Supplement: Supplementary file 8 — Source data Fig. 1 [file 44320_2024_47_MOESM8_ESM.zip › Source Data for Figure 1/1C/DMSO_Mock/DMSO_t007c4.tif]

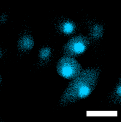

Supplement: Supplementary file 8 — Source data Fig. 1 [file 44320_2024_47_MOESM8_ESM.zip › Source Data for Figure 1/1C/DMSO_Mock/DMSO_t007c3.tif]

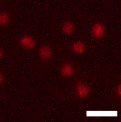

Supplement: Supplementary file 8 — Source data Fig. 1 [file 44320_2024_47_MOESM8_ESM.zip › Source Data for Figure 1/1C/DMSO_Mock/DMSO_t007c1.tif]

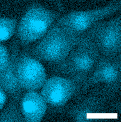

Supplement: Supplementary file 8 — Source data Fig. 1 [file 44320_2024_47_MOESM8_ESM.zip › Source Data for Figure 1/1C/DMSO_LPS/LPS_t019c3.tif]

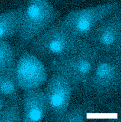

Supplement: Supplementary file 8 — Source data Fig. 1 [file 44320_2024_47_MOESM8_ESM.zip › Source Data for Figure 1/1C/DMSO_LPS/LPS_t079c3.tif]

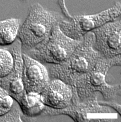

Supplement: Supplementary file 8 — Source data Fig. 1 [file 44320_2024_47_MOESM8_ESM.zip › Source Data for Figure 1/1C/DMSO_LPS/LPS_t007c4.tif]

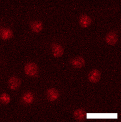

Supplement: Supplementary file 8 — Source data Fig. 1 [file 44320_2024_47_MOESM8_ESM.zip › Source Data for Figure 1/1C/DMSO_LPS/LPS_t007c1.tif]

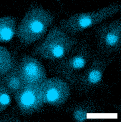

Supplement: Supplementary file 8 — Source data Fig. 1 [file 44320_2024_47_MOESM8_ESM.zip › Source Data for Figure 1/1C/DMSO_LPS/LPS_t007c3.tif]

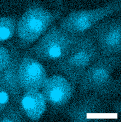

Supplement: Supplementary file 8 — Source data Fig. 1 [file 44320_2024_47_MOESM8_ESM.zip › Source Data for Figure 1/1C/DMSO_LPS/LPS_t013c3.tif]

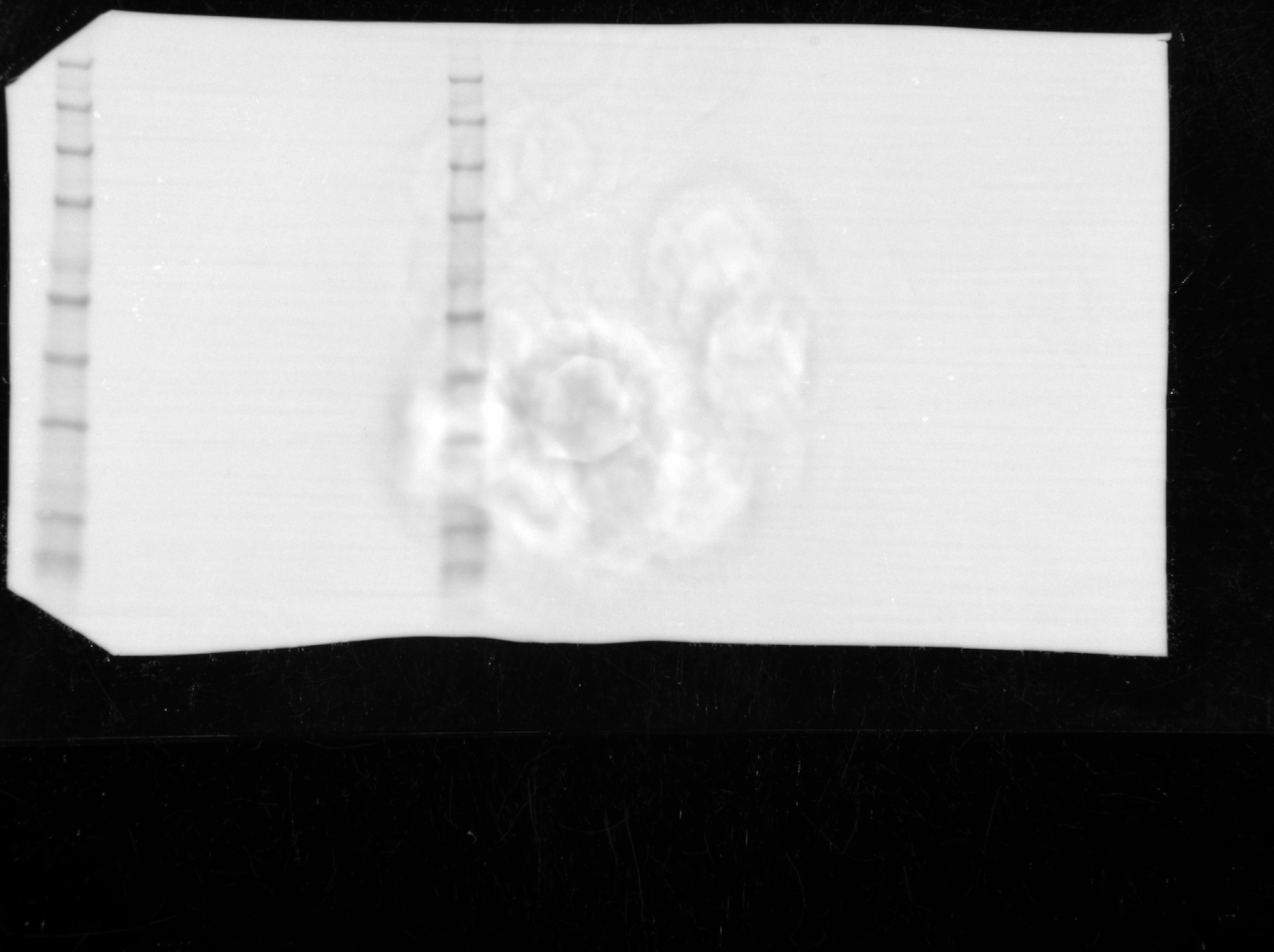

Supplement: Supplementary file 8 — Source data Fig. 1 [file 44320_2024_47_MOESM8_ESM.zip › Source Data for Figure 1/1E/CpG/tubulin gel 2/Image 2021-05-24 13hr 07min coilormietric.tif]

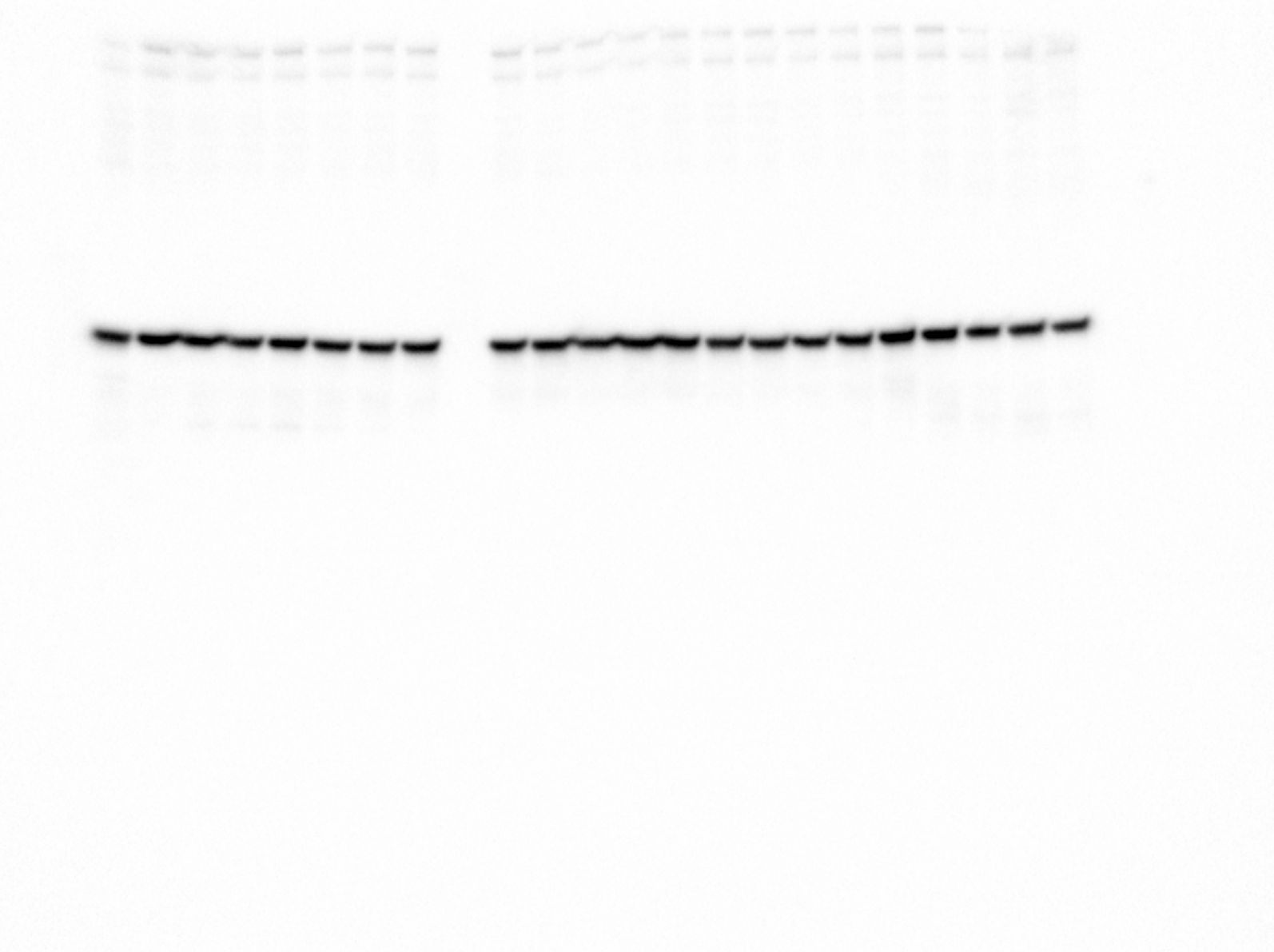

Supplement: Supplementary file 8 — Source data Fig. 1 [file 44320_2024_47_MOESM8_ESM.zip › Source Data for Figure 1/1E/CpG/tubulin gel 2/Image 2021-05-24 13hr 08min_Exposure_4.0sec.tif]

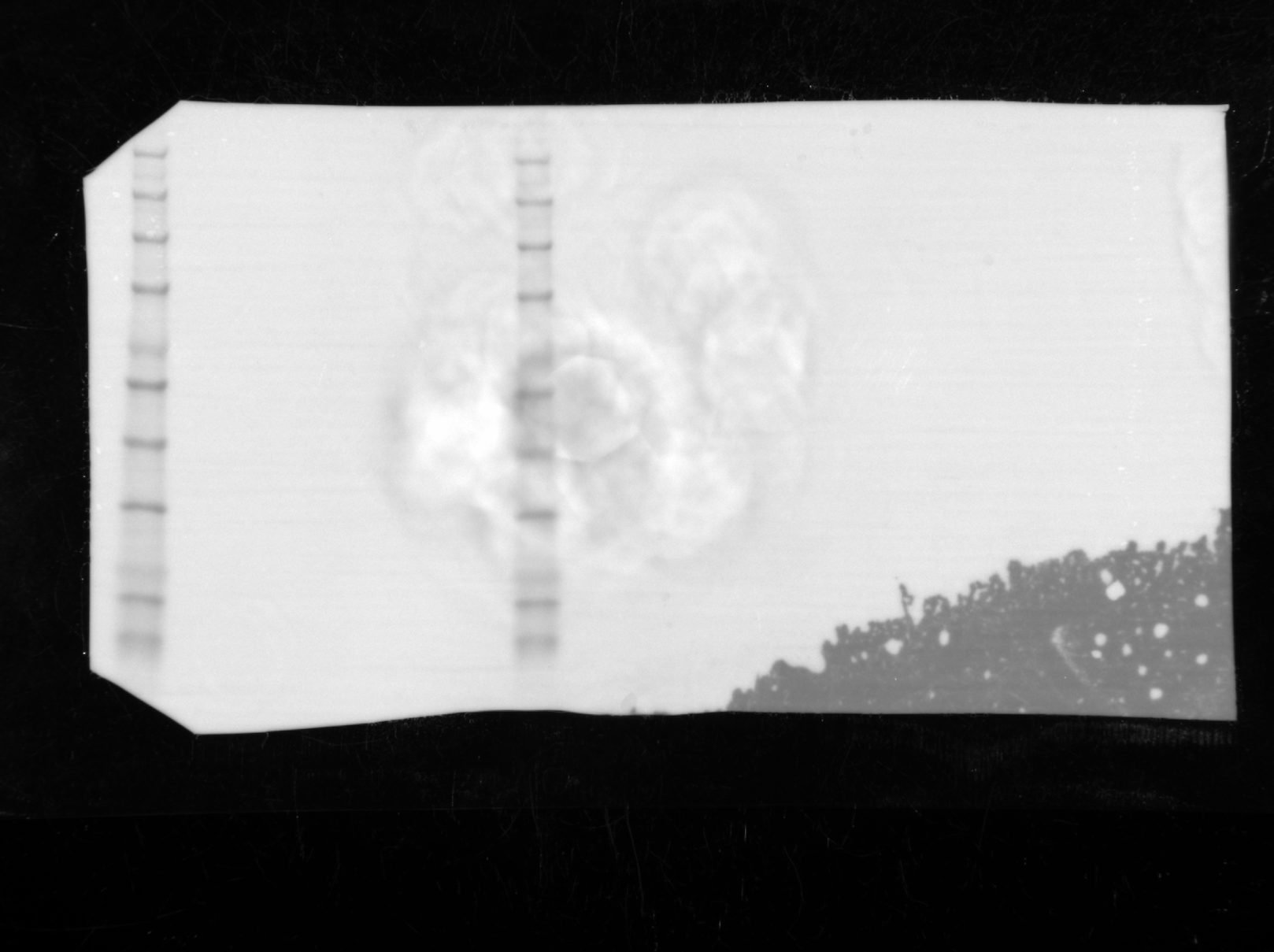

Supplement: Supplementary file 8 — Source data Fig. 1 [file 44320_2024_47_MOESM8_ESM.zip › Source Data for Figure 1/1E/CpG/pMK2/Image 2021-05-18 12hr 32min colorimetric.tif]

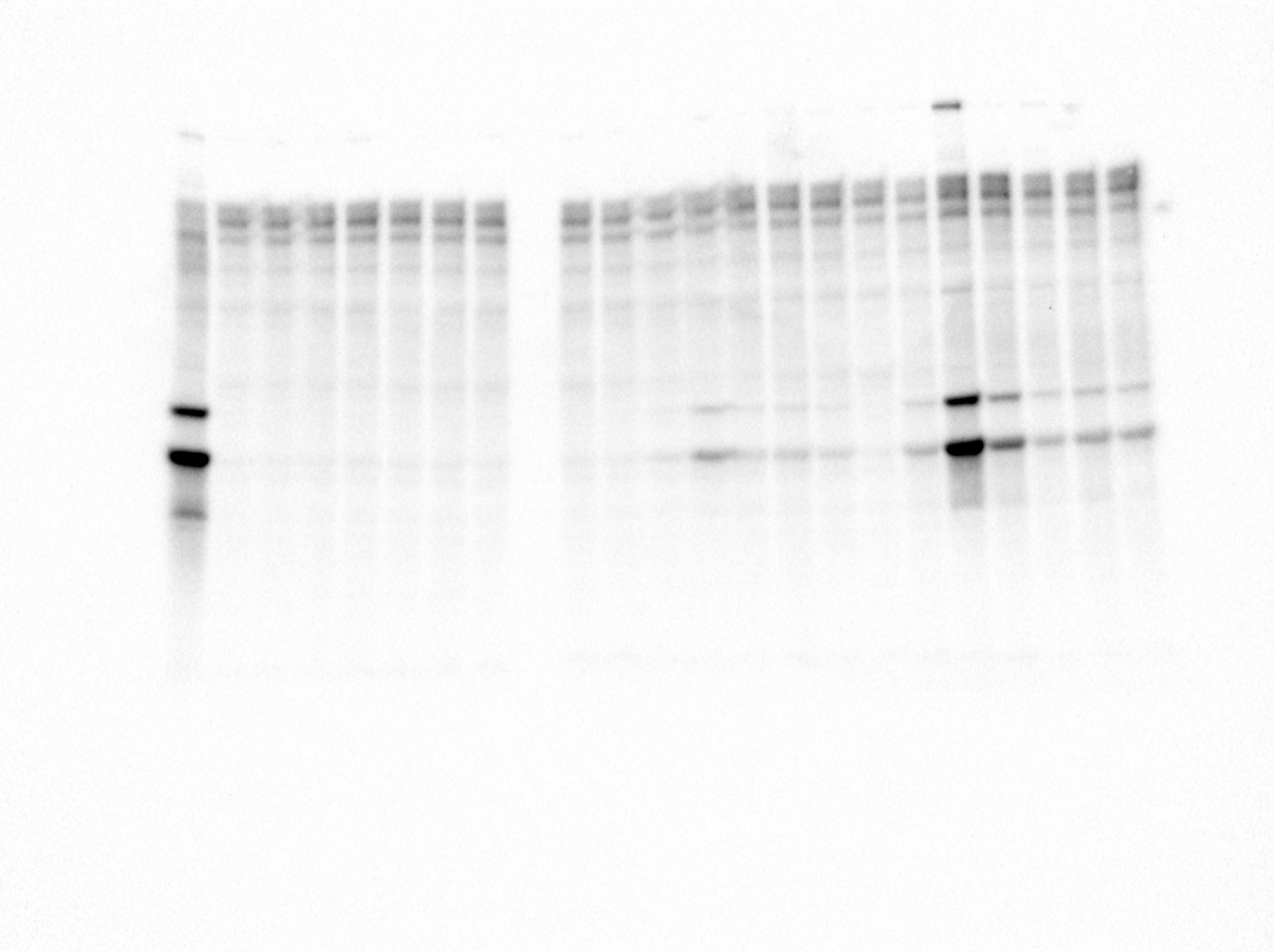

Supplement: Supplementary file 8 — Source data Fig. 1 [file 44320_2024_47_MOESM8_ESM.zip › Source Data for Figure 1/1E/CpG/pMK2/Image 2021-05-18 12hr 34min_Exposure_30.0sec.tif]

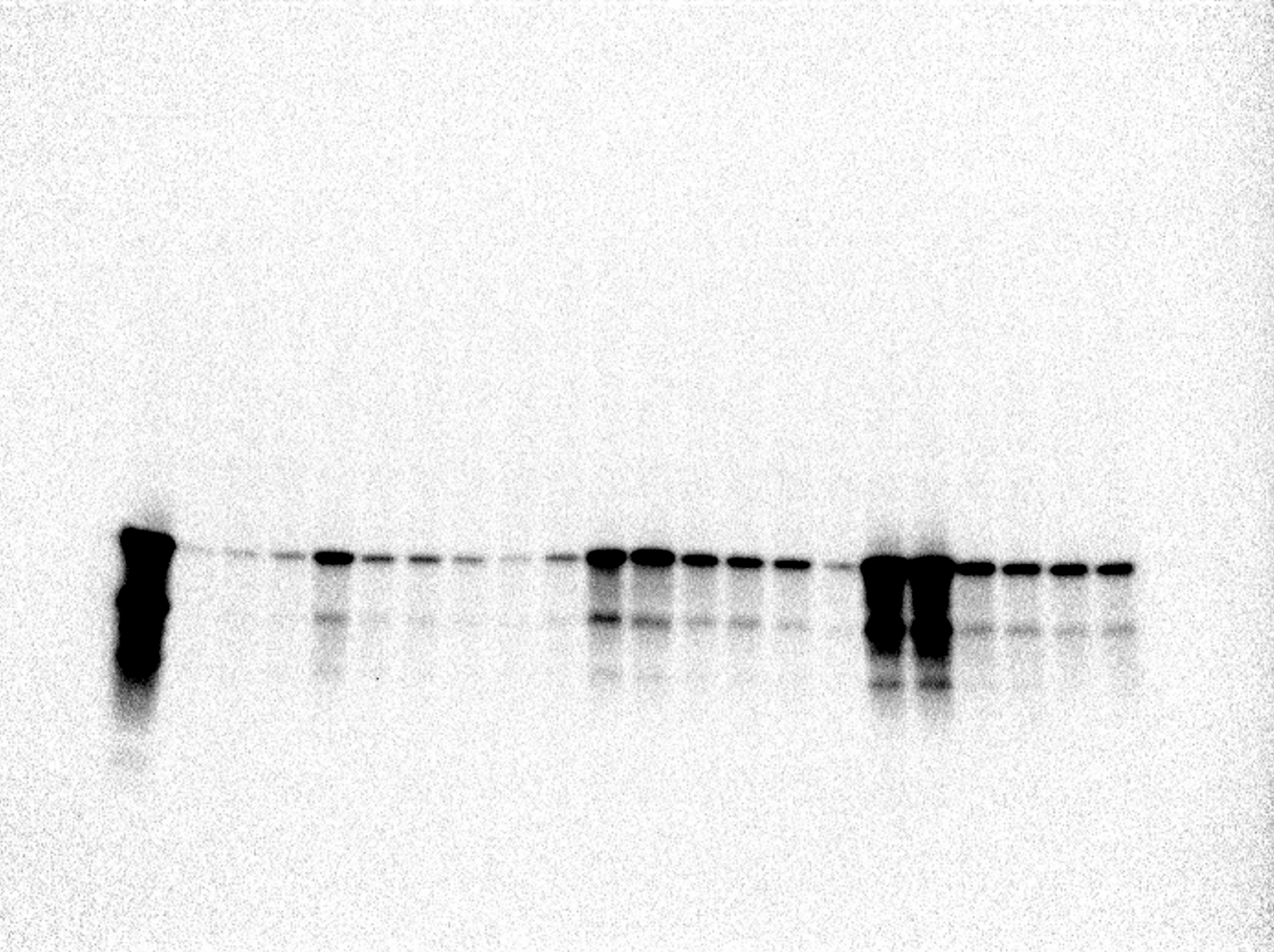

Supplement: Supplementary file 8 — Source data Fig. 1 [file 44320_2024_47_MOESM8_ESM.zip › Source Data for Figure 1/1E/CpG/pp38/Image 2021-05-19 11hr 51min_Exposure_20.0sec with some overexposure.tif]

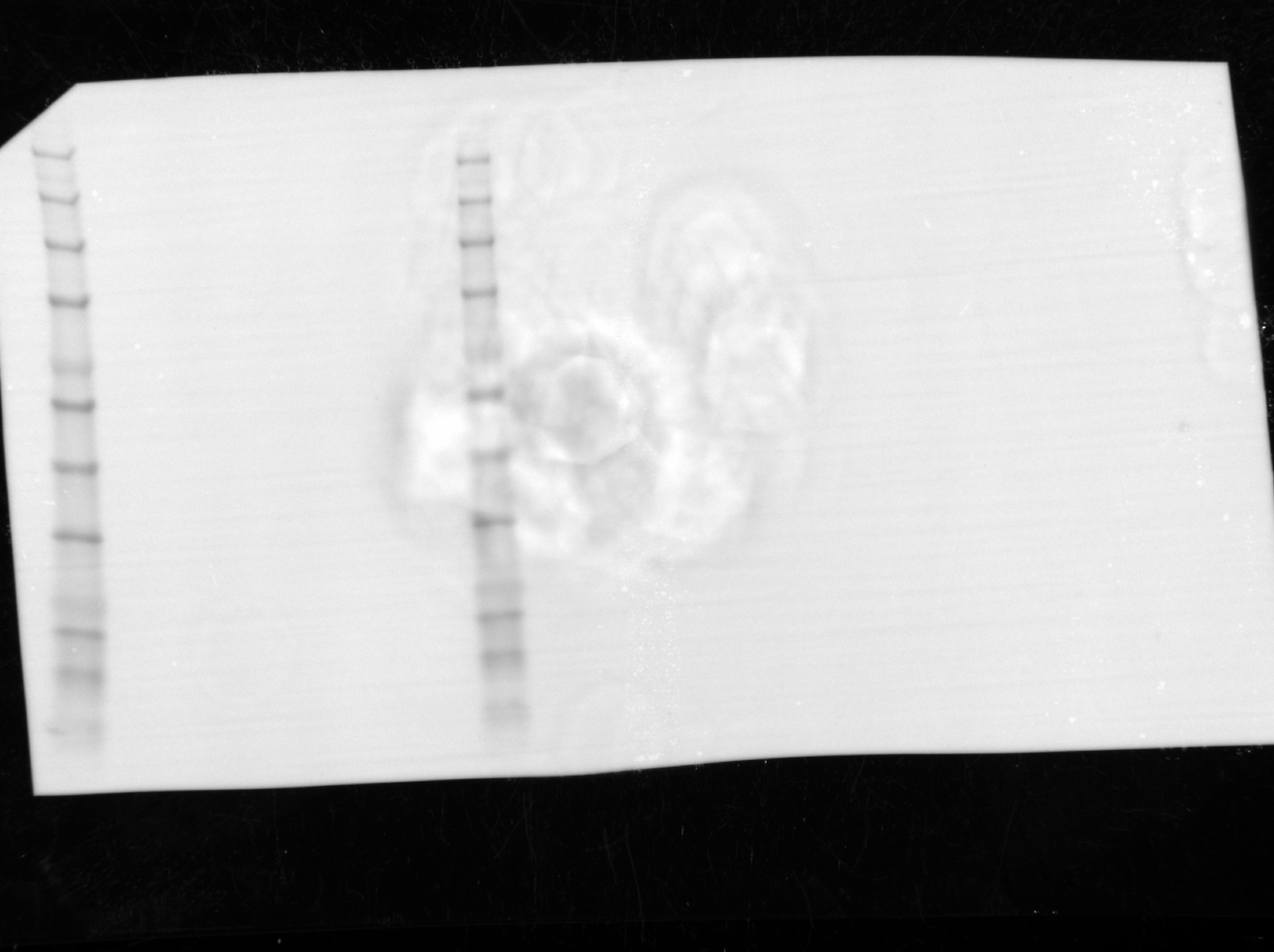

Supplement: Supplementary file 8 — Source data Fig. 1 [file 44320_2024_47_MOESM8_ESM.zip › Source Data for Figure 1/1E/CpG/pp38/Image 2021-05-19 11hr 59min colorimetric.tif]

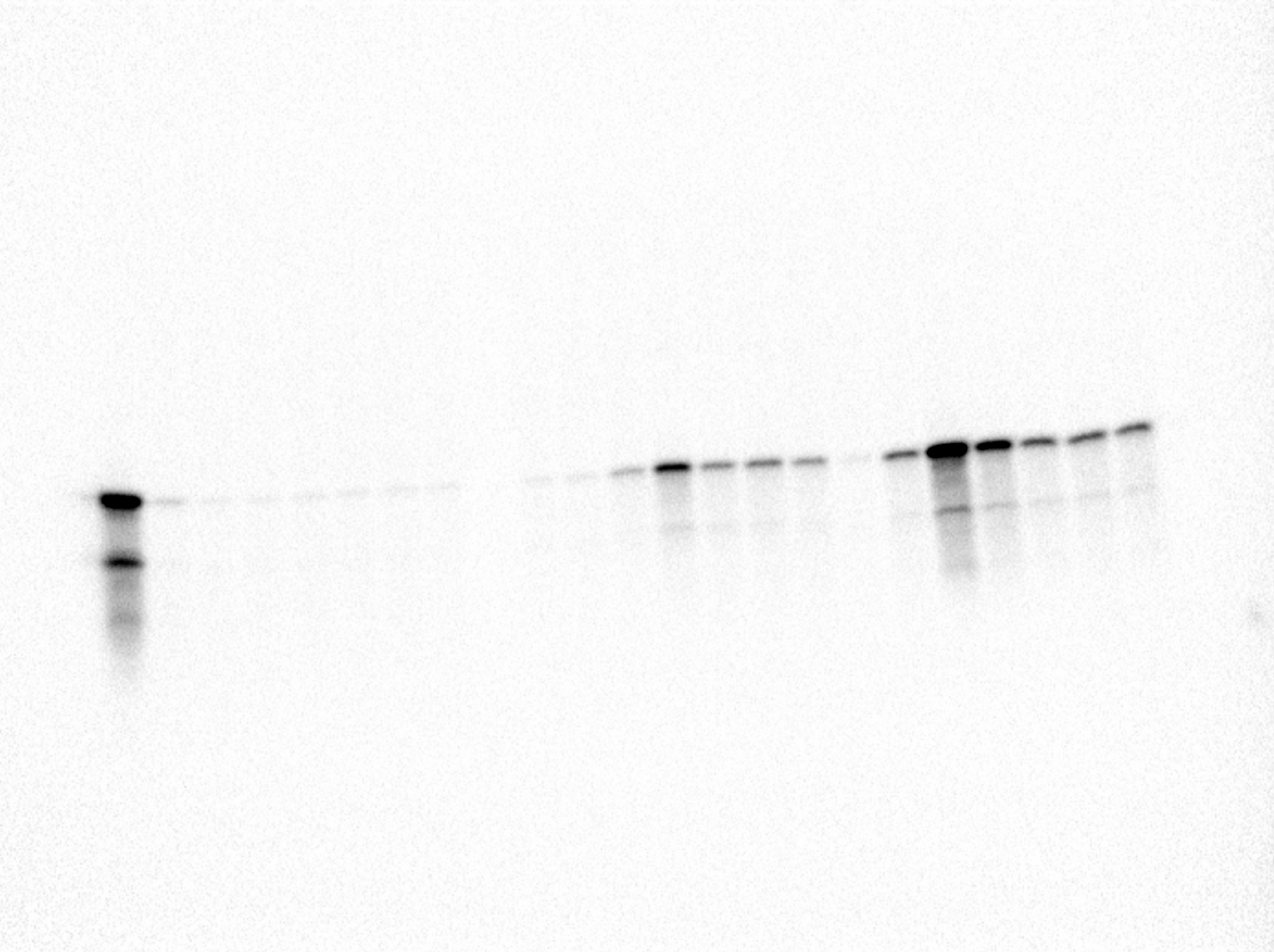

Supplement: Supplementary file 8 — Source data Fig. 1 [file 44320_2024_47_MOESM8_ESM.zip › Source Data for Figure 1/1E/CpG/pp38/Image 2021-05-19 12hr 02min_Exposure_14.0sec.tif]

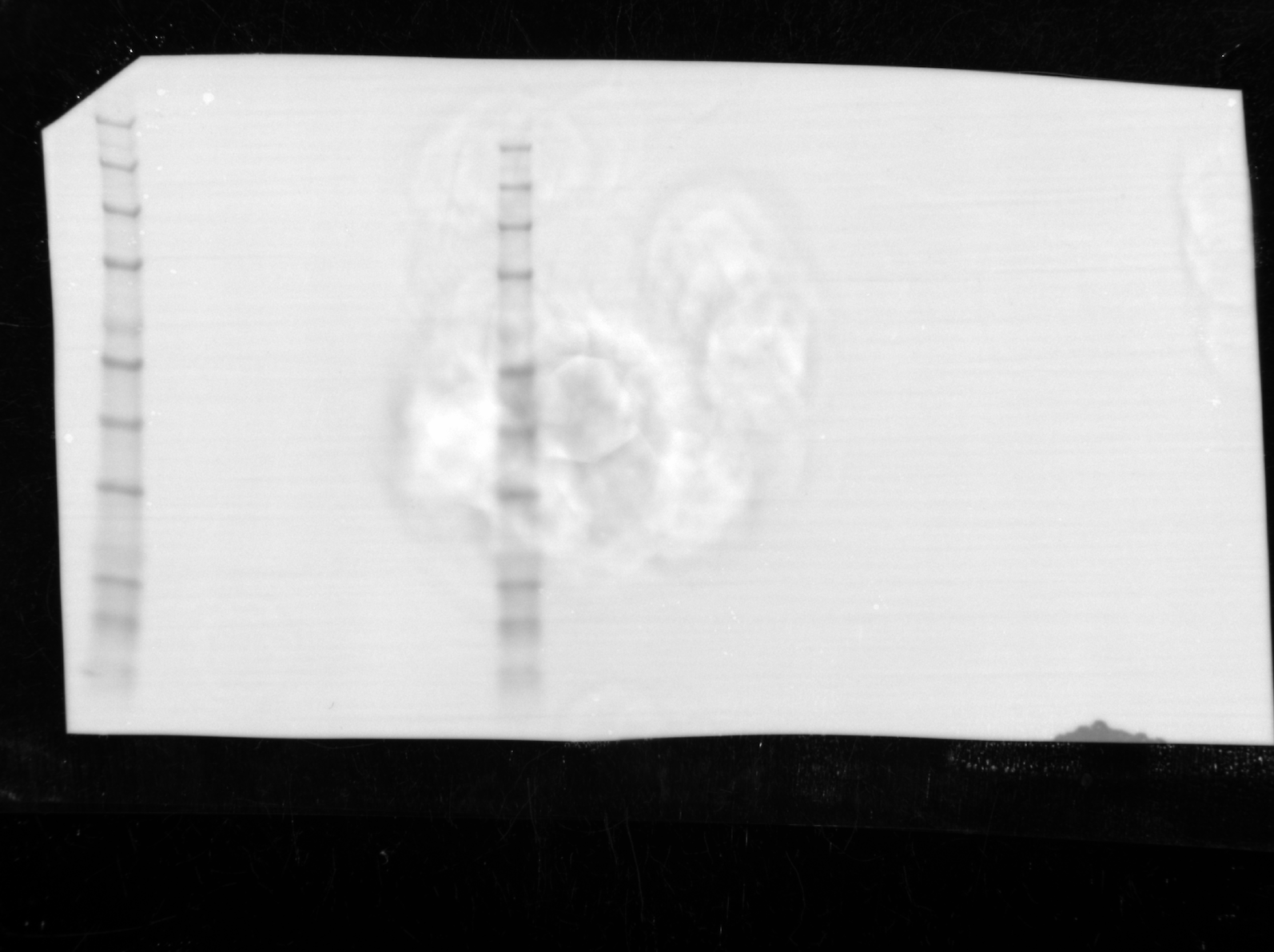

Supplement: Supplementary file 8 — Source data Fig. 1 [file 44320_2024_47_MOESM8_ESM.zip › Source Data for Figure 1/1E/CpG/tubulin gel 1/Image 2021-05-24 12hr 48min colorimetric.tif]

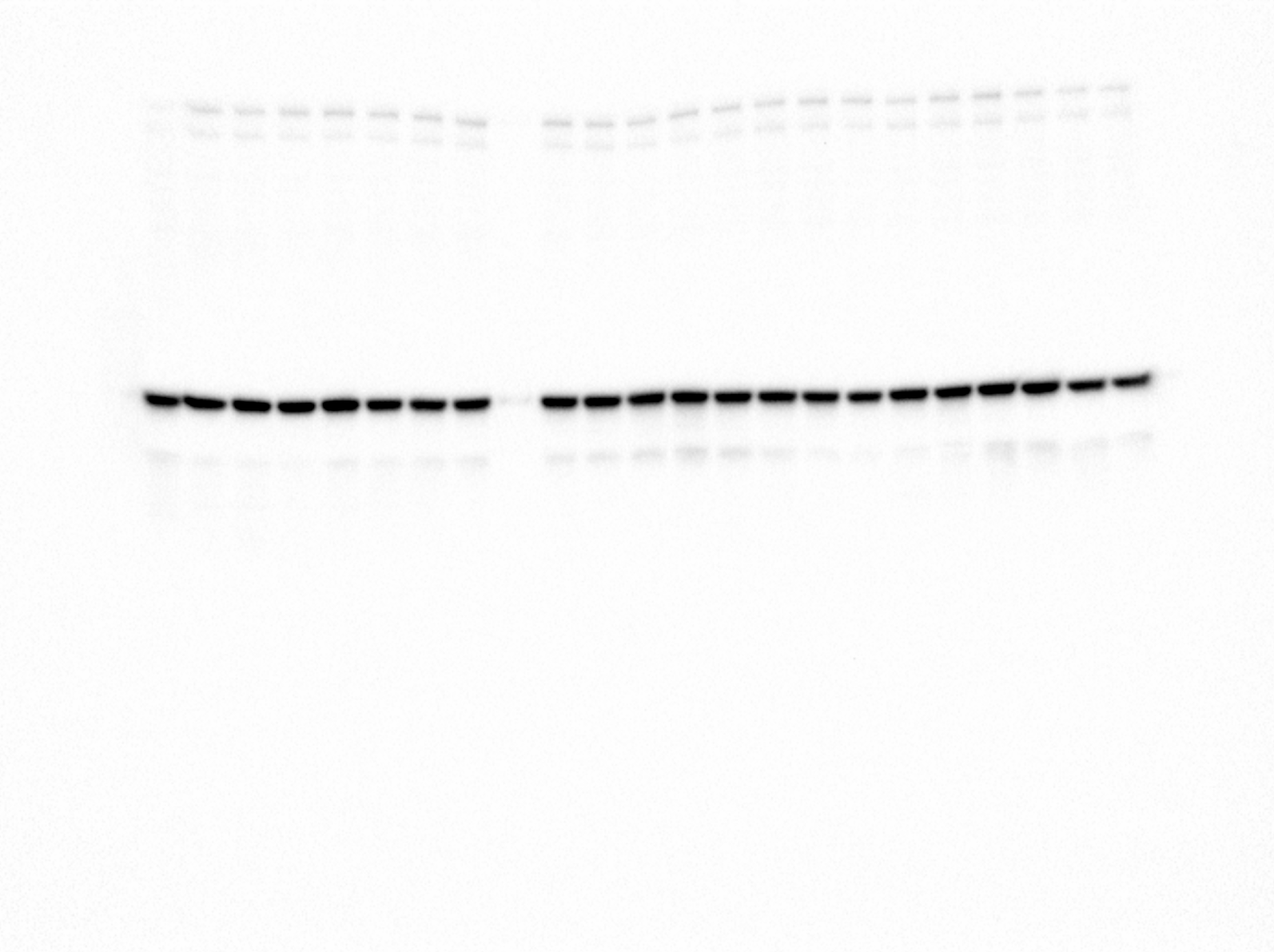

Supplement: Supplementary file 8 — Source data Fig. 1 [file 44320_2024_47_MOESM8_ESM.zip › Source Data for Figure 1/1E/CpG/tubulin gel 1/Image 2021-05-24 12hr 49min_Exposure_4.0sec.tif]

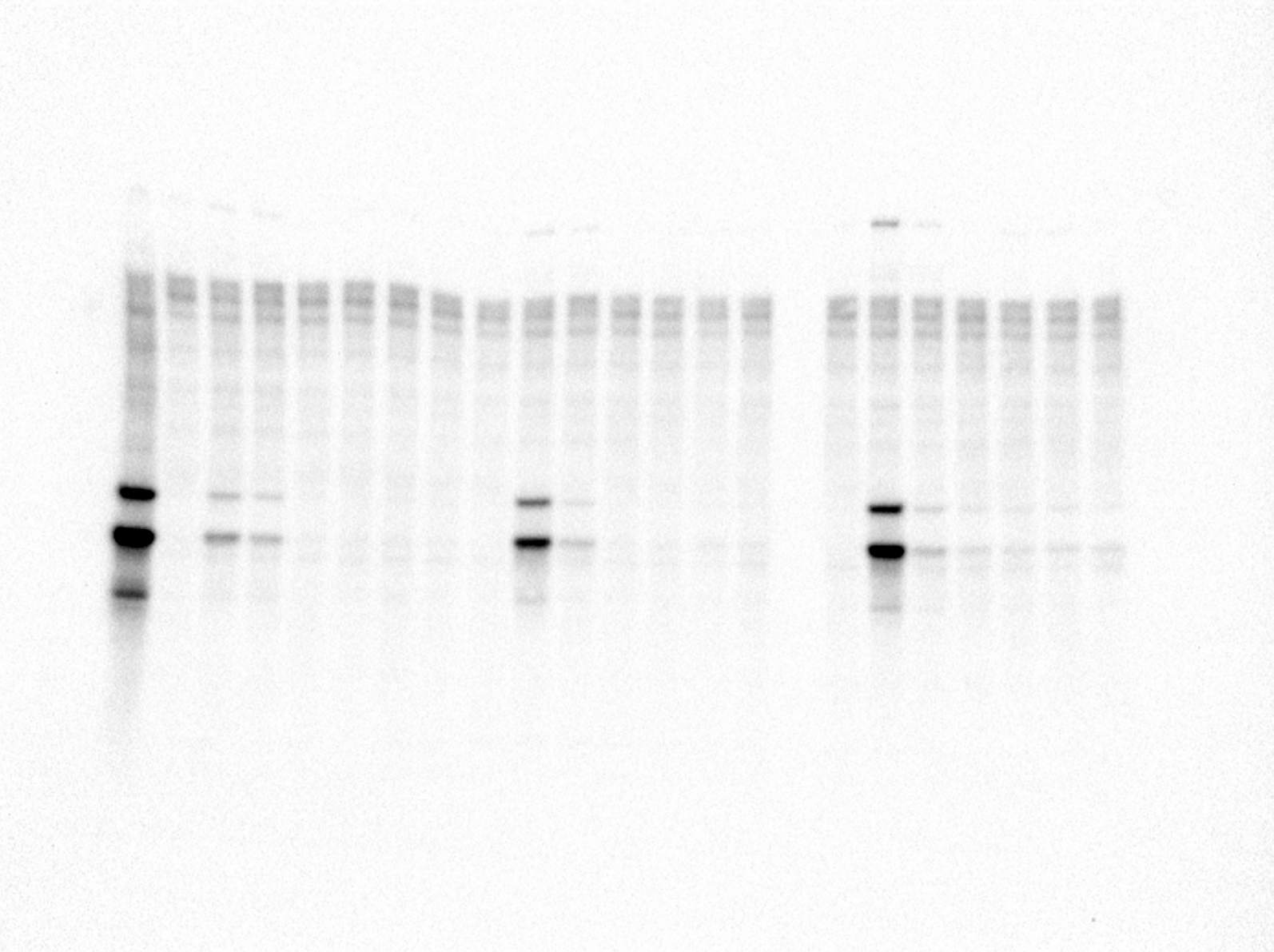

Supplement: Supplementary file 8 — Source data Fig. 1 [file 44320_2024_47_MOESM8_ESM.zip › Source Data for Figure 1/1E/TNF/pMK2/Image 2021-04-27 13hr 08min_Exposure_50.0sec.tif]

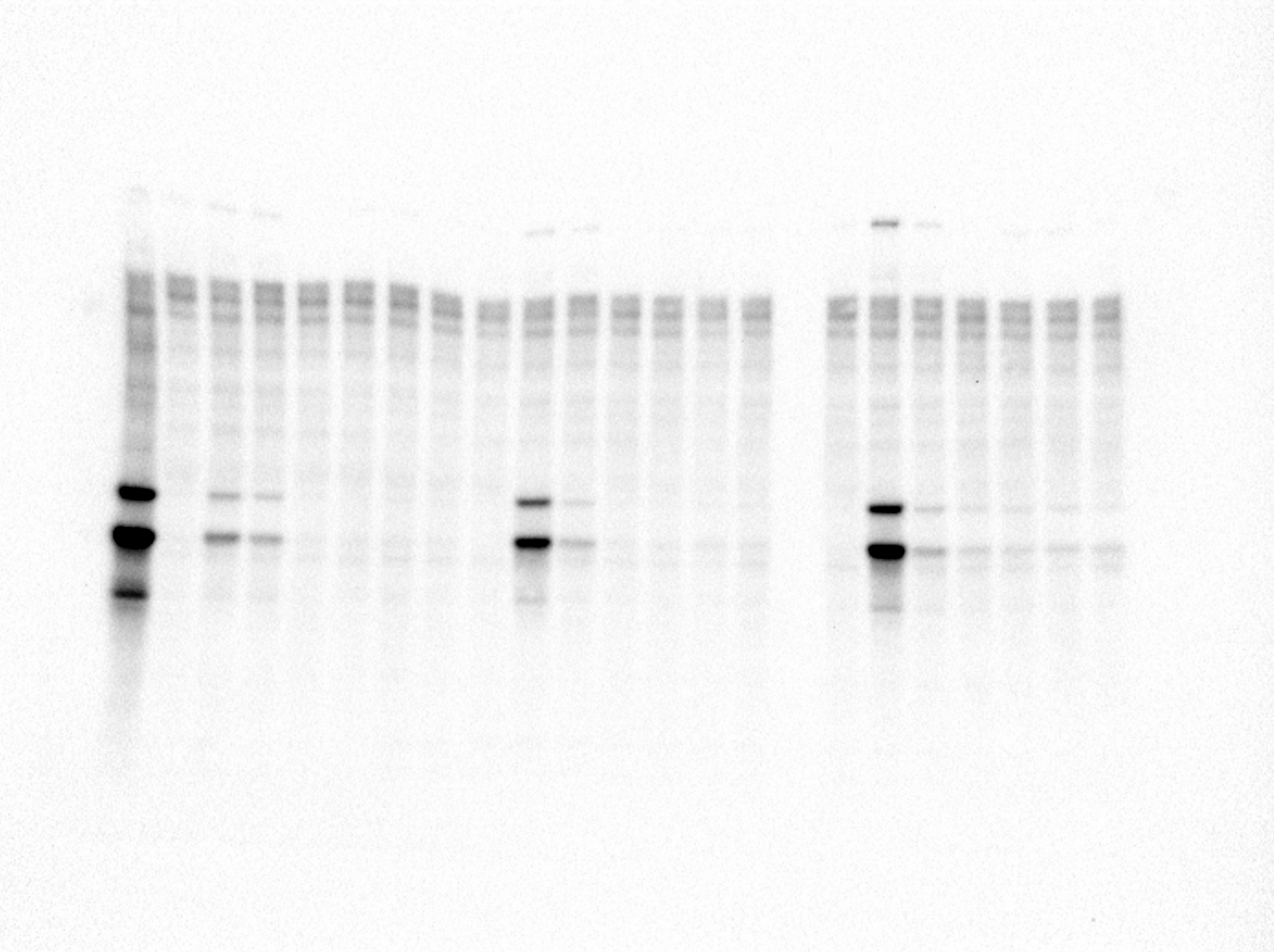

Supplement: Supplementary file 8 — Source data Fig. 1 [file 44320_2024_47_MOESM8_ESM.zip › Source Data for Figure 1/1E/TNF/pMK2/Image 2021-04-27 13hr 08min_Exposure_80.0sec.tif]

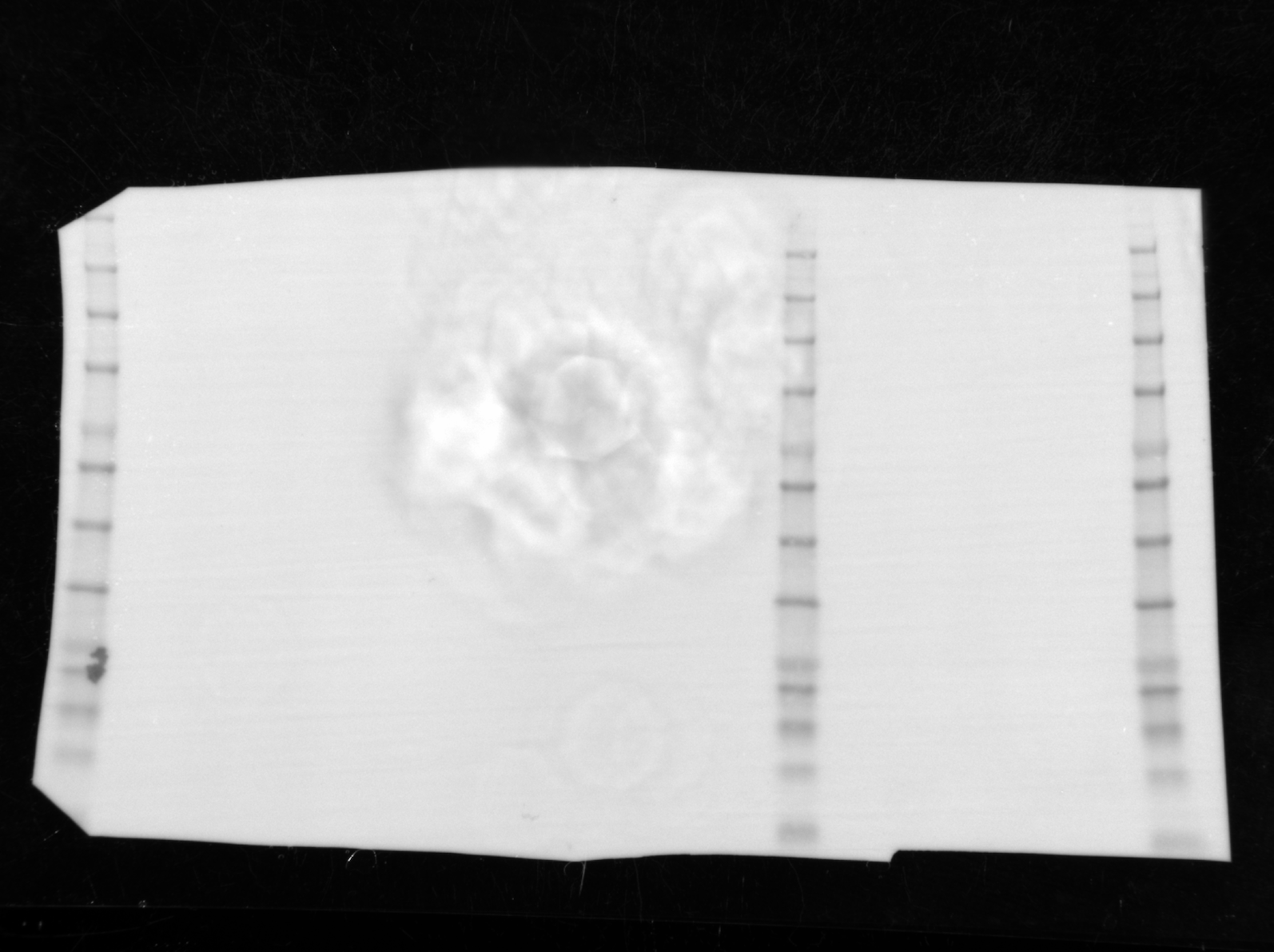

Supplement: Supplementary file 8 — Source data Fig. 1 [file 44320_2024_47_MOESM8_ESM.zip › Source Data for Figure 1/1E/TNF/pMK2/Image 2021-04-27 13hr 06min colorimetric.tif]

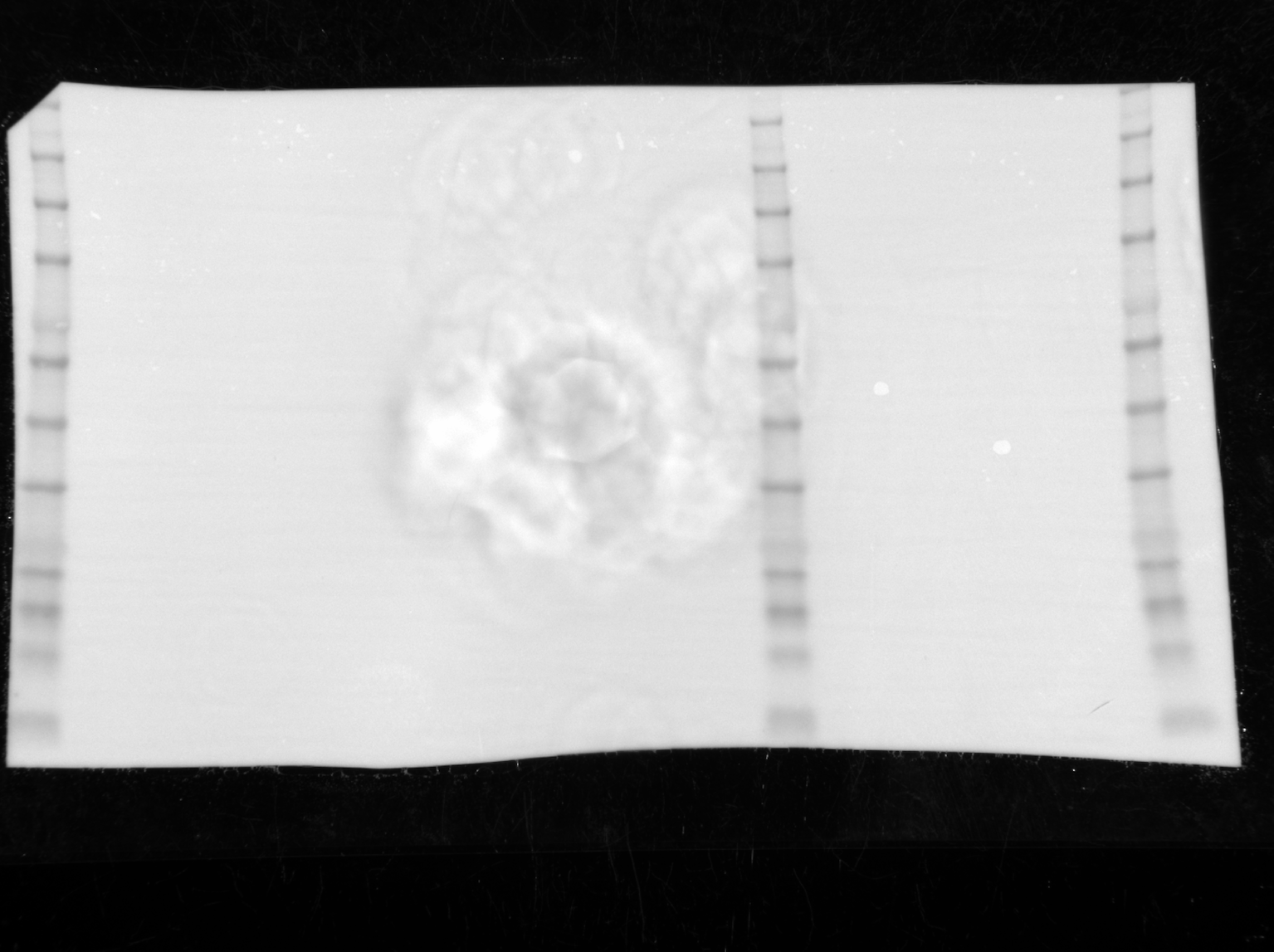

Supplement: Supplementary file 8 — Source data Fig. 1 [file 44320_2024_47_MOESM8_ESM.zip › Source Data for Figure 1/1E/TNF/tubulin gel1/Image 2021-04-30 10hr 21min colorimetric.tif]

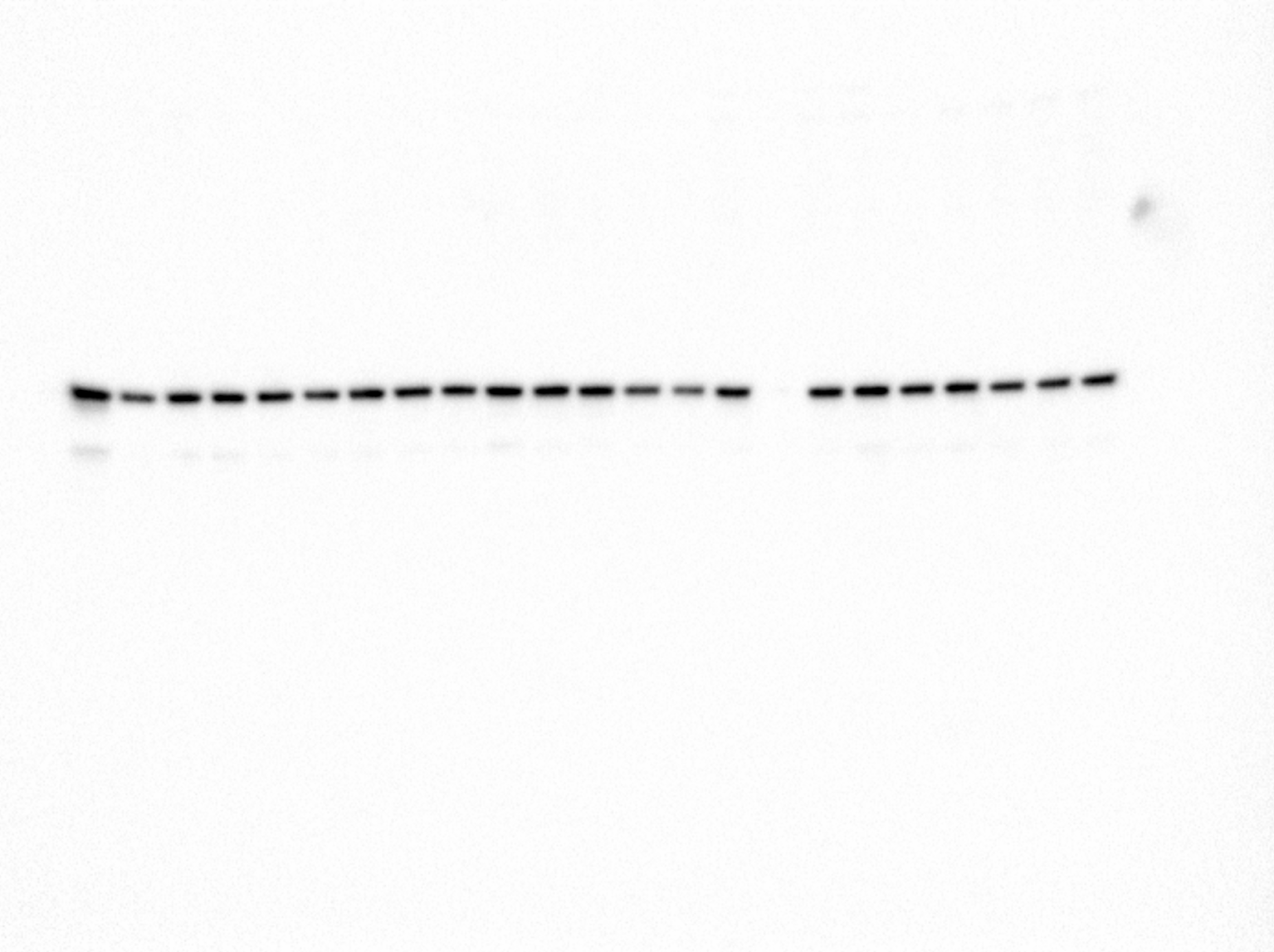

Supplement: Supplementary file 8 — Source data Fig. 1 [file 44320_2024_47_MOESM8_ESM.zip › Source Data for Figure 1/1E/TNF/tubulin gel1/Image 2021-04-30 10hr 23min_Exposure_18.0sec.tif]

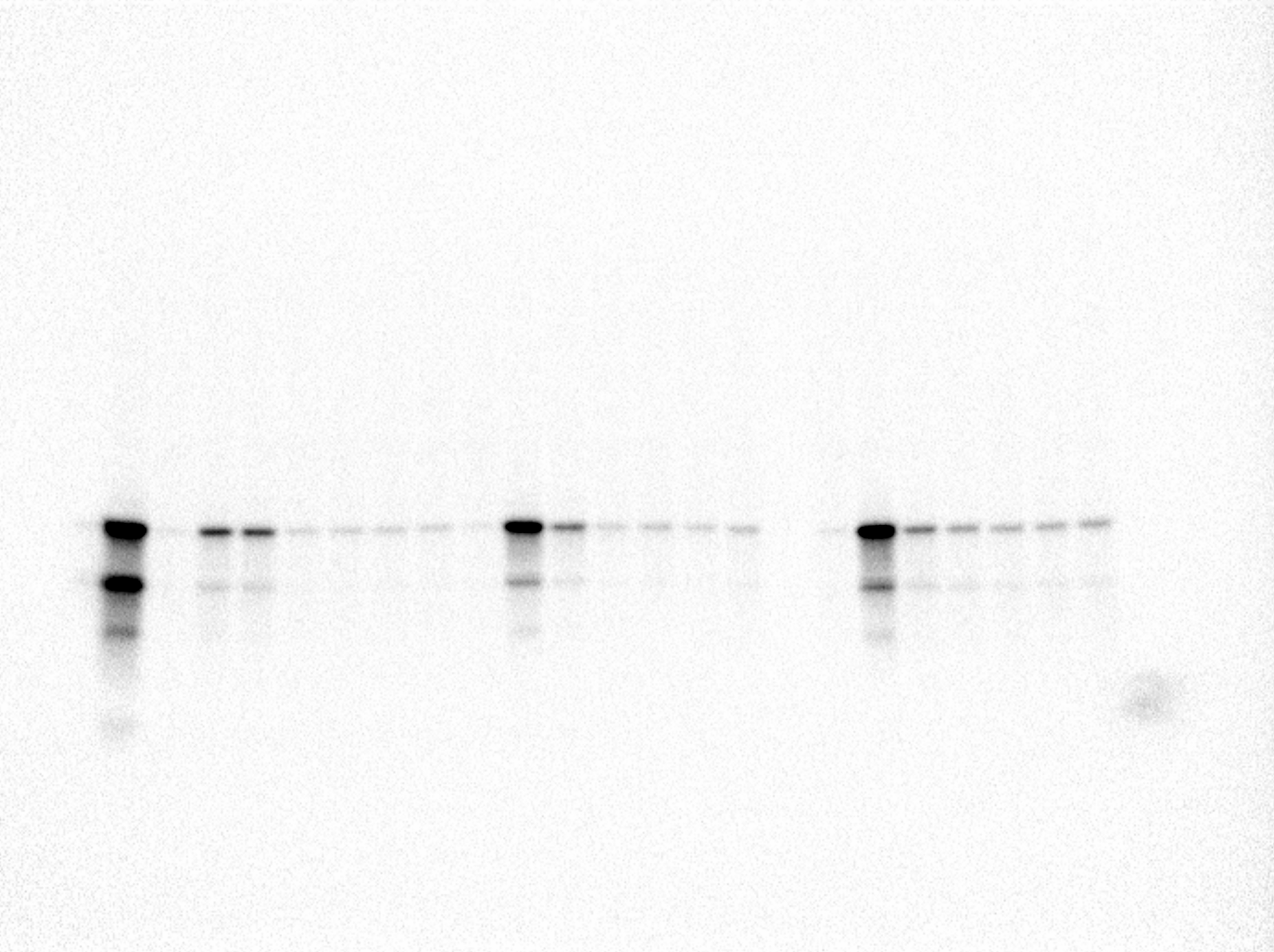

Supplement: Supplementary file 8 — Source data Fig. 1 [file 44320_2024_47_MOESM8_ESM.zip › Source Data for Figure 1/1E/TNF/pp38/Image 2021-04-28 12hr 22min_Exposure_18.0sec.tif]

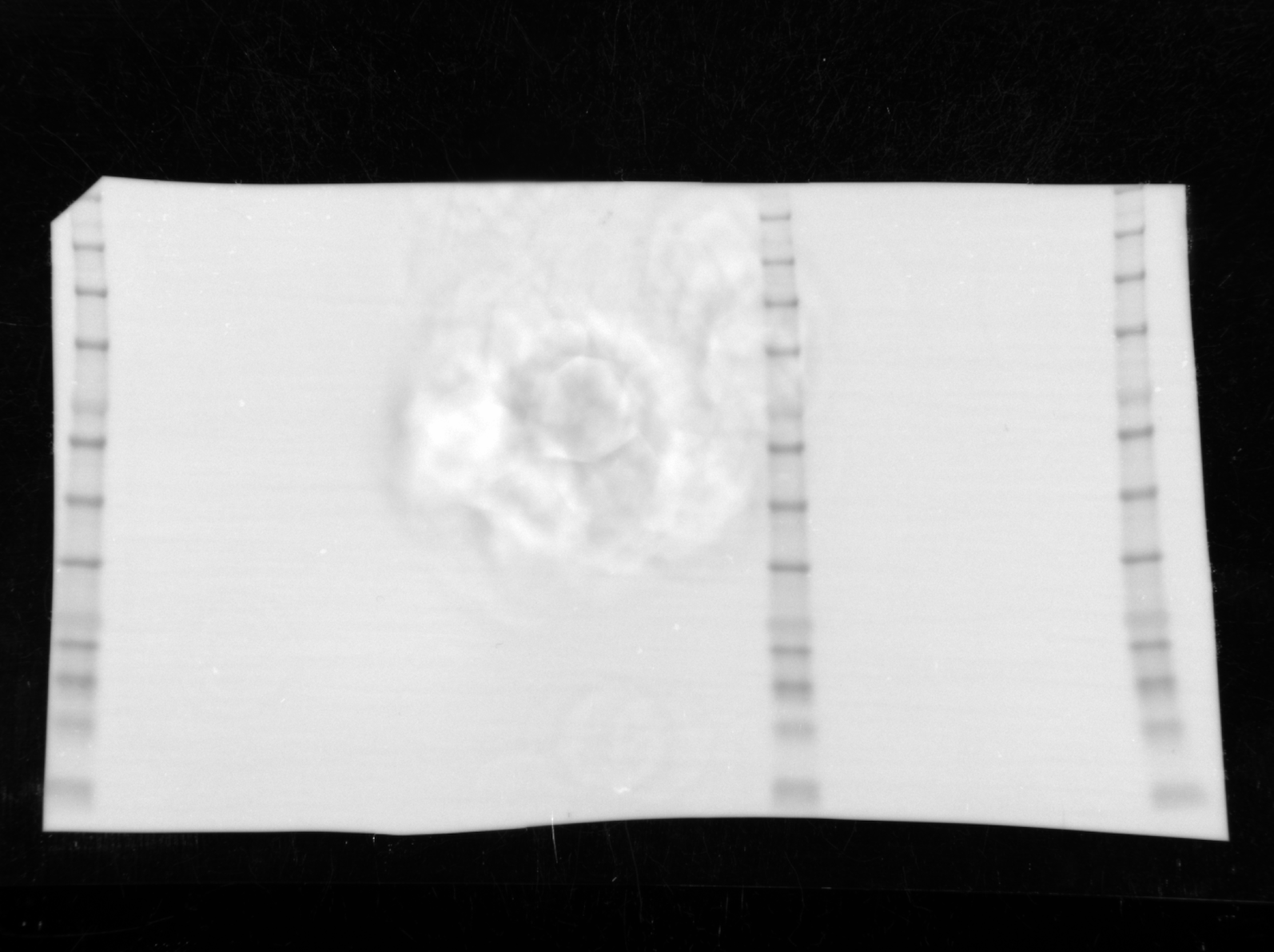

Supplement: Supplementary file 8 — Source data Fig. 1 [file 44320_2024_47_MOESM8_ESM.zip › Source Data for Figure 1/1E/TNF/pp38/Image 2021-04-28 12hr 20min colorimetric.tif]

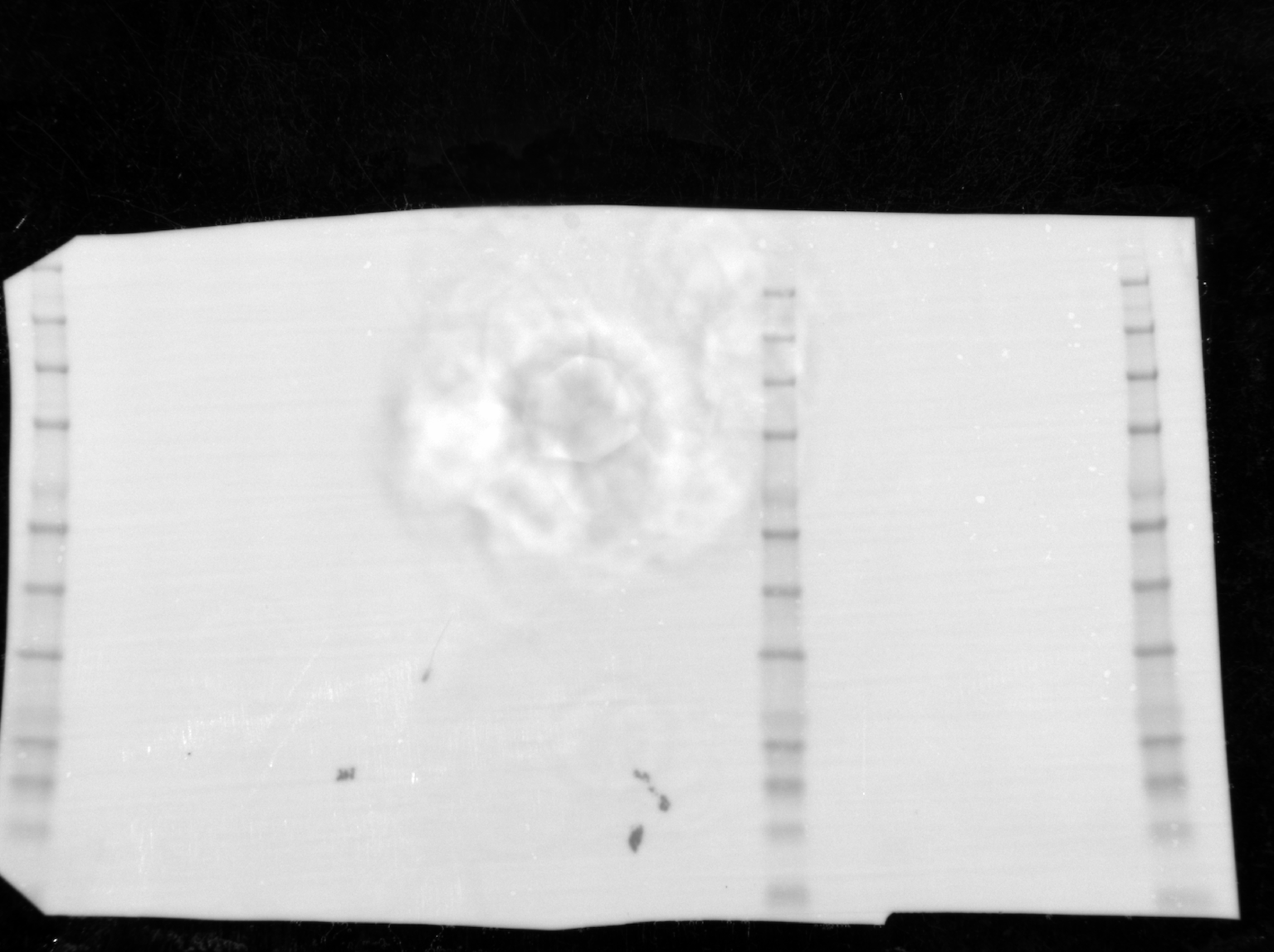

Supplement: Supplementary file 8 — Source data Fig. 1 [file 44320_2024_47_MOESM8_ESM.zip › Source Data for Figure 1/1E/TNF/tubulin gel2/Image 2021-04-30 10hr 38min colorimtetric.tif]

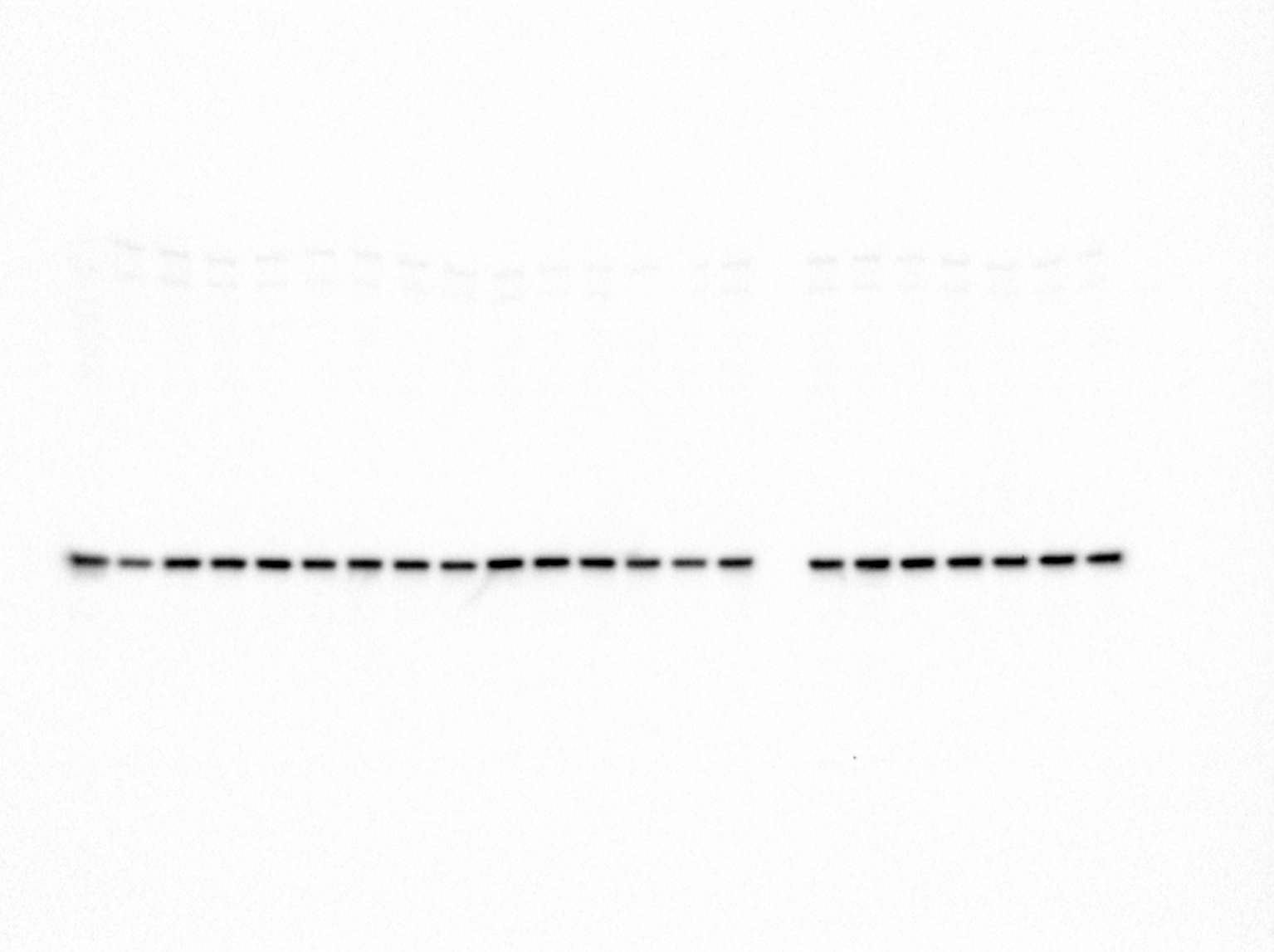

Supplement: Supplementary file 8 — Source data Fig. 1 [file 44320_2024_47_MOESM8_ESM.zip › Source Data for Figure 1/1E/TNF/tubulin gel2/Image 2021-04-30 10hr 40min_Exposure_14.0sec.tif]

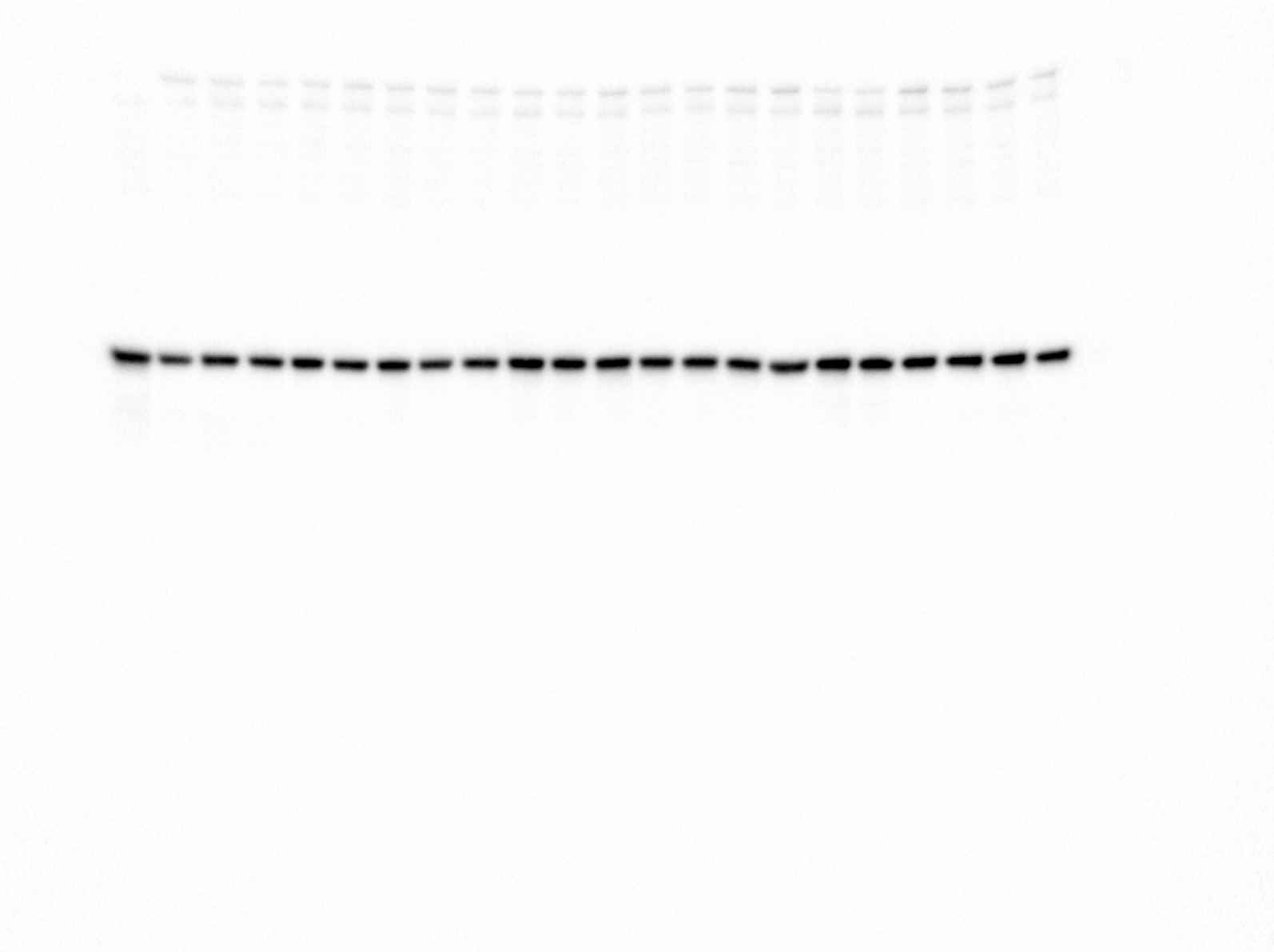

Supplement: Supplementary file 8 — Source data Fig. 1 [file 44320_2024_47_MOESM8_ESM.zip › Source Data for Figure 1/1E/P3C4/tubulin gel 2/Image 2021-05-24 12hr 57min_Exposure_4.0sec.tif]

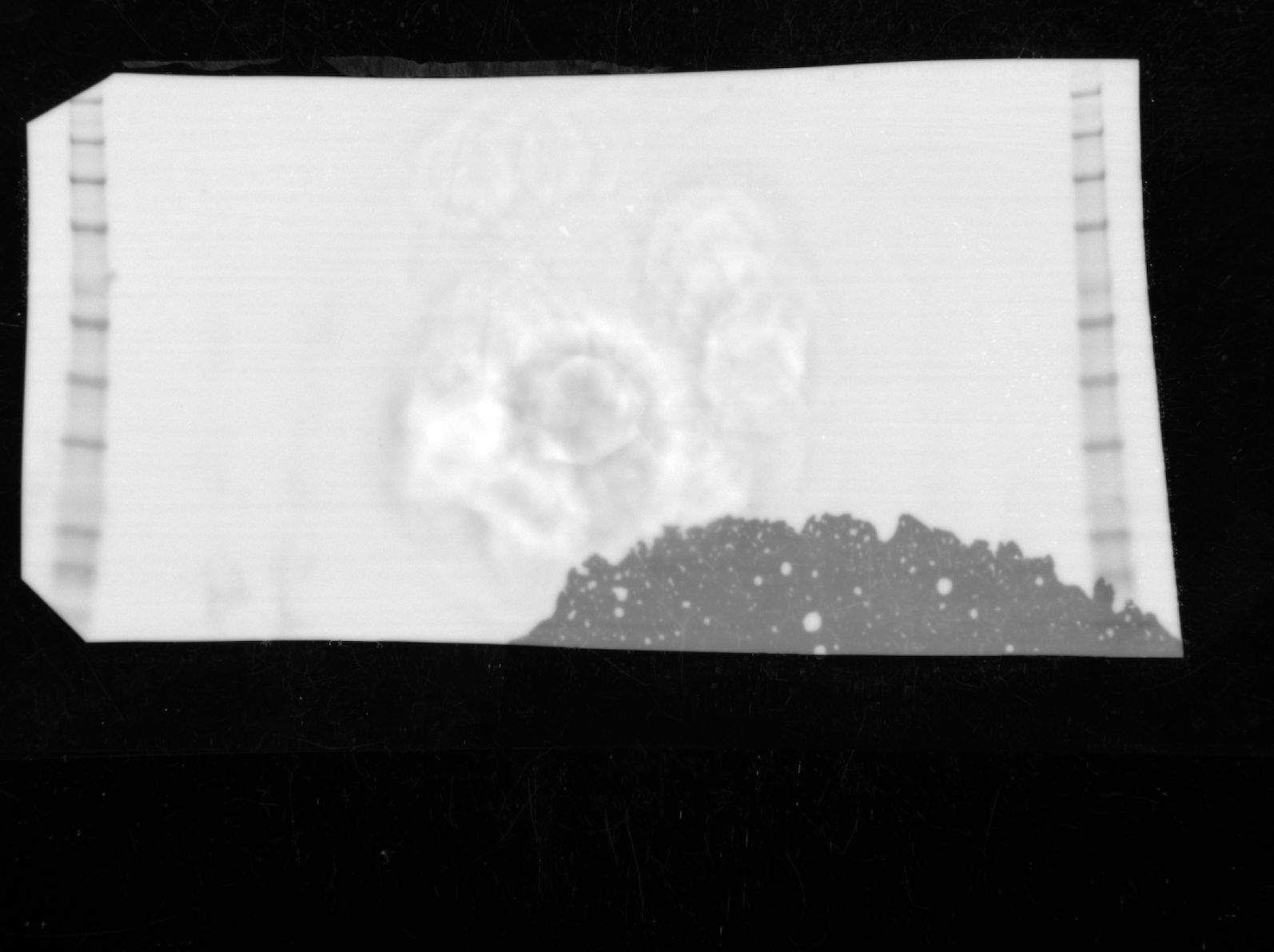

Supplement: Supplementary file 8 — Source data Fig. 1 [file 44320_2024_47_MOESM8_ESM.zip › Source Data for Figure 1/1E/P3C4/tubulin gel 2/Image 2021-05-24 13hr 00min colorimetric.tif]

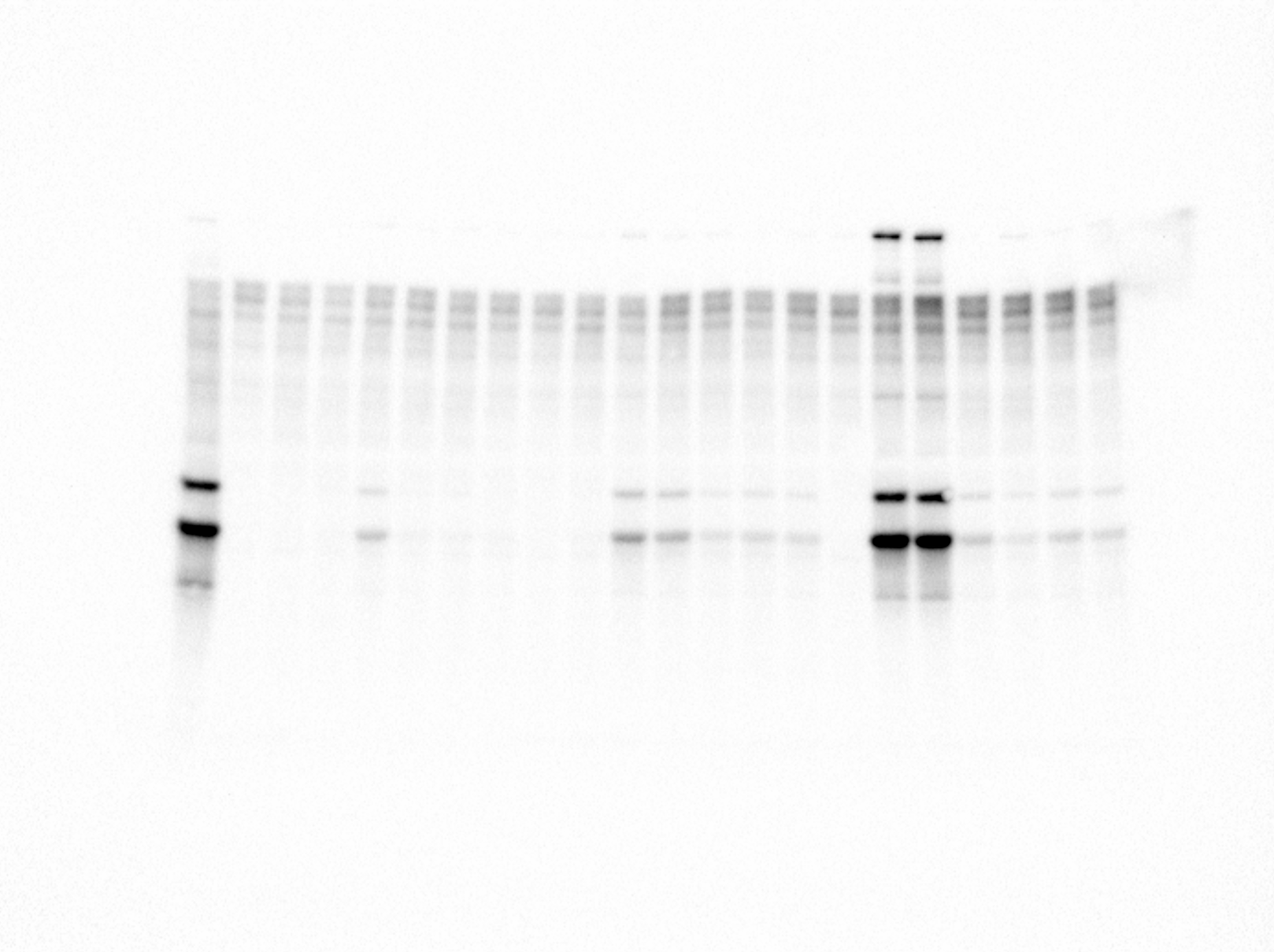

Supplement: Supplementary file 8 — Source data Fig. 1 [file 44320_2024_47_MOESM8_ESM.zip › Source Data for Figure 1/1E/P3C4/pMK2/Image 2021-05-18 12hr 17min_Exposure_30.0sec.tif]

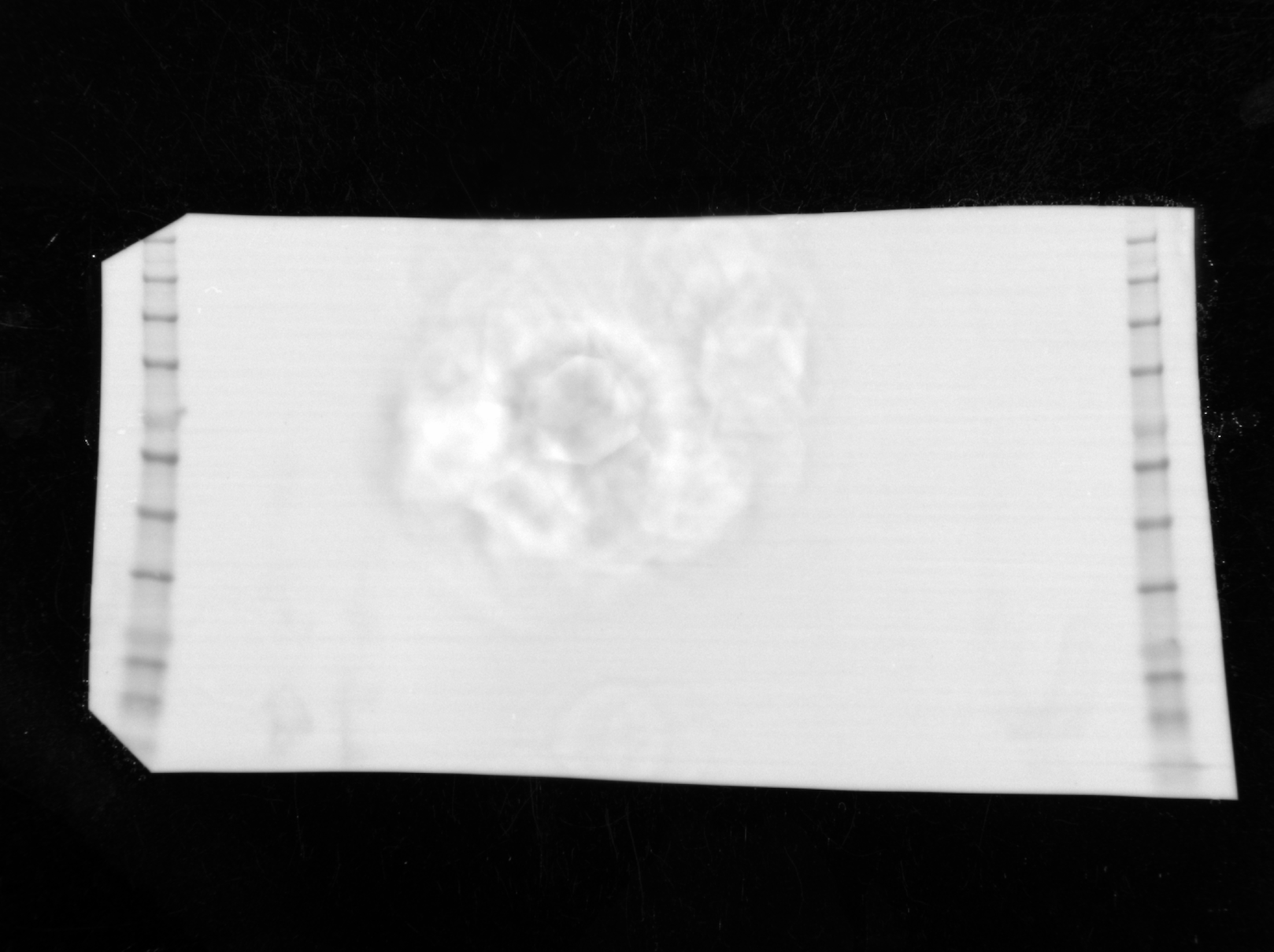

Supplement: Supplementary file 8 — Source data Fig. 1 [file 44320_2024_47_MOESM8_ESM.zip › Source Data for Figure 1/1E/P3C4/pMK2/Image 2021-05-18 12hr 27min colorimetric.tif]

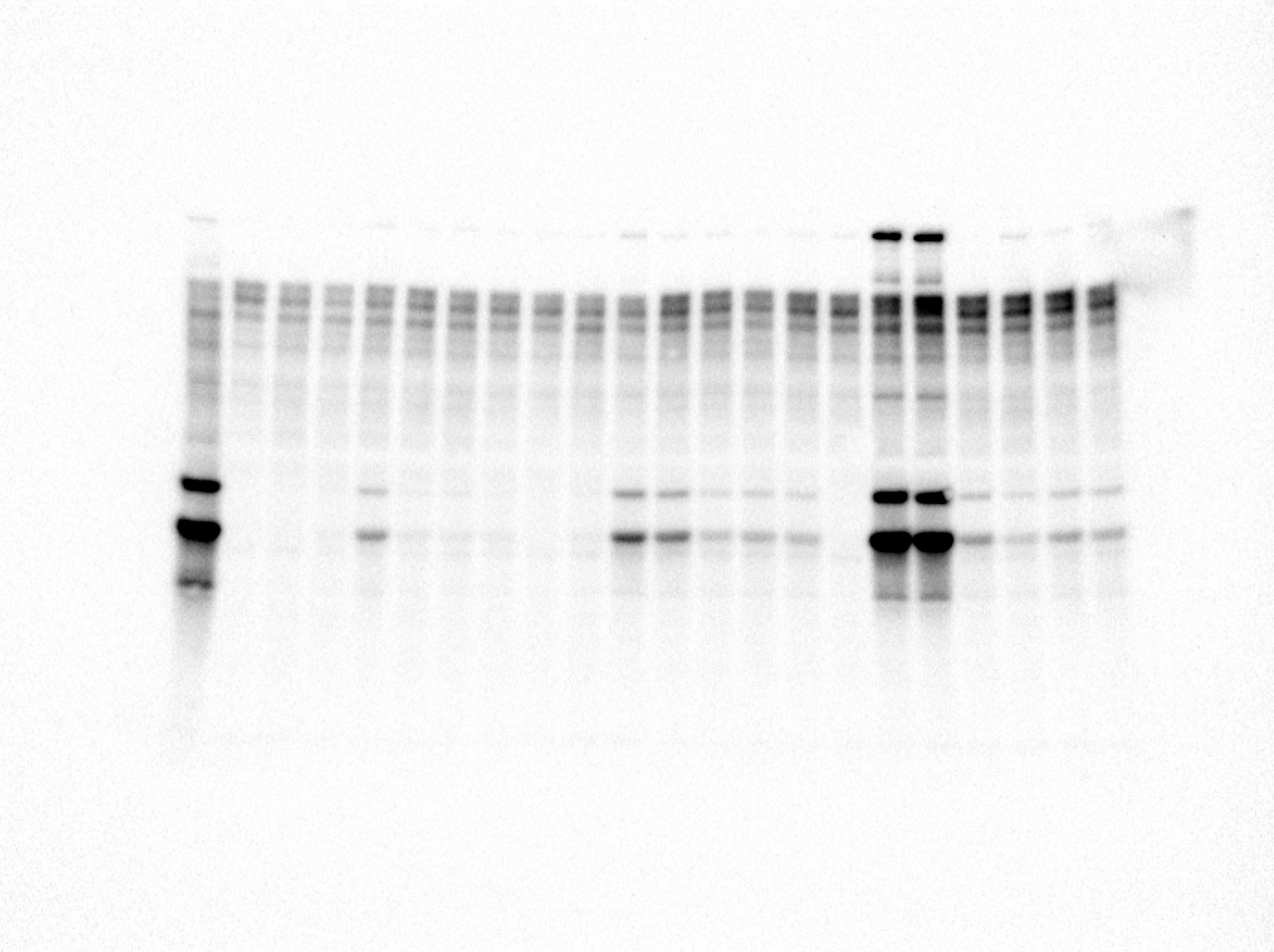

Supplement: Supplementary file 8 — Source data Fig. 1 [file 44320_2024_47_MOESM8_ESM.zip › Source Data for Figure 1/1E/P3C4/pMK2/Image 2021-05-18 12hr 17min_Exposure_50.0sec w overexposure.tif]

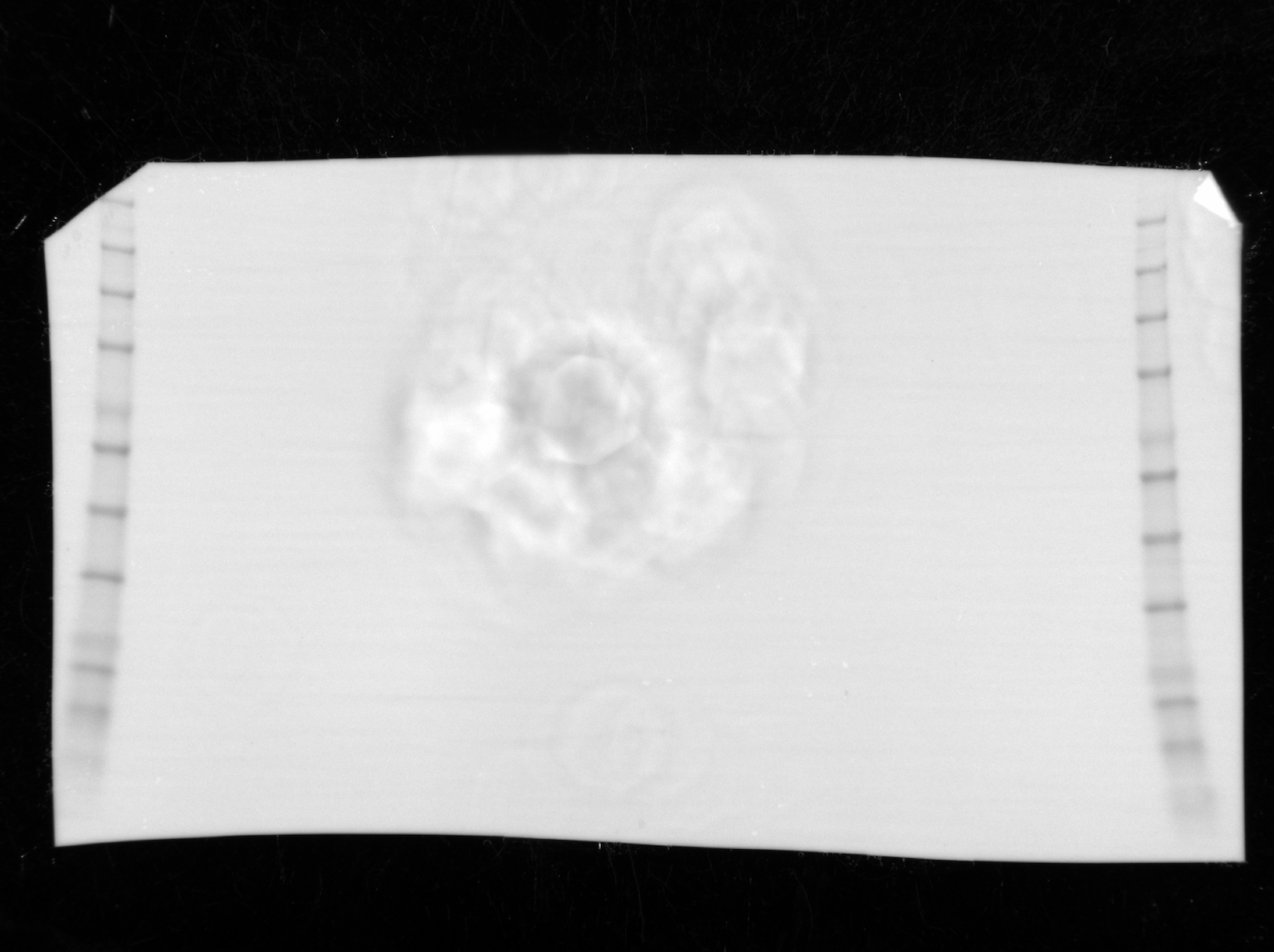

Supplement: Supplementary file 8 — Source data Fig. 1 [file 44320_2024_47_MOESM8_ESM.zip › Source Data for Figure 1/1E/P3C4/pp38/Image 2021-05-19 11hr 53min colorimetric.tif]

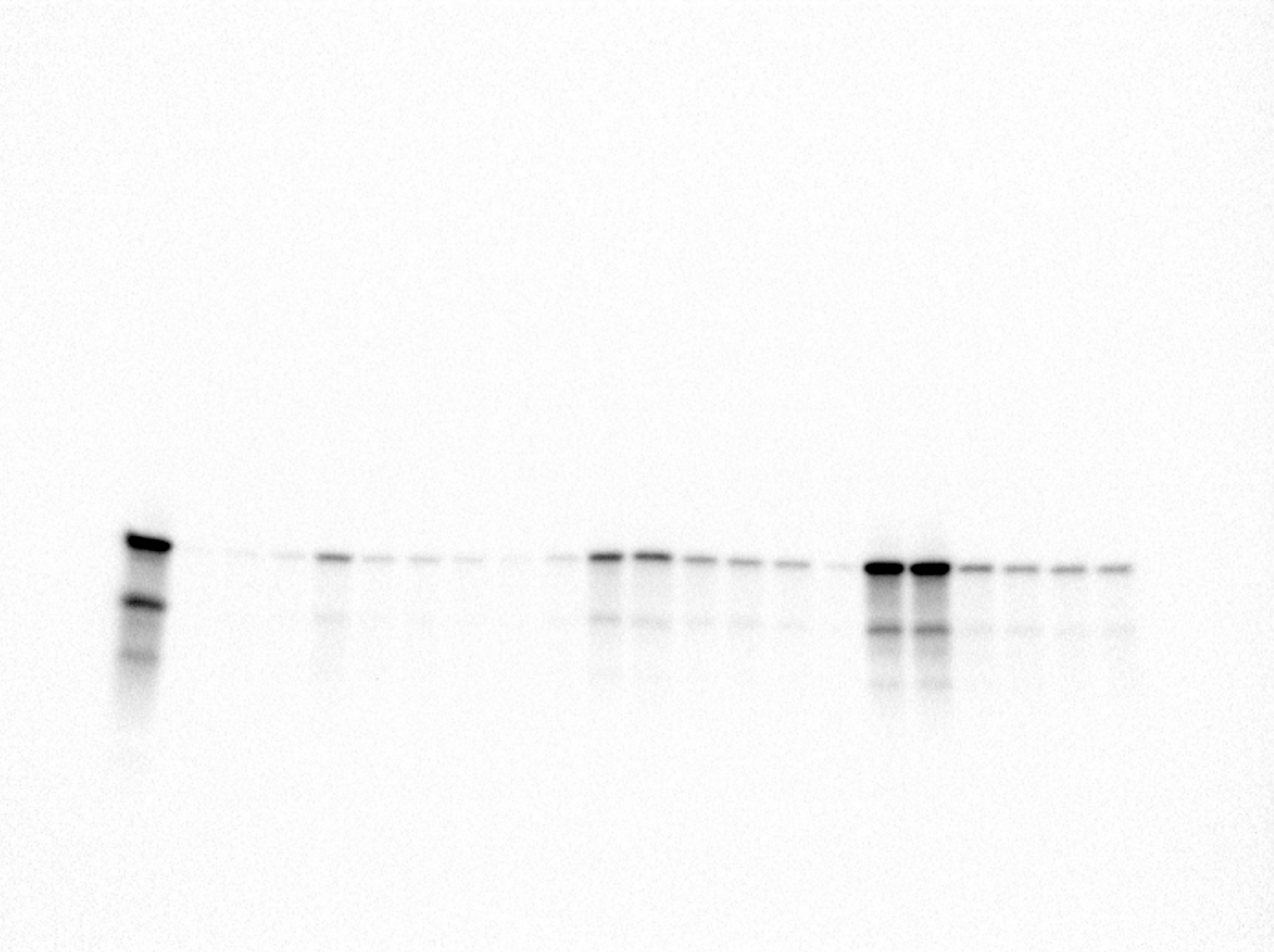

Supplement: Supplementary file 8 — Source data Fig. 1 [file 44320_2024_47_MOESM8_ESM.zip › Source Data for Figure 1/1E/P3C4/pp38/Image 2021-05-19 11hr 51min_Exposure_10.0sec.tif]

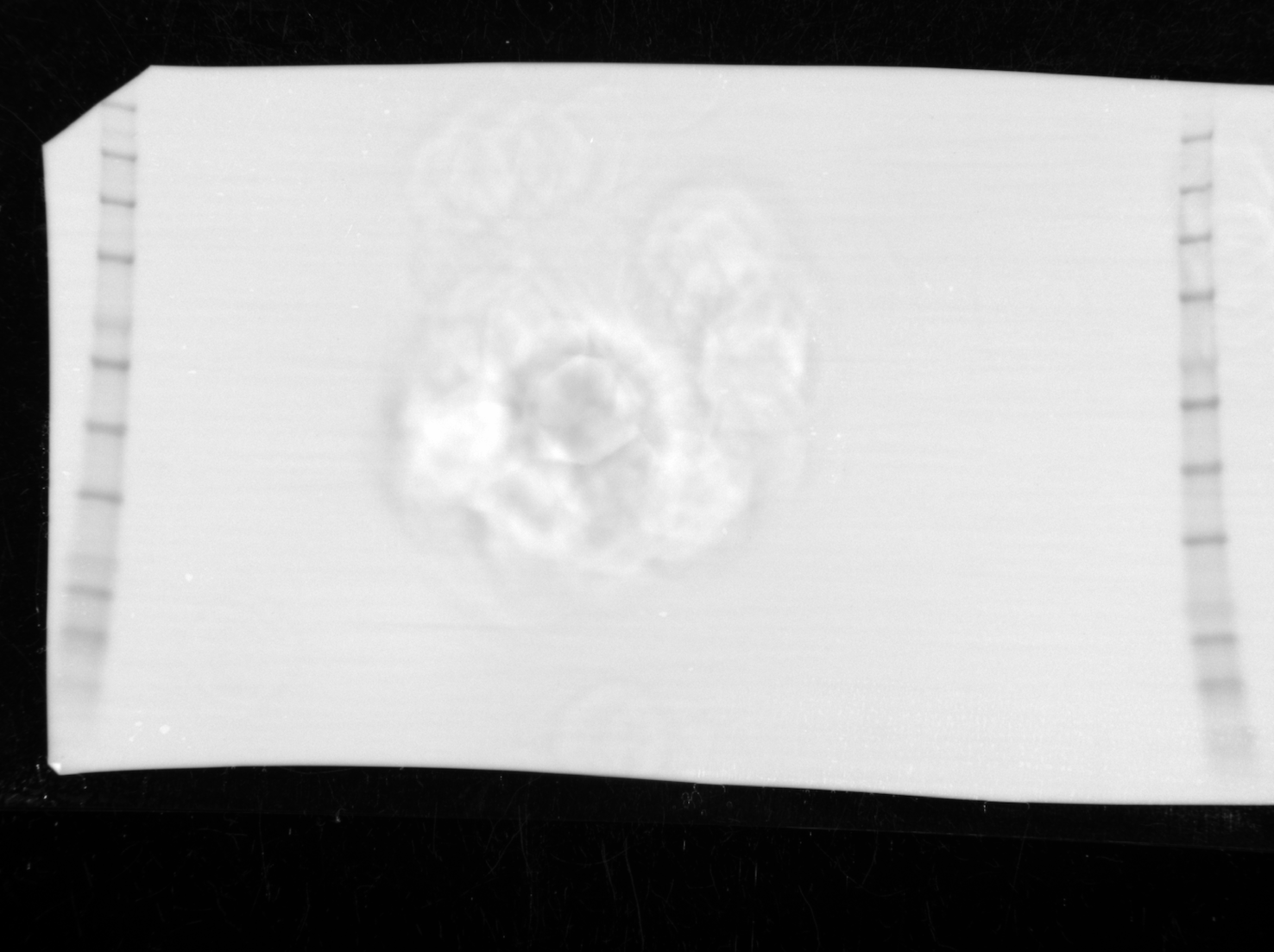

Supplement: Supplementary file 8 — Source data Fig. 1 [file 44320_2024_47_MOESM8_ESM.zip › Source Data for Figure 1/1E/P3C4/tubulin gel 1/Image 2021-05-24 12hr 43min colorimetric.tif]

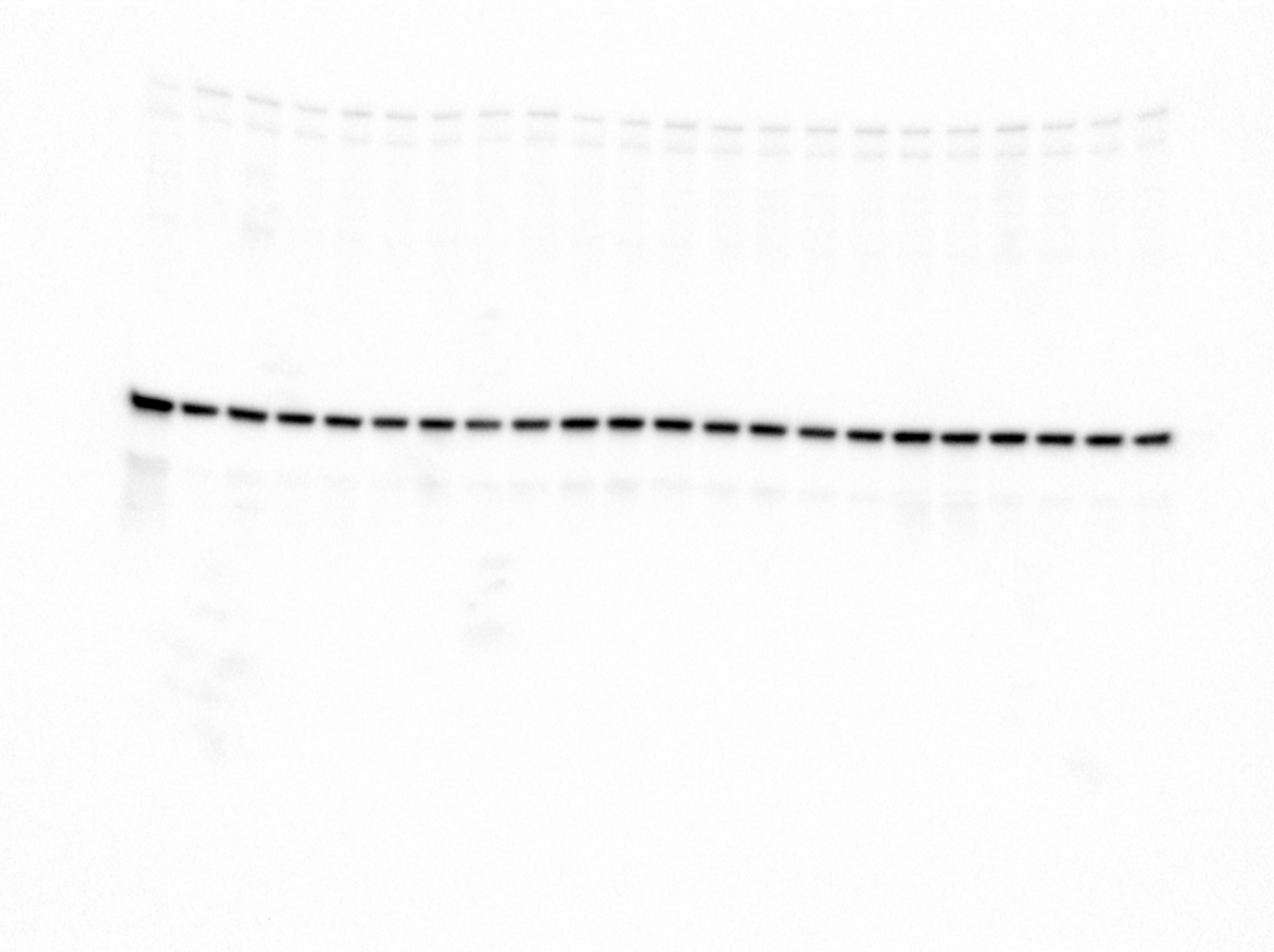

Supplement: Supplementary file 8 — Source data Fig. 1 [file 44320_2024_47_MOESM8_ESM.zip › Source Data for Figure 1/1E/P3C4/tubulin gel 1/Image 2021-05-24 12hr 40min_Exposure_4.0sec.tif]

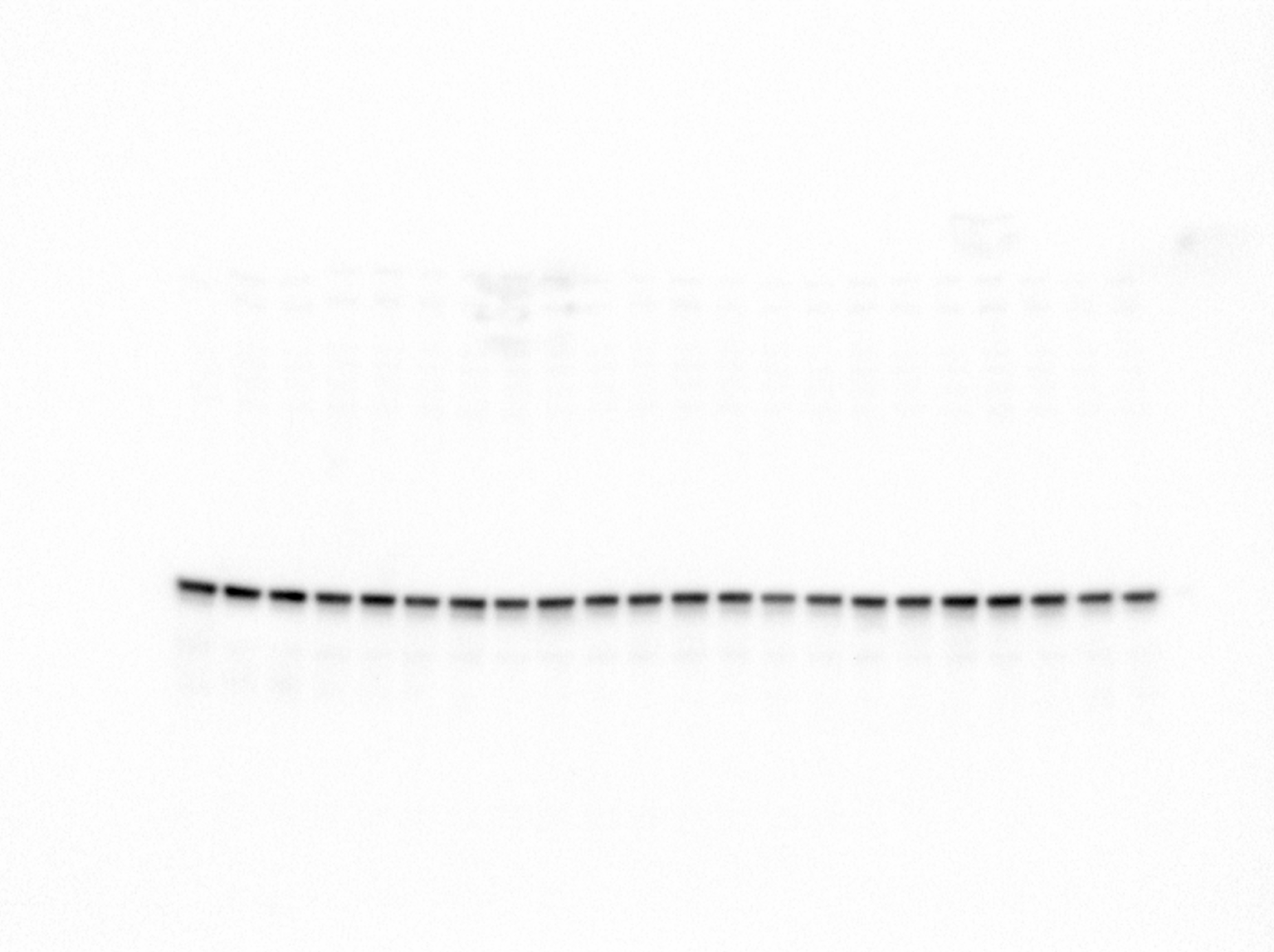

Supplement: Supplementary file 8 — Source data Fig. 1 [file 44320_2024_47_MOESM8_ESM.zip › Source Data for Figure 1/1E/LPS/tubulin gel 2/Image 2021-04-30 10hr 30min_Exposure_10.0sec.tif]

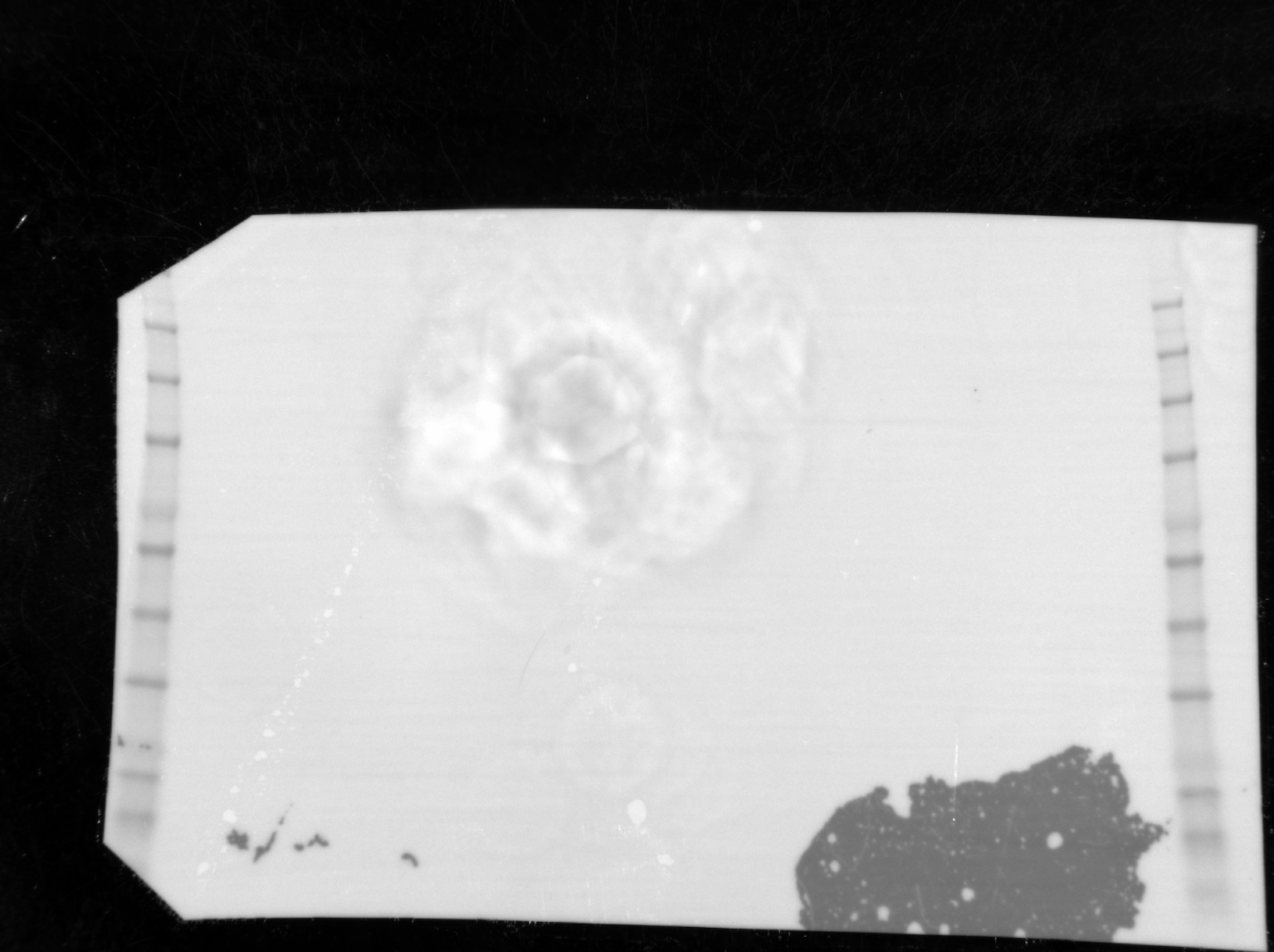

Supplement: Supplementary file 8 — Source data Fig. 1 [file 44320_2024_47_MOESM8_ESM.zip › Source Data for Figure 1/1E/LPS/tubulin gel 2/Image 2021-04-30 10hr 32min colorimetric.tif]

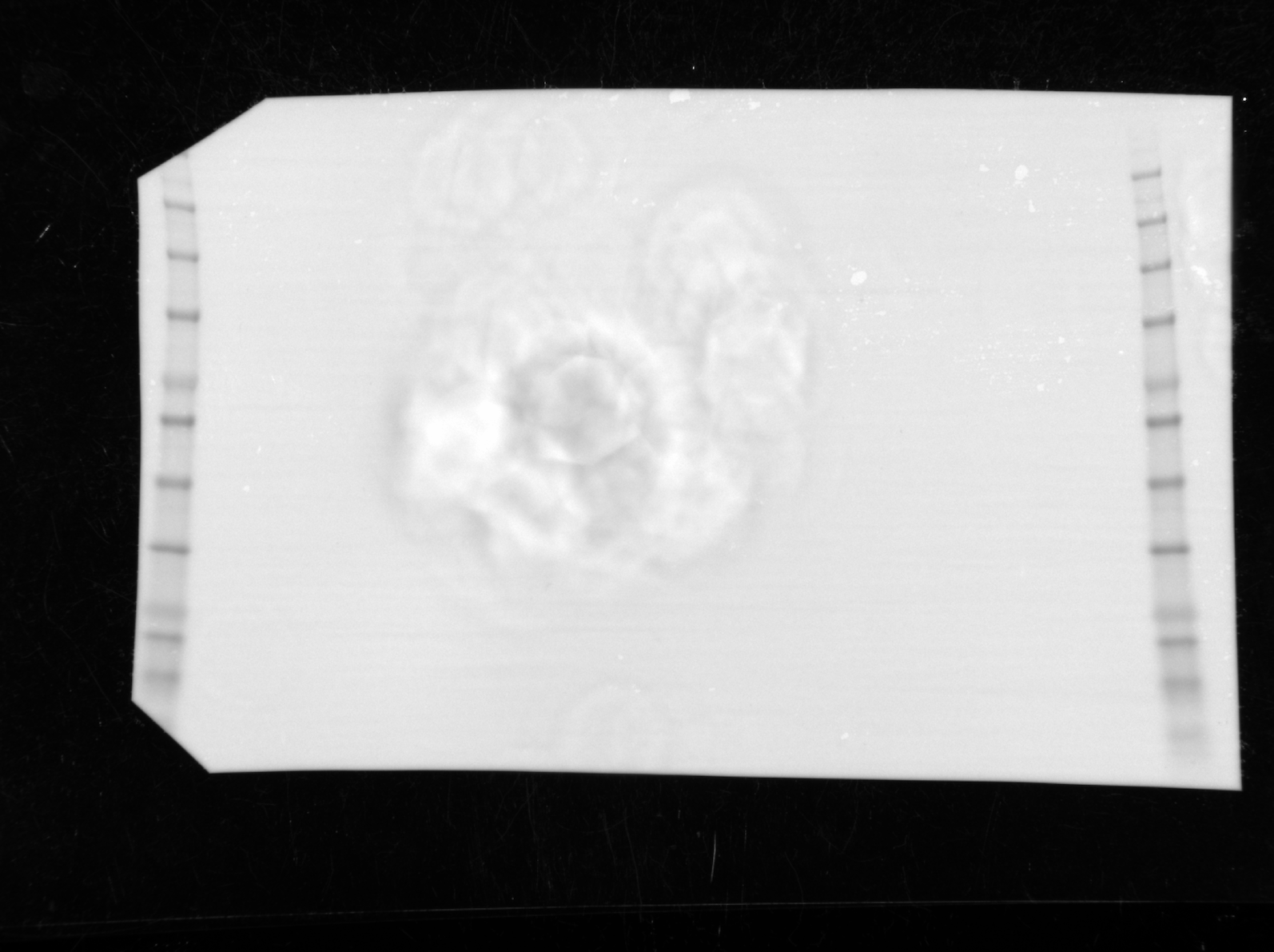

Supplement: Supplementary file 8 — Source data Fig. 1 [file 44320_2024_47_MOESM8_ESM.zip › Source Data for Figure 1/1E/LPS/pMK2/Image 2021-04-27 13hr 00min colorimetric.tif]

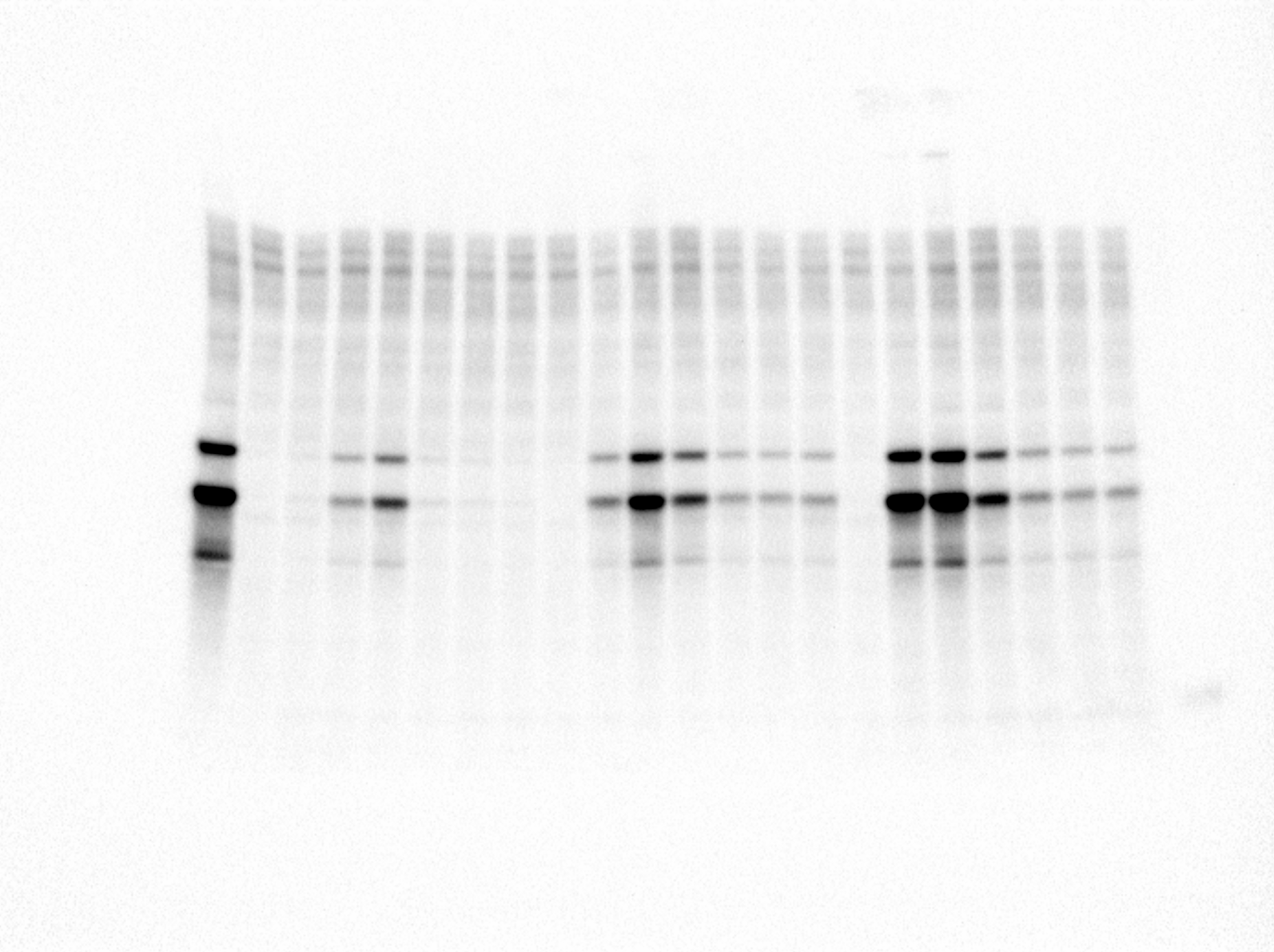

Supplement: Supplementary file 8 — Source data Fig. 1 [file 44320_2024_47_MOESM8_ESM.zip › Source Data for Figure 1/1E/LPS/pMK2/Image 2021-04-27 12hr 55min_Exposure_70.0sec.tif]

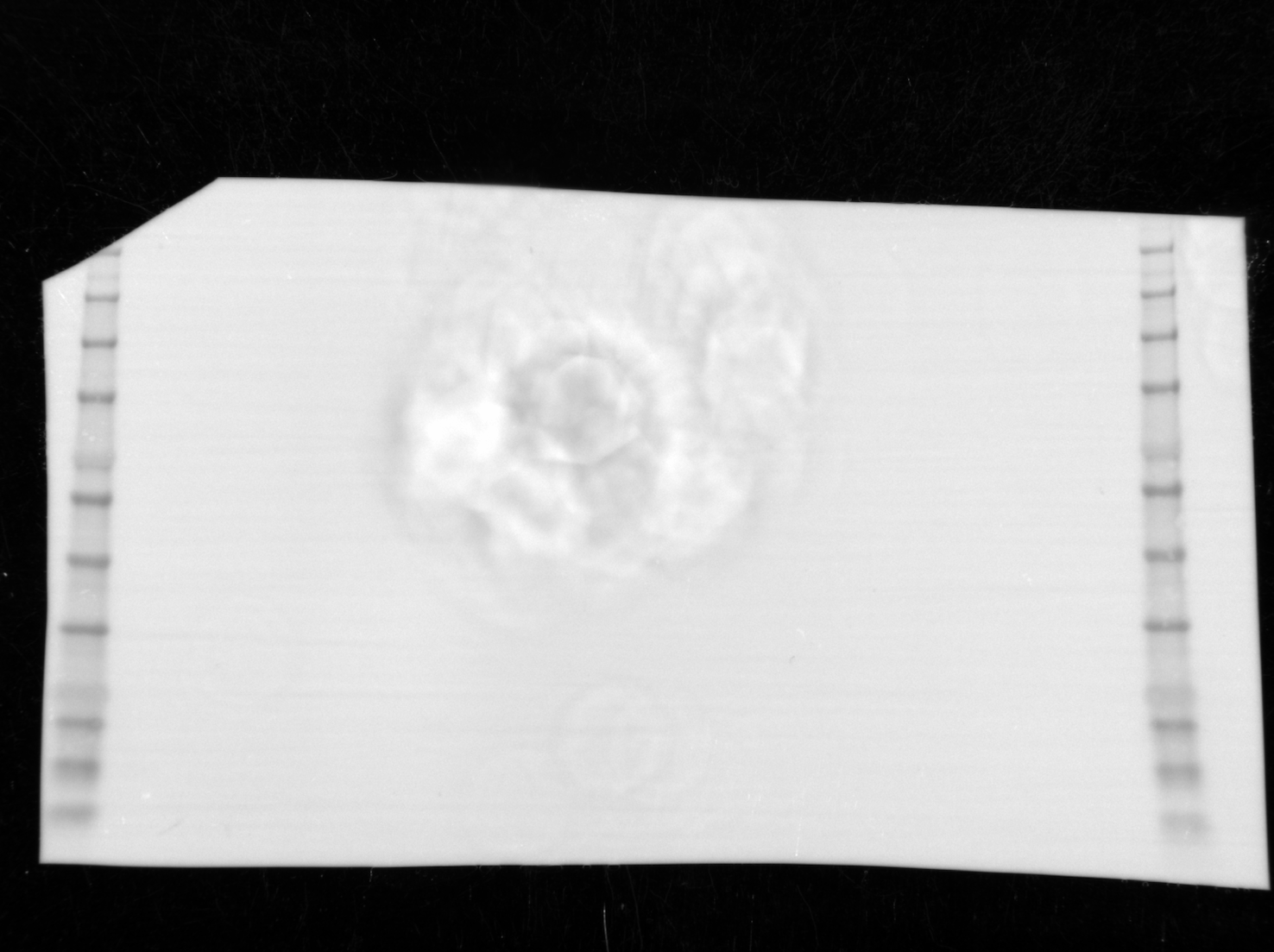

Supplement: Supplementary file 8 — Source data Fig. 1 [file 44320_2024_47_MOESM8_ESM.zip › Source Data for Figure 1/1E/LPS/pp38/Image 2021-04-28 12hr 12min colorimetricf.tif]

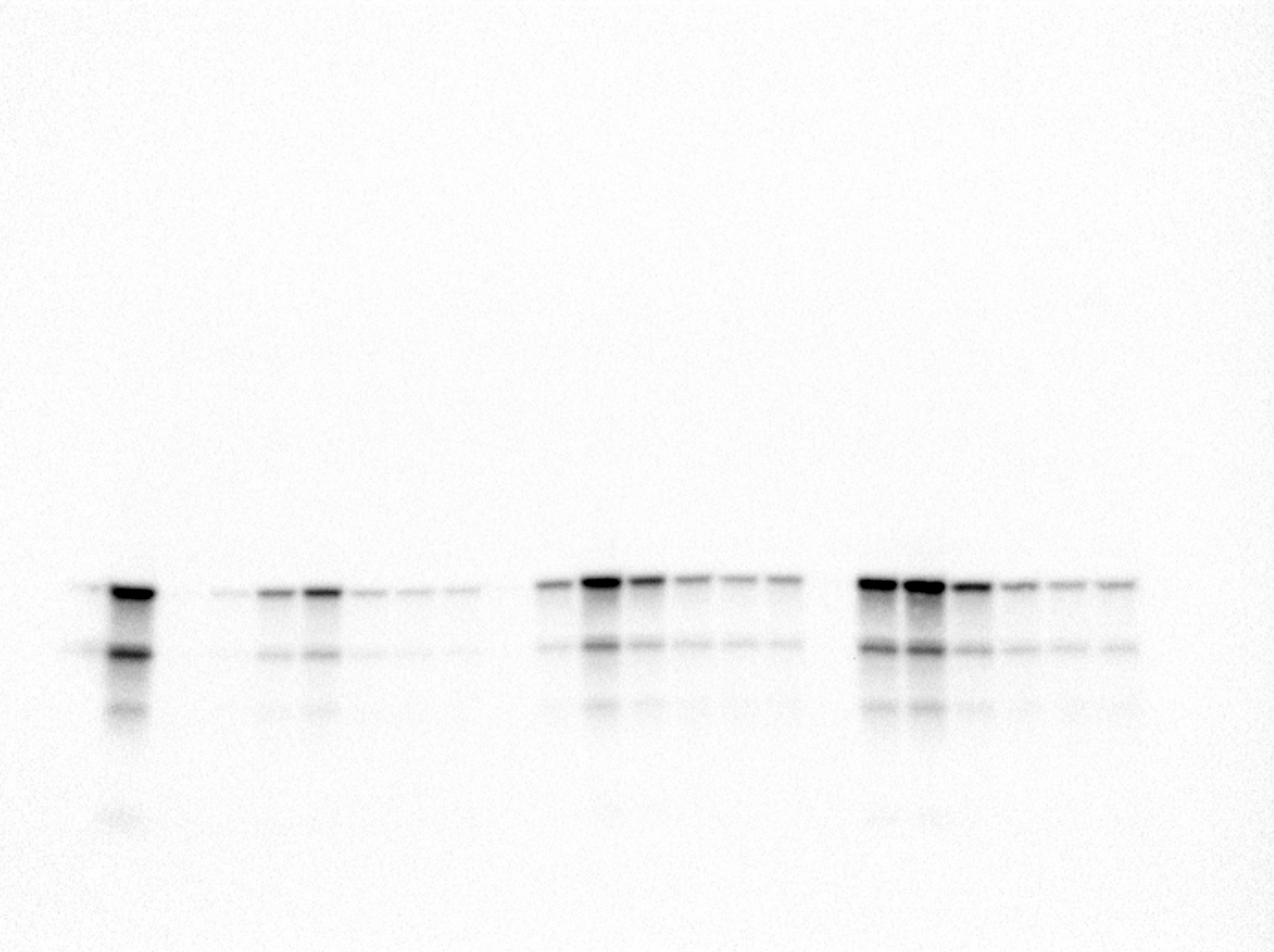

Supplement: Supplementary file 8 — Source data Fig. 1 [file 44320_2024_47_MOESM8_ESM.zip › Source Data for Figure 1/1E/LPS/pp38/Image 2021-04-28 12hr 09min_Exposure_14.0sec.tif]

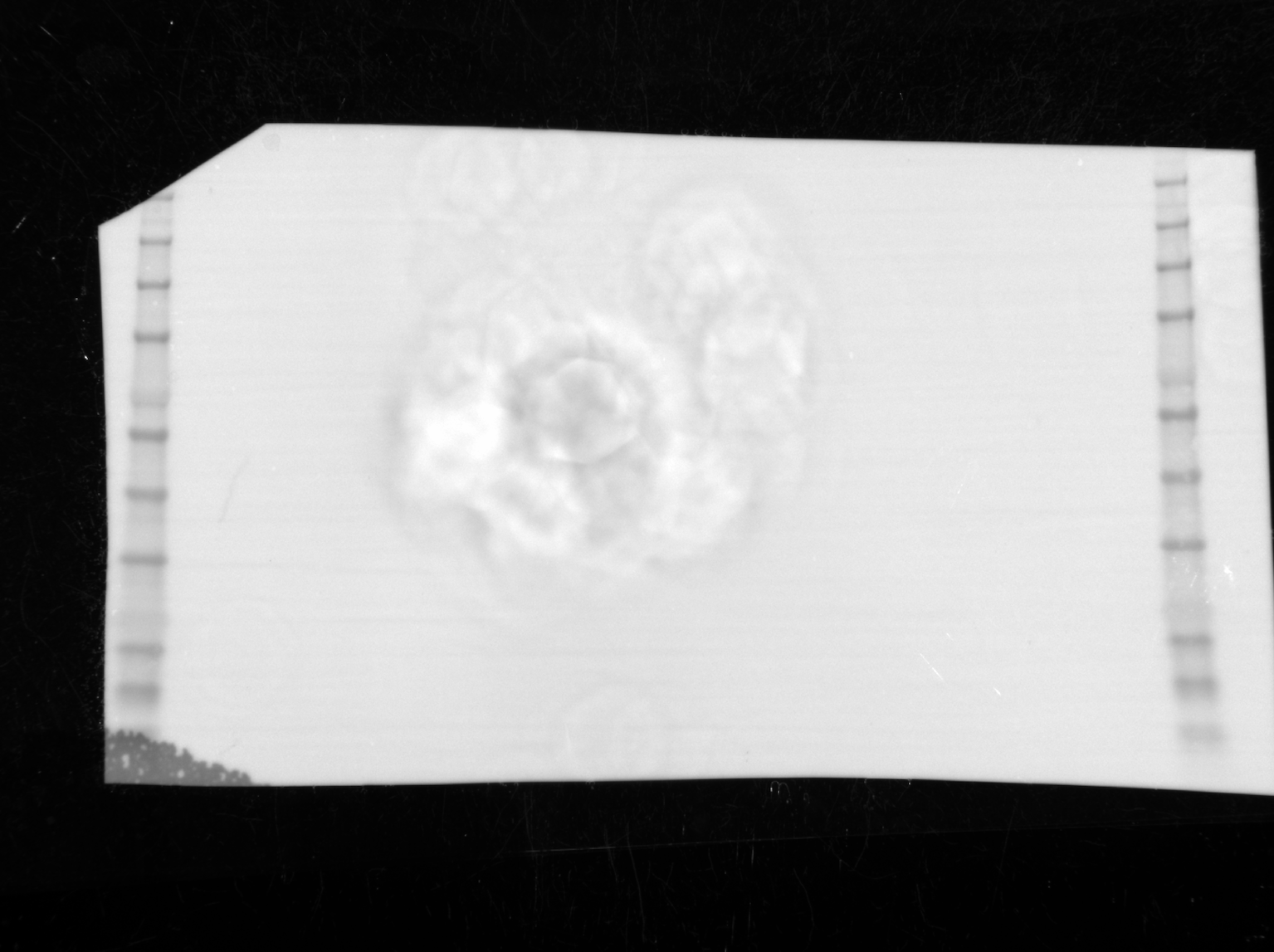

Supplement: Supplementary file 8 — Source data Fig. 1 [file 44320_2024_47_MOESM8_ESM.zip › Source Data for Figure 1/1E/LPS/tubulin gel 1/Image 2021-04-30 10hr 15min colorimetric.tif]

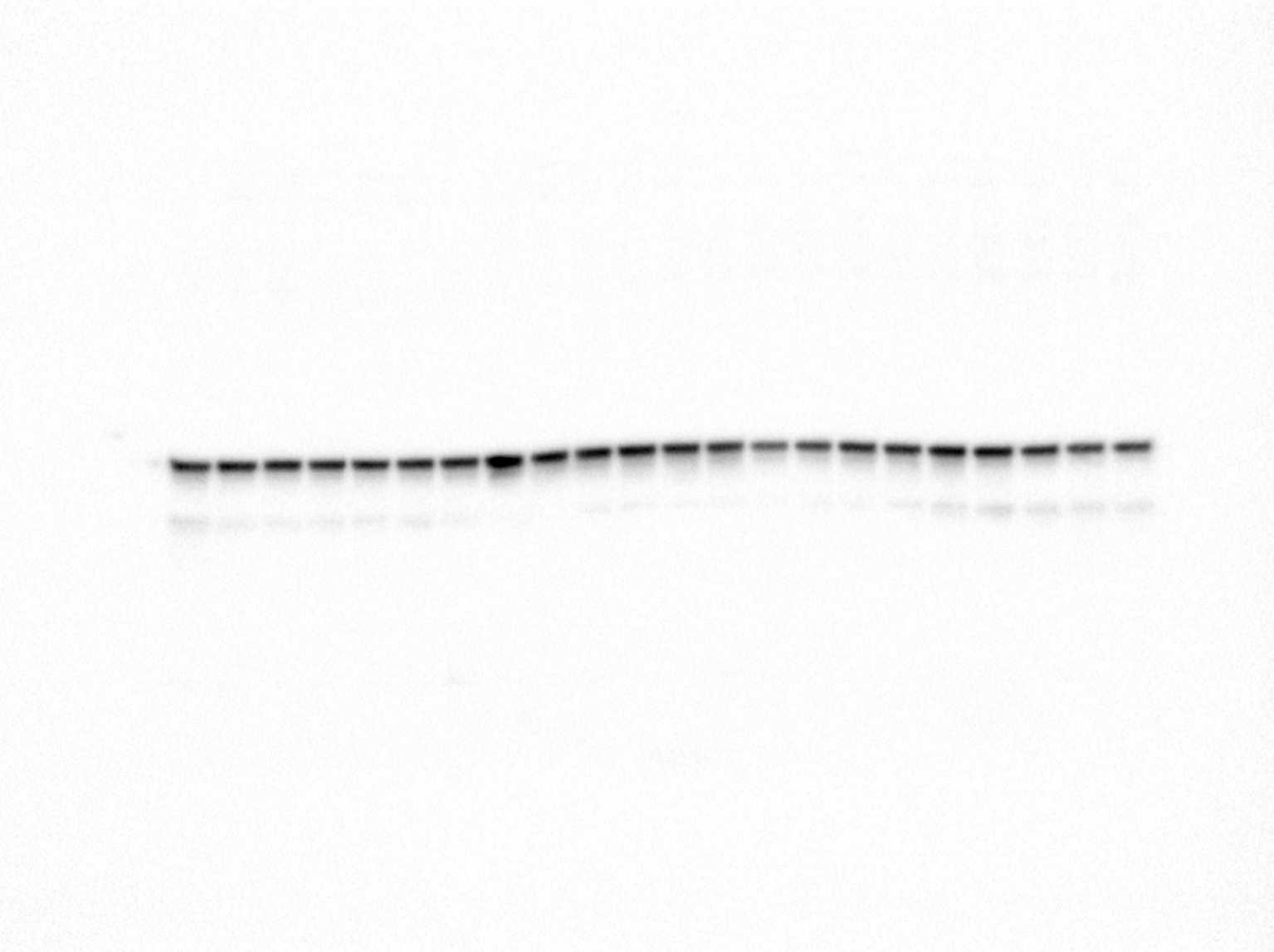

Supplement: Supplementary file 8 — Source data Fig. 1 [file 44320_2024_47_MOESM8_ESM.zip › Source Data for Figure 1/1E/LPS/tubulin gel 1/Image 2021-04-30 10hr 12min_Exposure_10.0sec.tif]

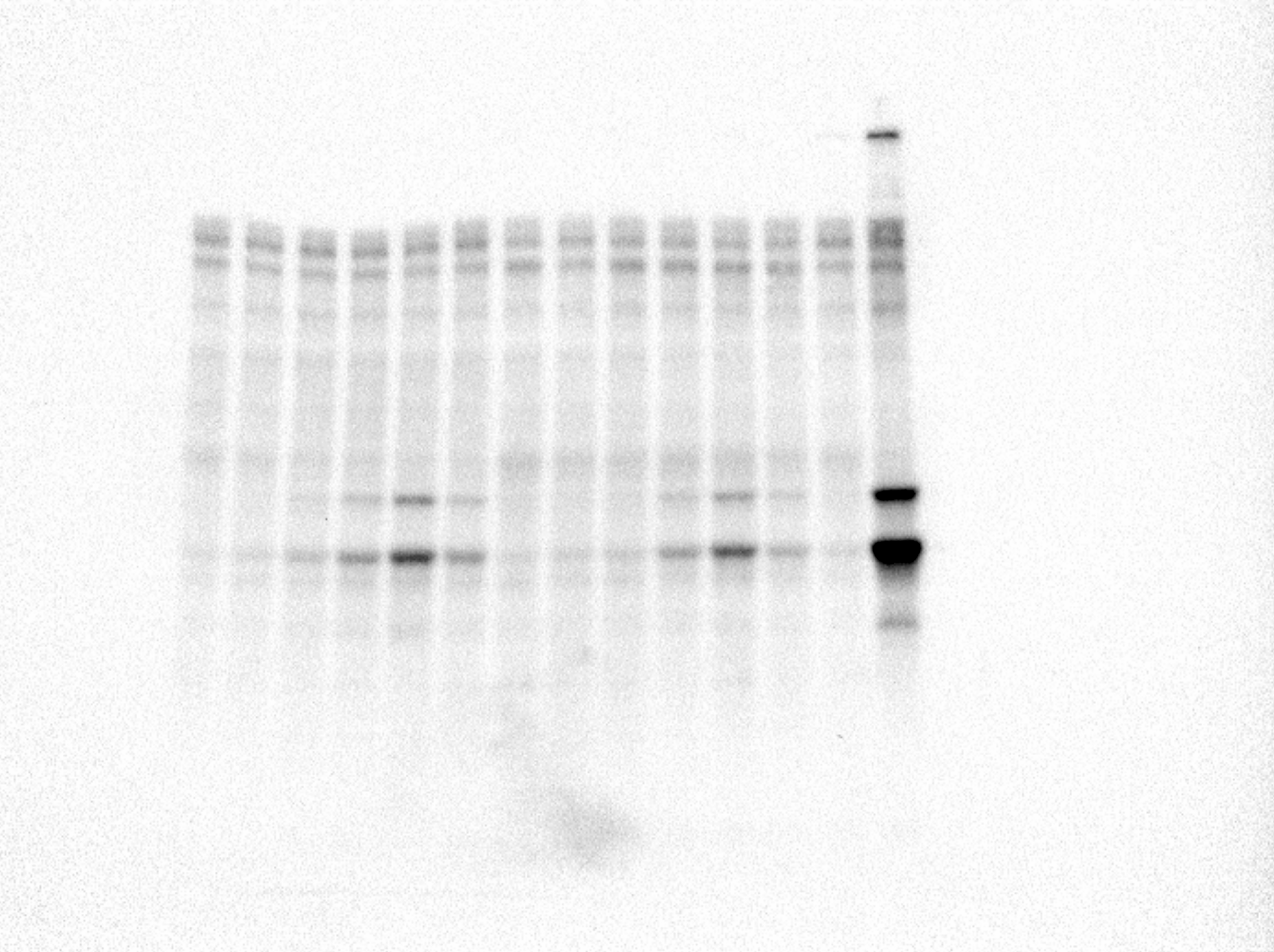

Supplement: Supplementary file 8 — Source data Fig. 1 [file 44320_2024_47_MOESM8_ESM.zip › Source Data for Figure 1/1B/1B WesternBlotImages/pMK2/Gel1/Image 2021-07-21 12hr 56min_Exposure_55.0sec.tif]

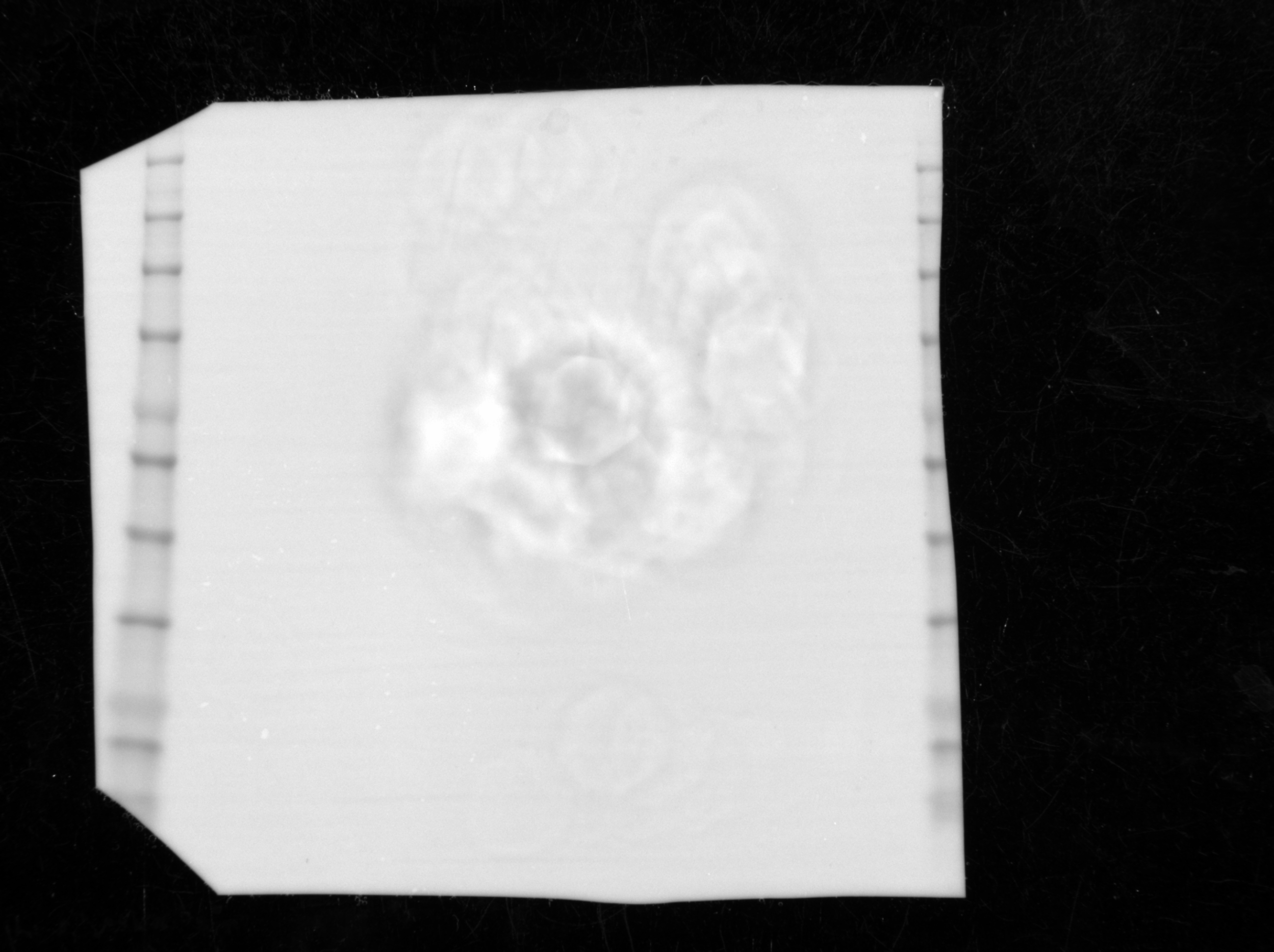

Supplement: Supplementary file 8 — Source data Fig. 1 [file 44320_2024_47_MOESM8_ESM.zip › Source Data for Figure 1/1B/1B WesternBlotImages/pMK2/Gel1/Image 2021-07-21 12hr 53min colorimetricf.tif]

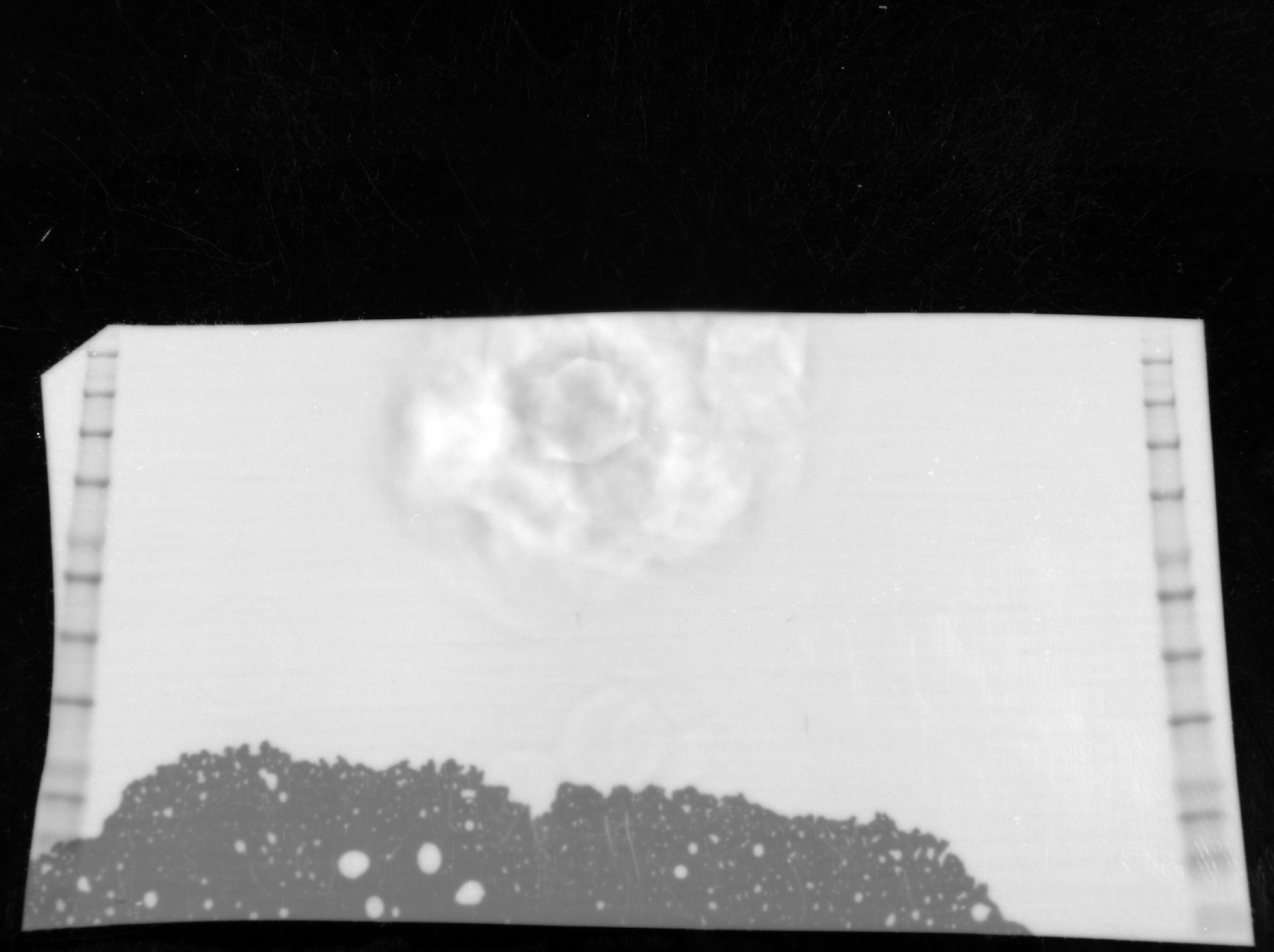

Supplement: Supplementary file 8 — Source data Fig. 1 [file 44320_2024_47_MOESM8_ESM.zip › Source Data for Figure 1/1B/1B WesternBlotImages/pMK2/Gel2/Image 2021-07-21 13hr 09min colorimetric.tif]

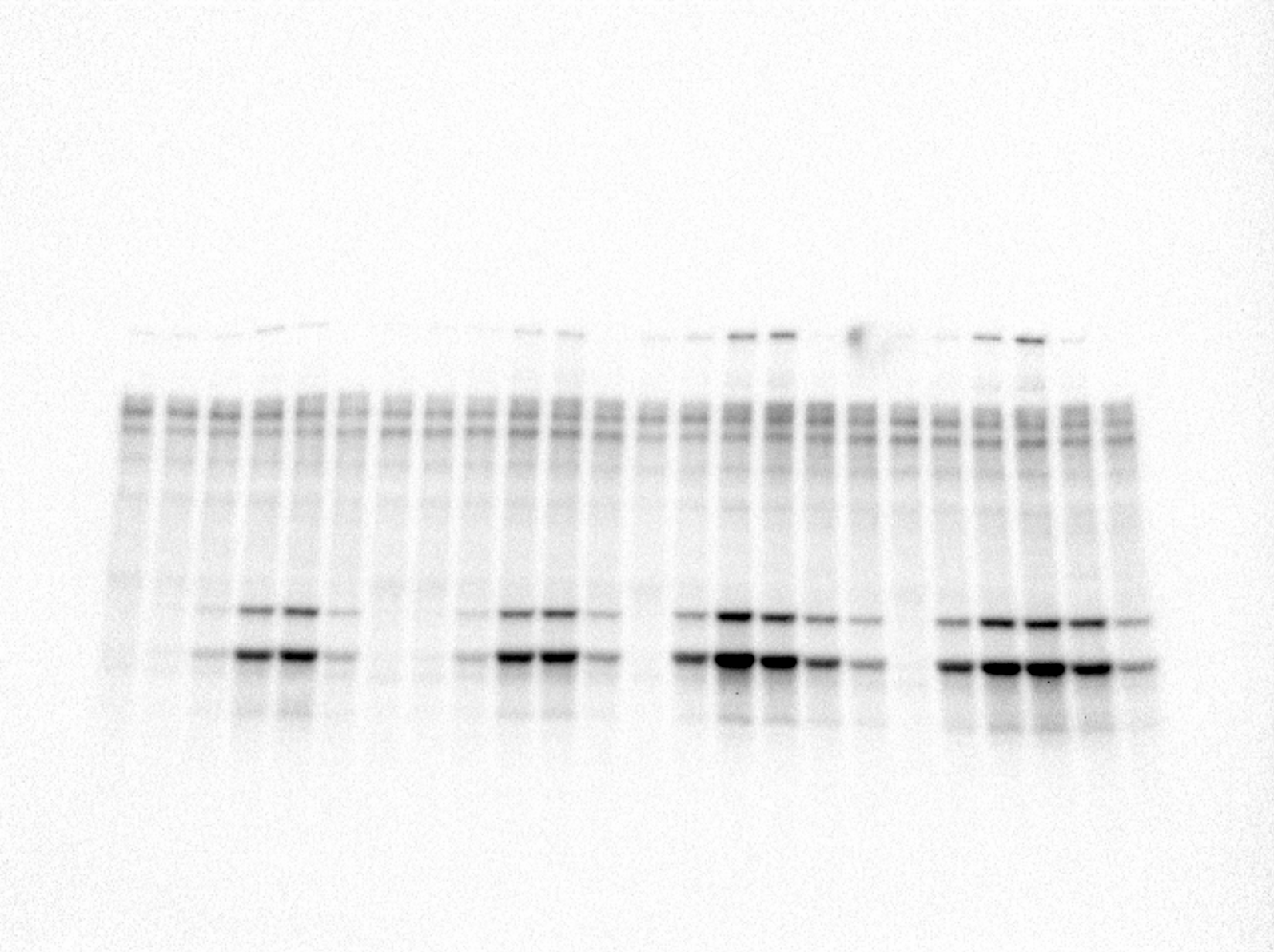

Supplement: Supplementary file 8 — Source data Fig. 1 [file 44320_2024_47_MOESM8_ESM.zip › Source Data for Figure 1/1B/1B WesternBlotImages/pMK2/Gel2/Image 2021-07-21 13hr 05min_Exposure_55.0sec.tif]

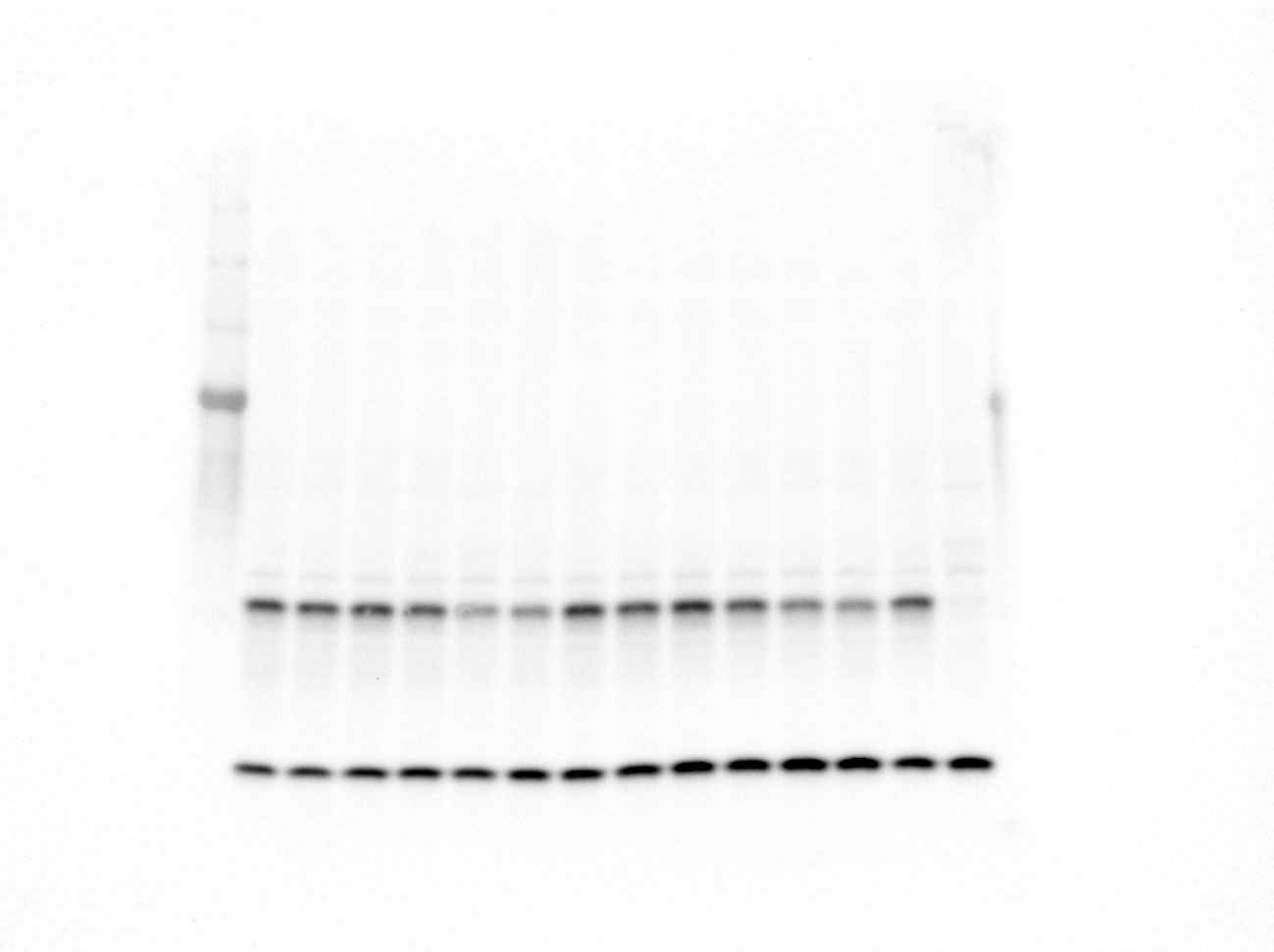

Supplement: Supplementary file 8 — Source data Fig. 1 [file 44320_2024_47_MOESM8_ESM.zip › Source Data for Figure 1/1B/1B WesternBlotImages/IkBa/Gel1/Image 2021-07-23 11hr 25min_Exposure_60.0sec.tif]

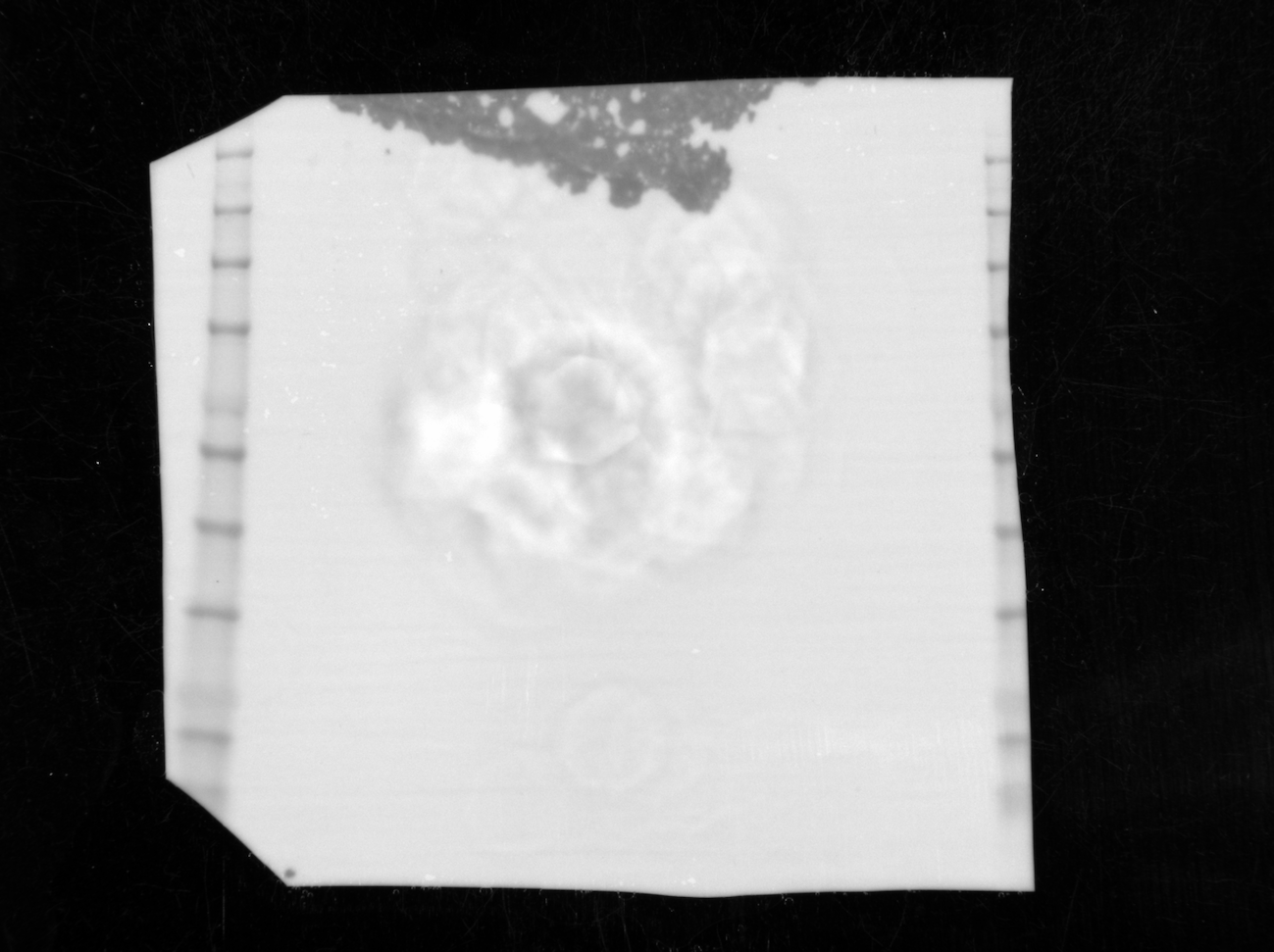

Supplement: Supplementary file 8 — Source data Fig. 1 [file 44320_2024_47_MOESM8_ESM.zip › Source Data for Figure 1/1B/1B WesternBlotImages/IkBa/Gel1/Image 2021-07-23 11hr 24min colorimteric.tif]

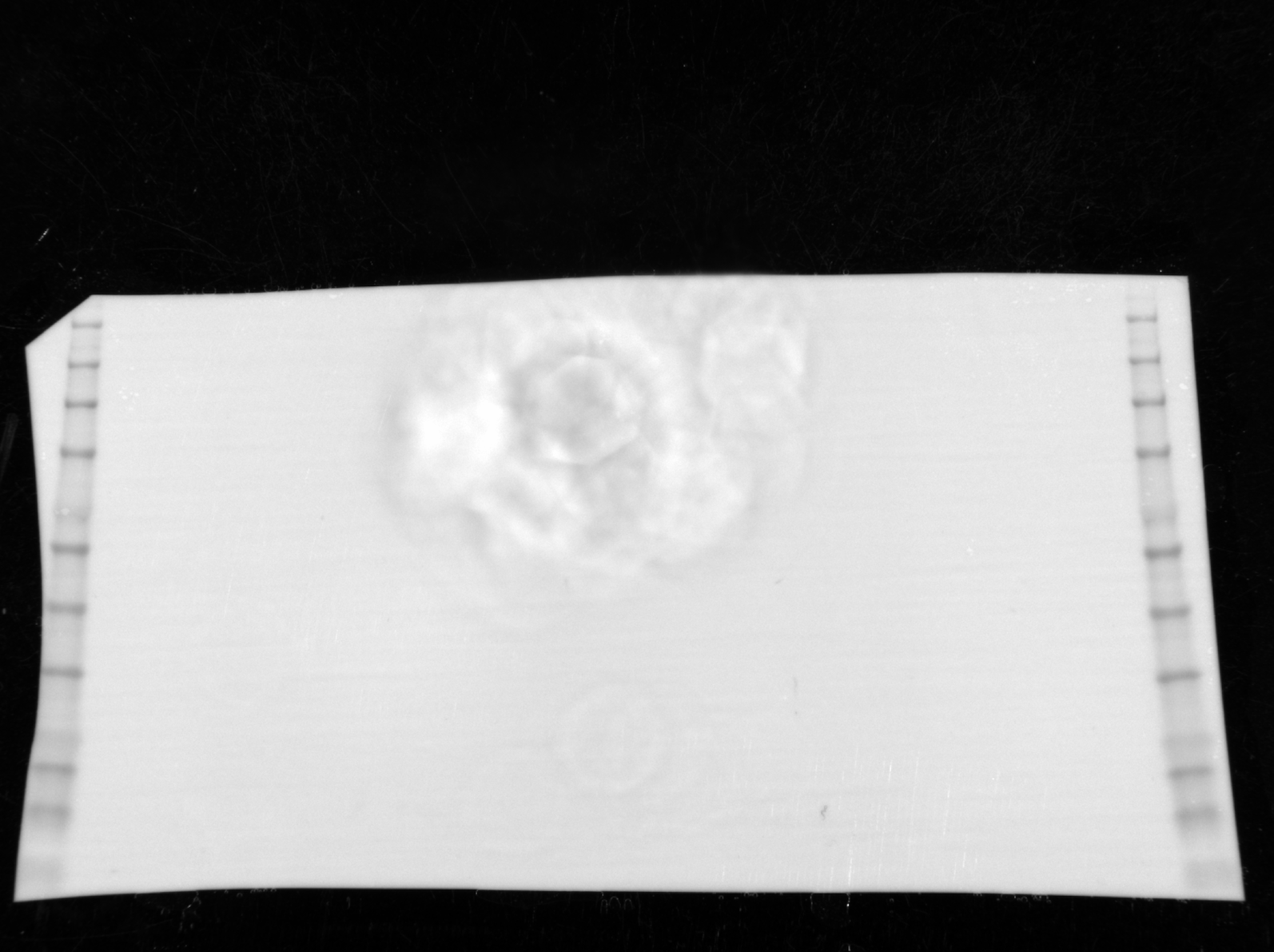

Supplement: Supplementary file 8 — Source data Fig. 1 [file 44320_2024_47_MOESM8_ESM.zip › Source Data for Figure 1/1B/1B WesternBlotImages/IkBa/Gel2/Image 2021-07-23 11hr 39min colorimetric.tif]

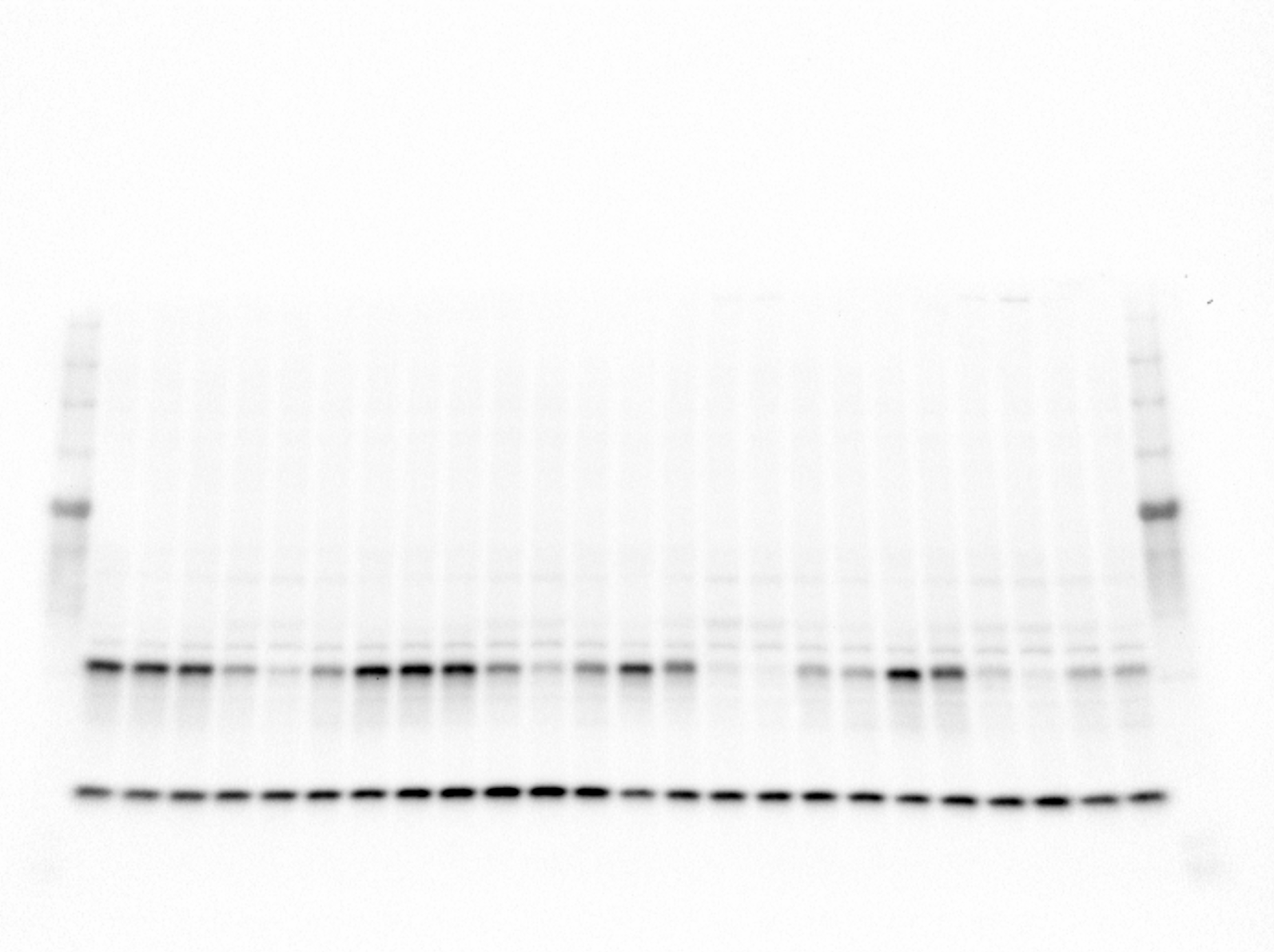

Supplement: Supplementary file 8 — Source data Fig. 1 [file 44320_2024_47_MOESM8_ESM.zip › Source Data for Figure 1/1B/1B WesternBlotImages/IkBa/Gel2/Image 2021-07-23 11hr 33min_Exposure_50.0sec.tif]

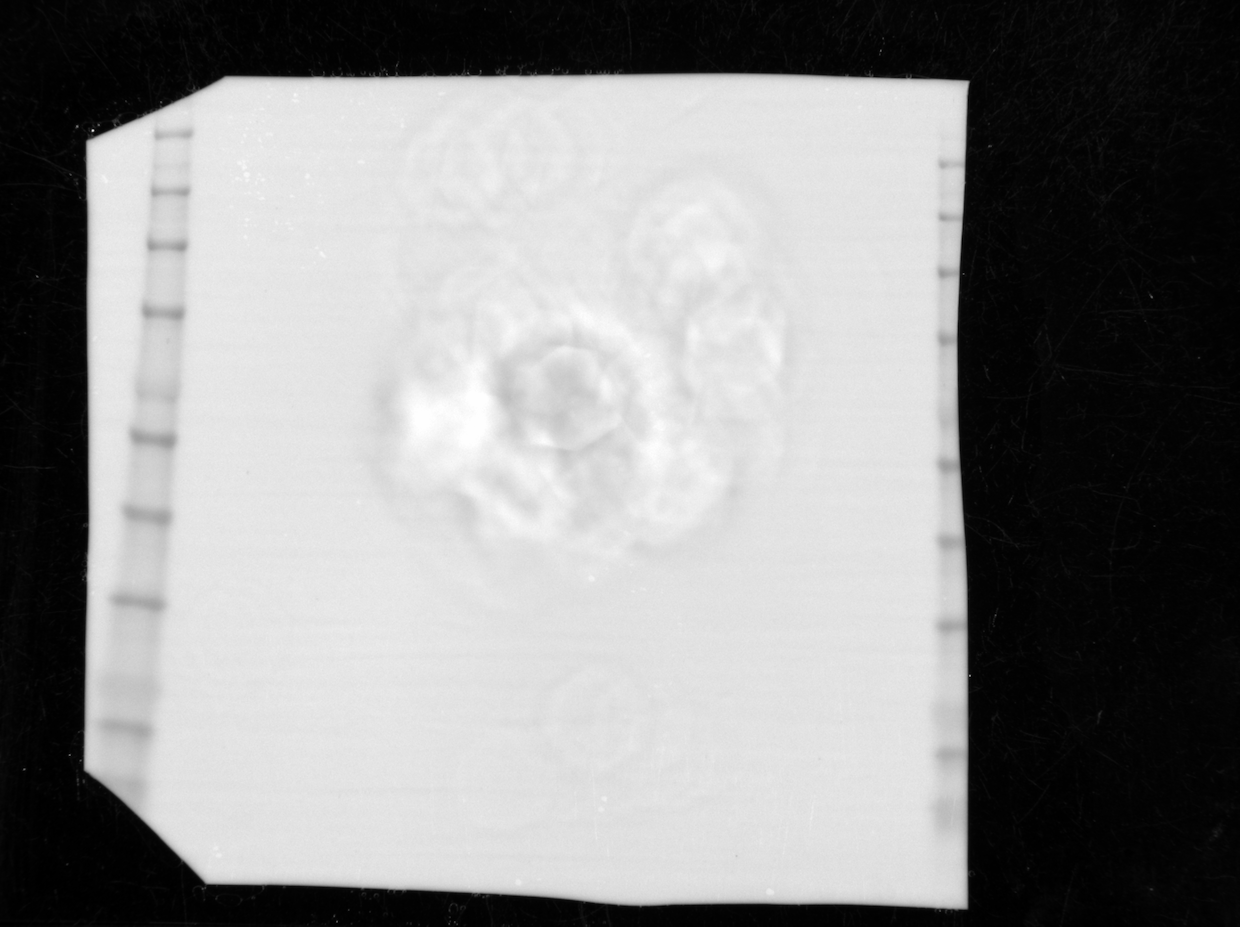

Supplement: Supplementary file 8 — Source data Fig. 1 [file 44320_2024_47_MOESM8_ESM.zip › Source Data for Figure 1/1B/1B WesternBlotImages/tubulin A/Gel1/Image 2021-07-24 15hr 59min colorimetric.tif]

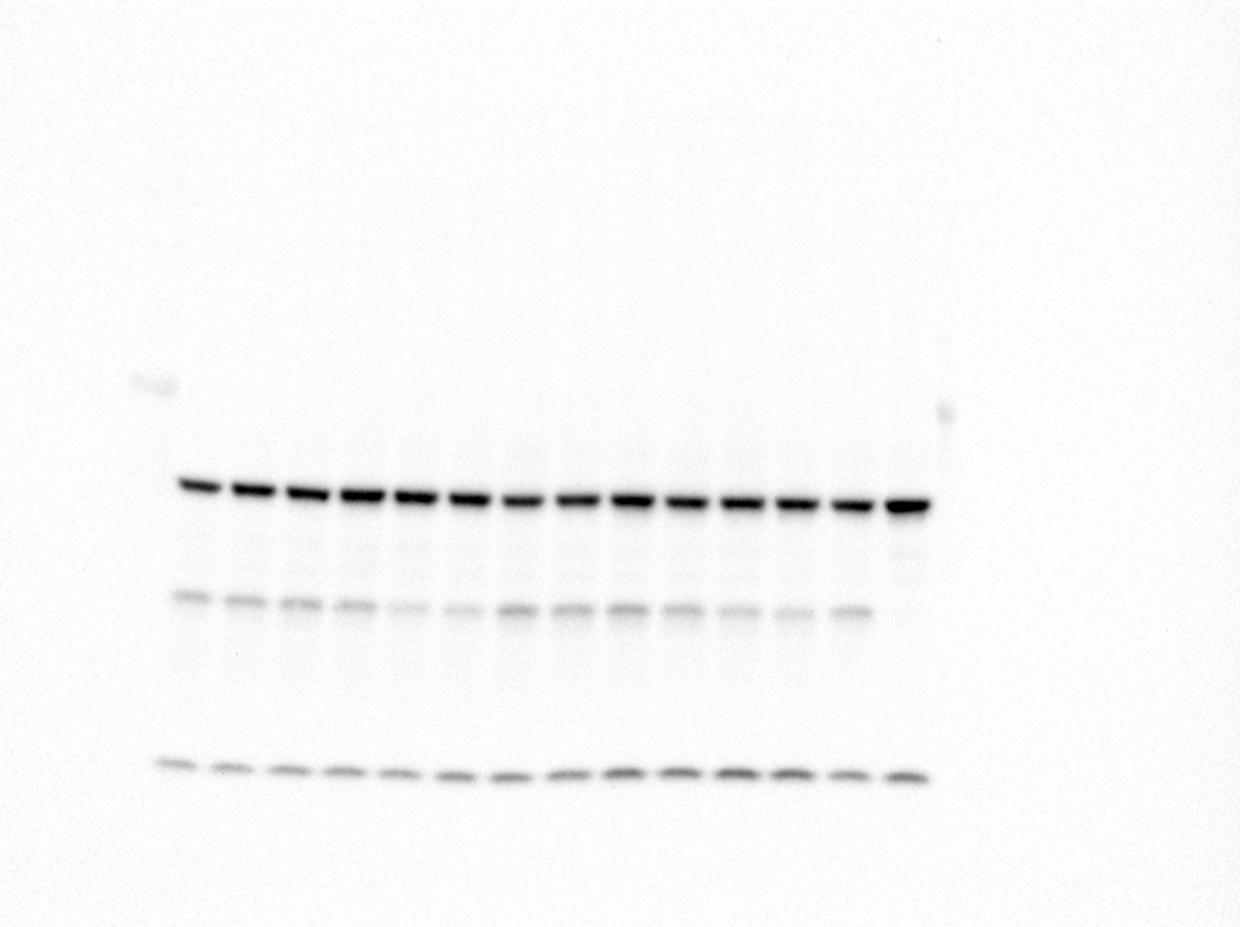

Supplement: Supplementary file 8 — Source data Fig. 1 [file 44320_2024_47_MOESM8_ESM.zip › Source Data for Figure 1/1B/1B WesternBlotImages/tubulin A/Gel1/Image 2021-07-24 16hr 00min_Exposure_60.0sec.tif]

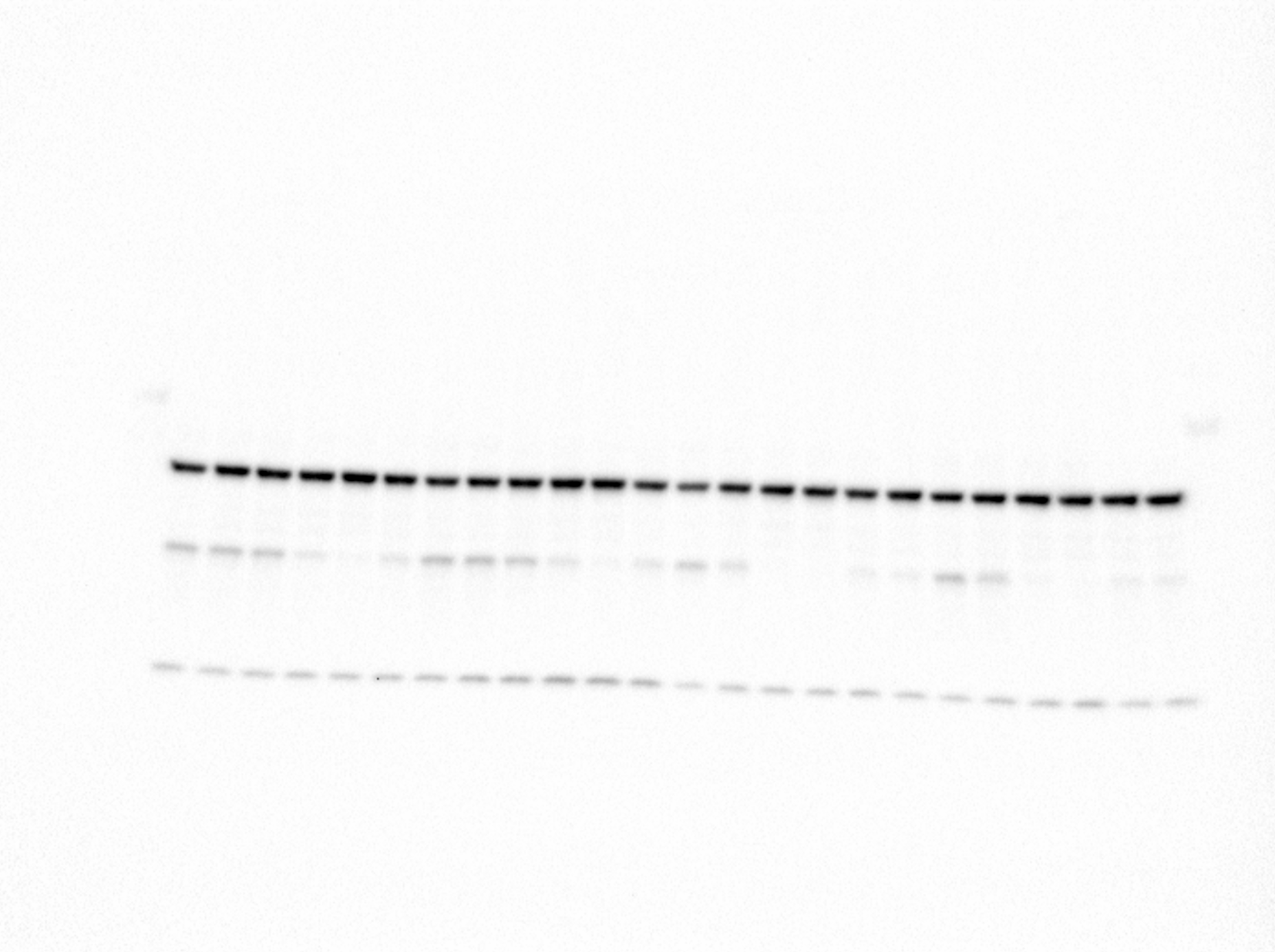

Supplement: Supplementary file 8 — Source data Fig. 1 [file 44320_2024_47_MOESM8_ESM.zip › Source Data for Figure 1/1B/1B WesternBlotImages/tubulin A/Gel2/Image 2021-07-24 16hr 05min_Exposure_80.0sec.tif]

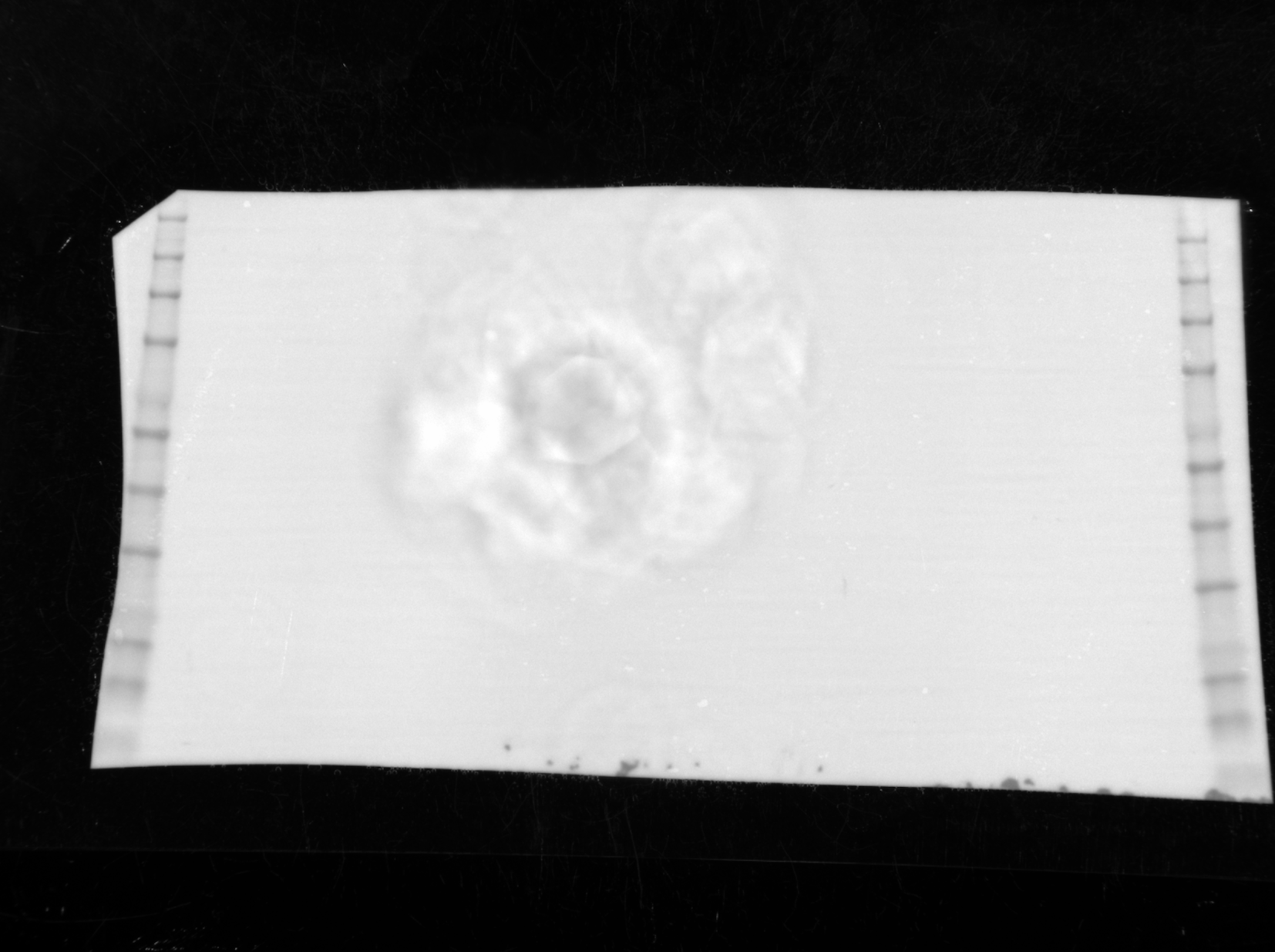

Supplement: Supplementary file 8 — Source data Fig. 1 [file 44320_2024_47_MOESM8_ESM.zip › Source Data for Figure 1/1B/1B WesternBlotImages/tubulin A/Gel2/Image 2021-07-24 16hr 08min coorimtreic.tif]

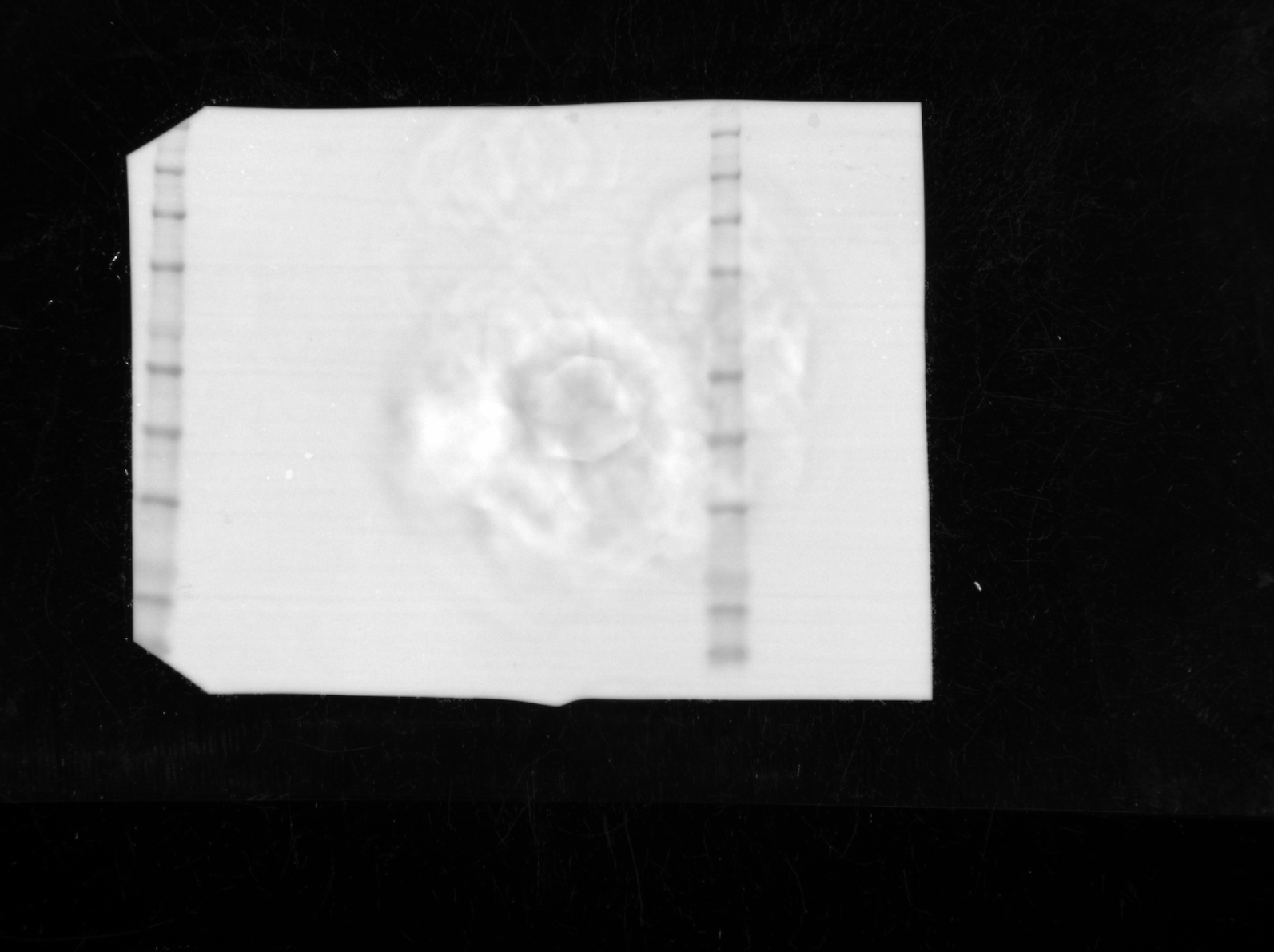

Supplement: Supplementary file 8 — Source data Fig. 1 [file 44320_2024_47_MOESM8_ESM.zip › Source Data for Figure 1/1B/1B WesternBlotImages/p-p38/Gel1/Image 2021-07-22 11hr 12min coloriemtric.tif]

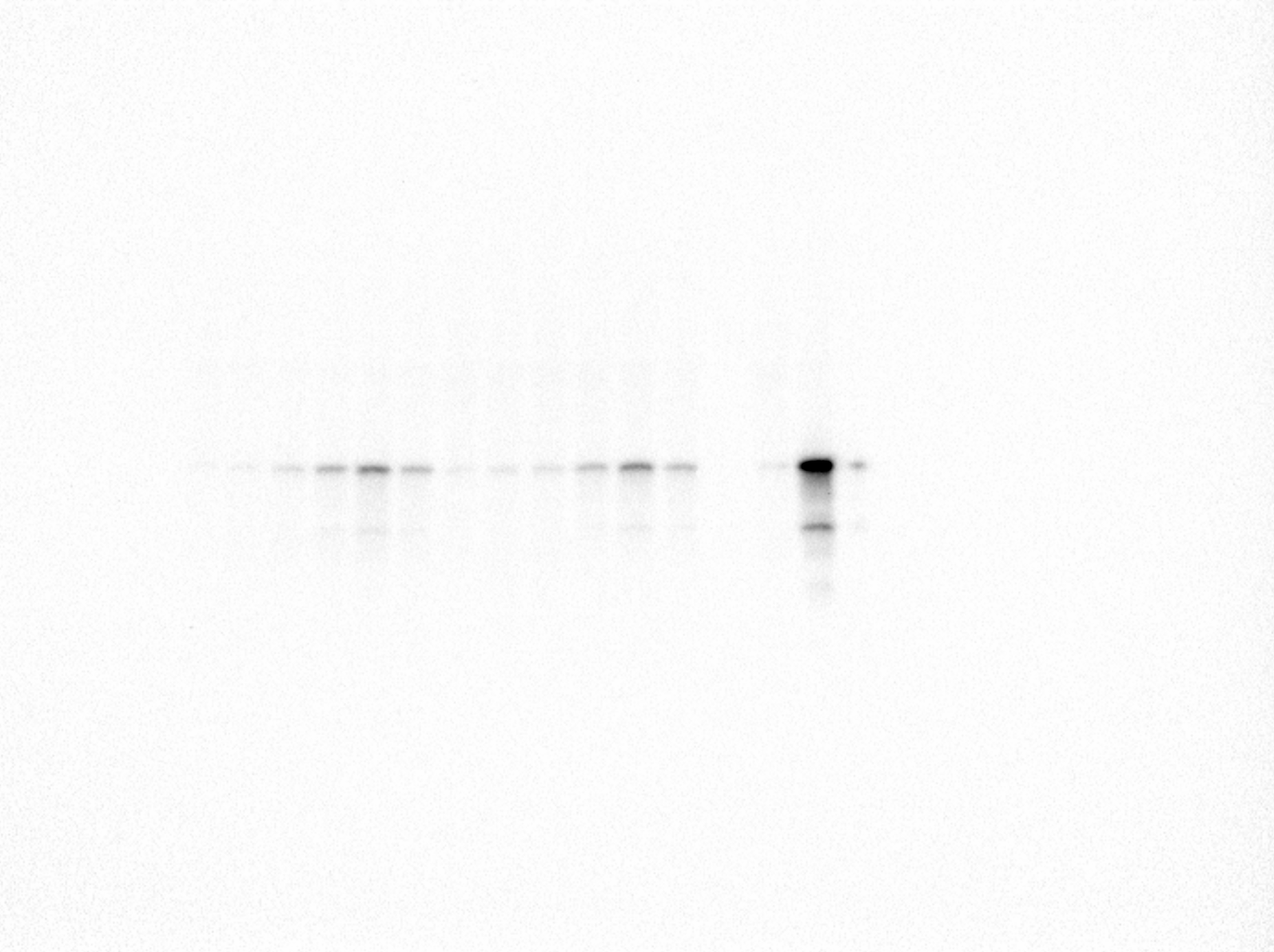

Supplement: Supplementary file 8 — Source data Fig. 1 [file 44320_2024_47_MOESM8_ESM.zip › Source Data for Figure 1/1B/1B WesternBlotImages/p-p38/Gel1/Image 2021-07-22 11hr 13min_Exposure_35.0sec adjusted.tif]

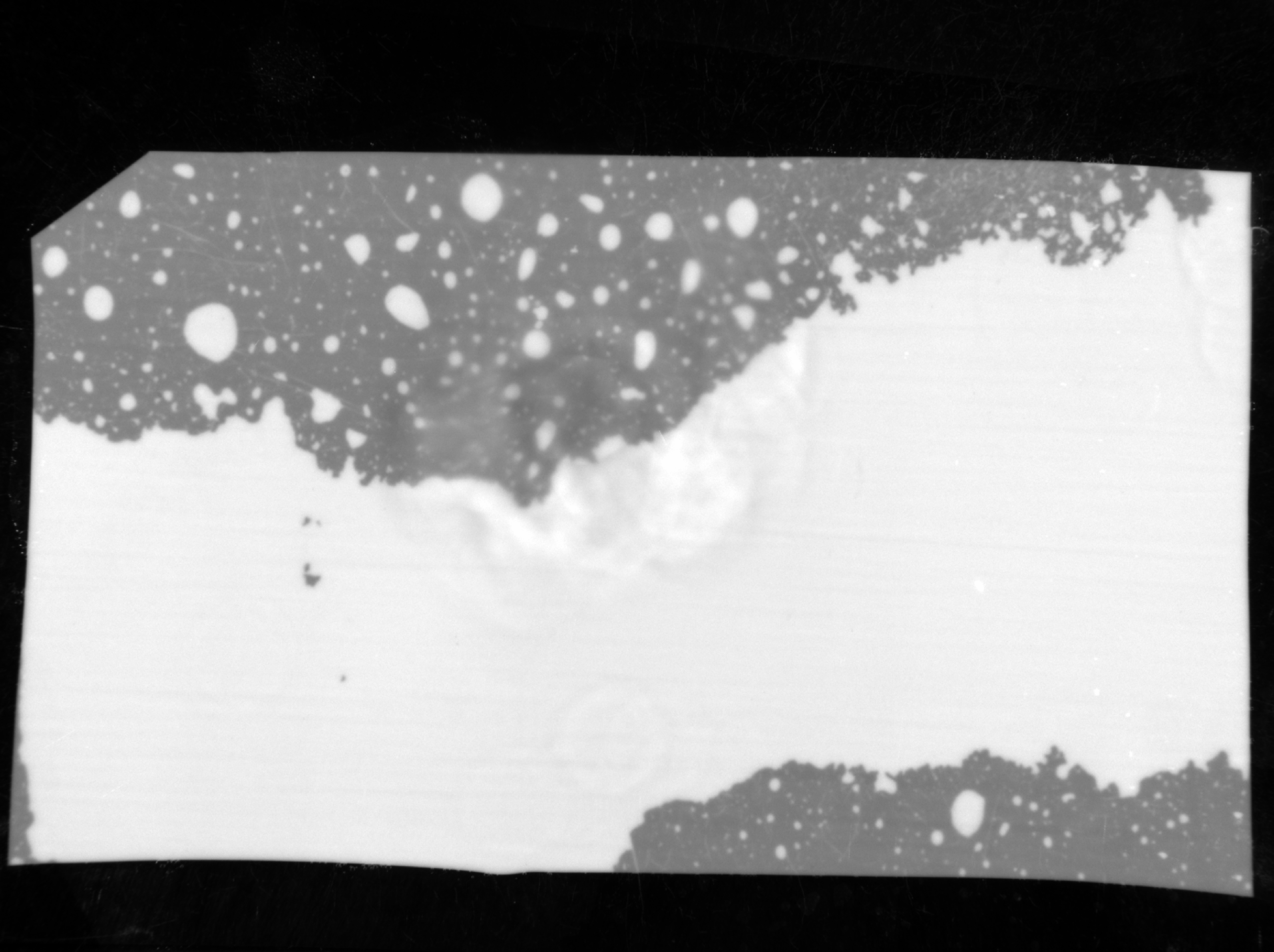

Supplement: Supplementary file 8 — Source data Fig. 1 [file 44320_2024_47_MOESM8_ESM.zip › Source Data for Figure 1/1B/1B WesternBlotImages/p-p38/Gel2/Image 2021-07-22 11hr 30min colorimtreic.tif]

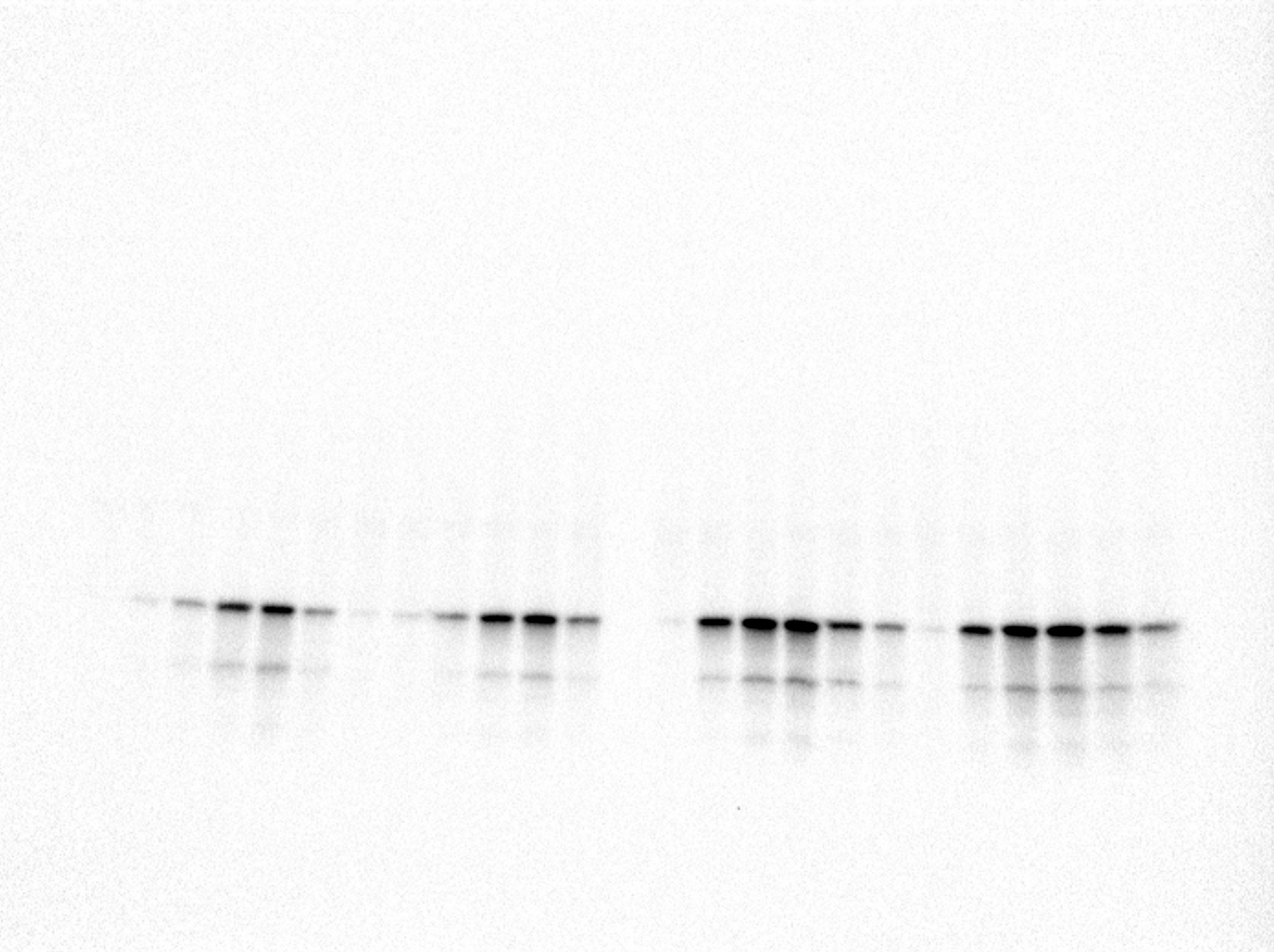

Supplement: Supplementary file 8 — Source data Fig. 1 [file 44320_2024_47_MOESM8_ESM.zip › Source Data for Figure 1/1B/1B WesternBlotImages/p-p38/Gel2/Image 2021-07-22 11hr 22min_Exposure_60.0sec.tif]

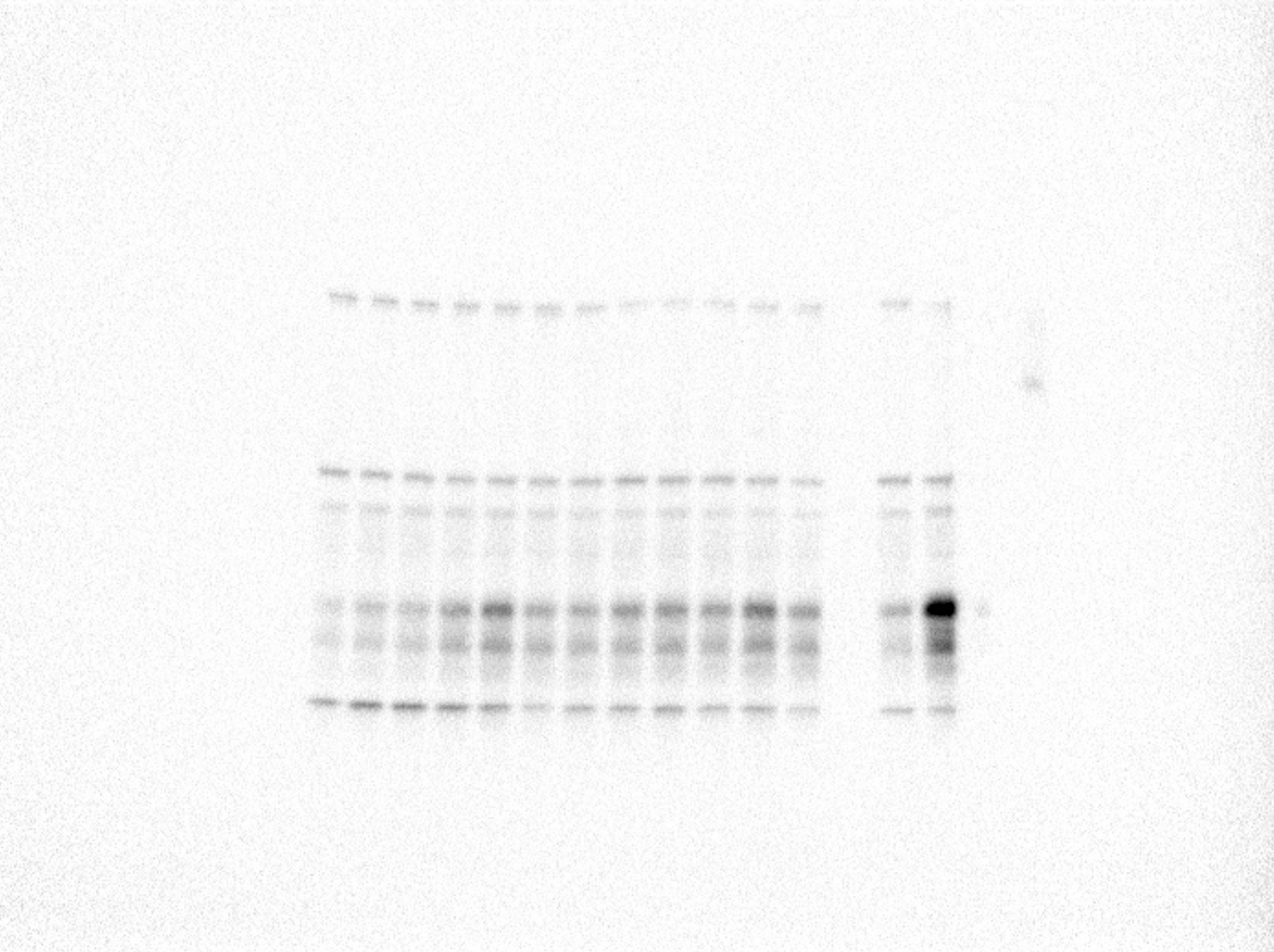

Supplement: Supplementary file 8 — Source data Fig. 1 [file 44320_2024_47_MOESM8_ESM.zip › Source Data for Figure 1/1B/1B WesternBlotImages/pCREB/Gel1/Image 2021-07-21 13hr 17min_Exposure_60.0sec.tif]

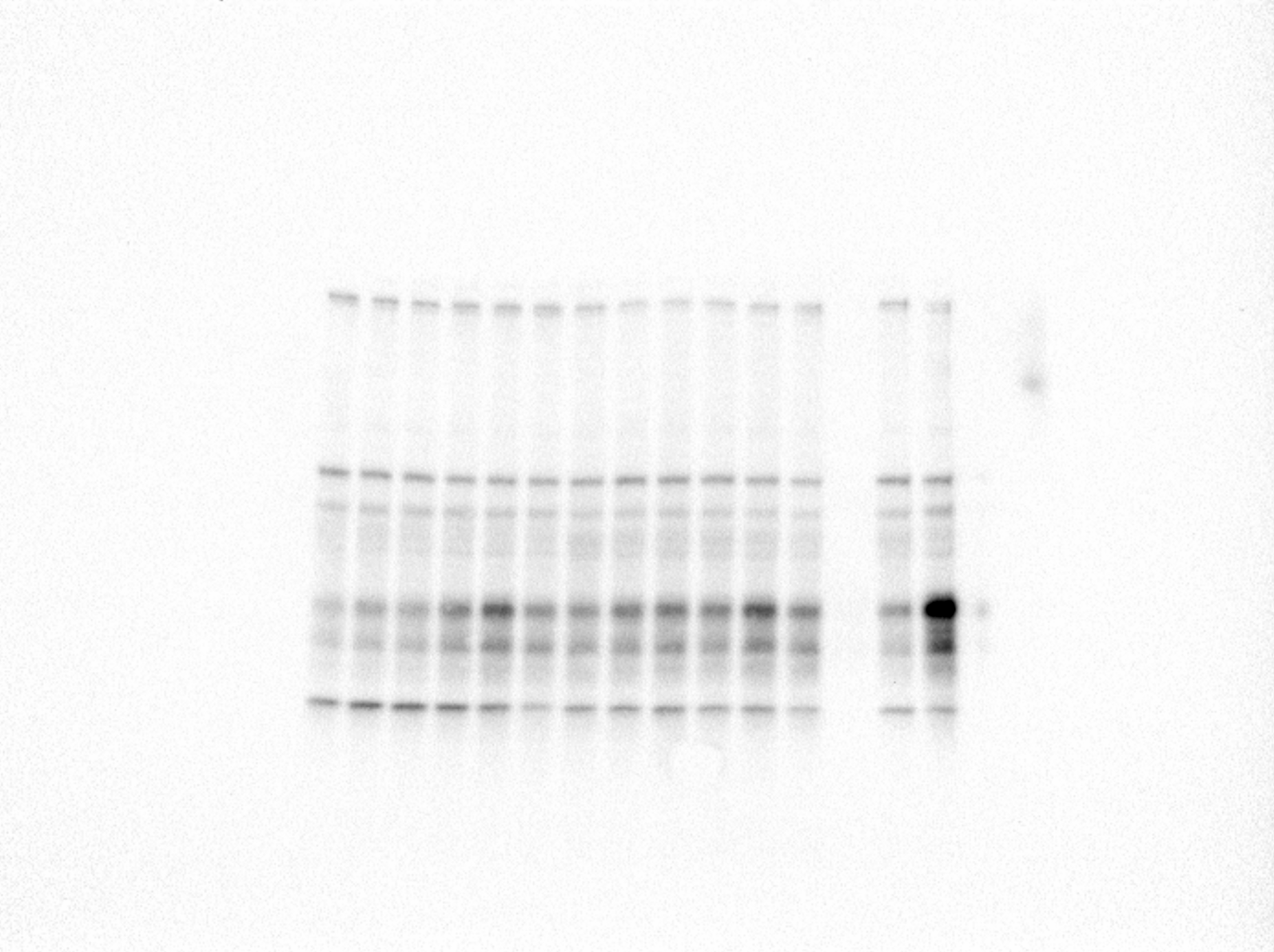

Supplement: Supplementary file 8 — Source data Fig. 1 [file 44320_2024_47_MOESM8_ESM.zip › Source Data for Figure 1/1B/1B WesternBlotImages/pCREB/Gel1/Image 2021-07-21 13hr 22min_Exposure_120.0sec.tif]

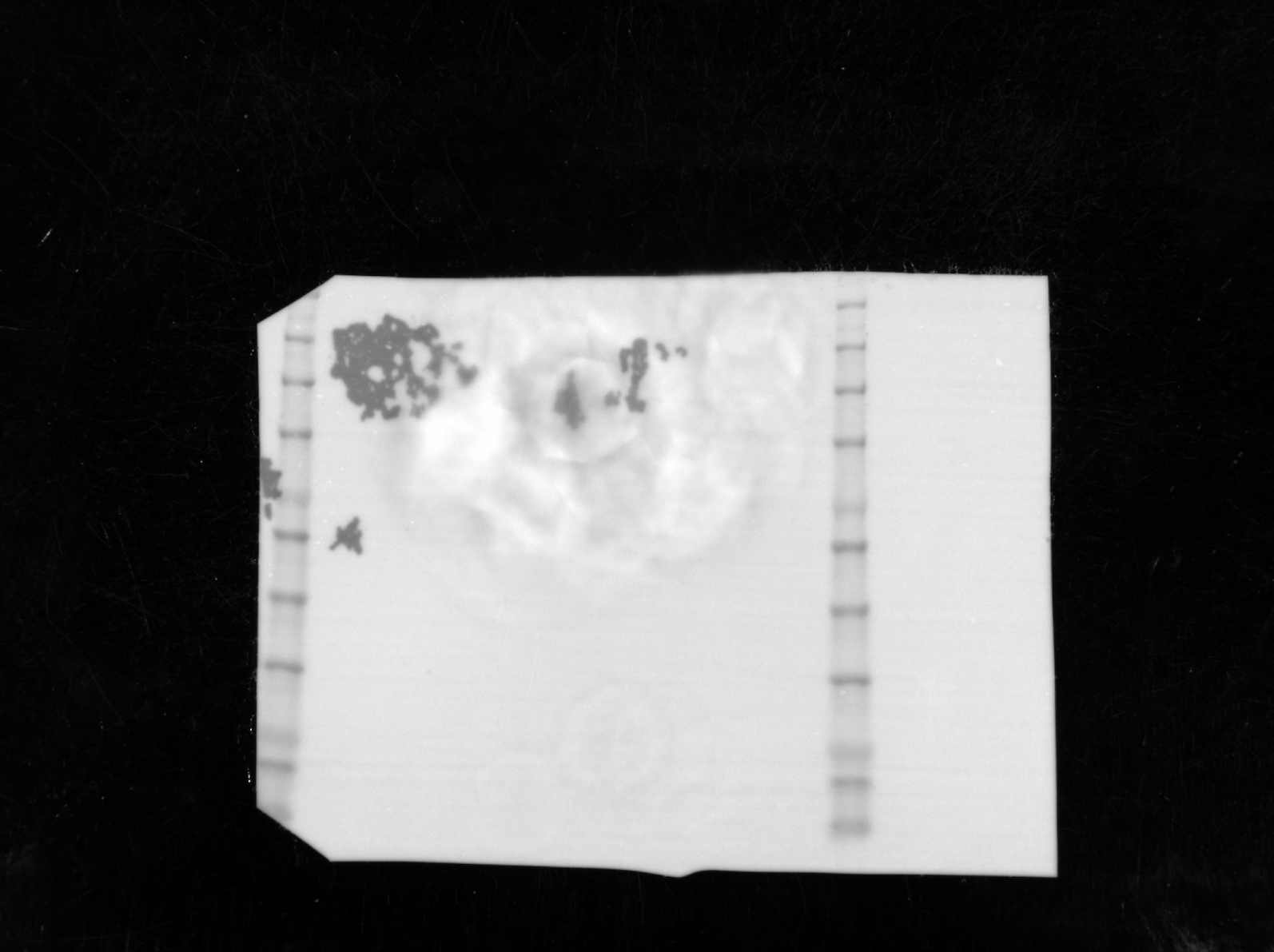

Supplement: Supplementary file 8 — Source data Fig. 1 [file 44320_2024_47_MOESM8_ESM.zip › Source Data for Figure 1/1B/1B WesternBlotImages/pCREB/Gel1/Image 2021-07-21 13hr 16min colorimetric.tif]

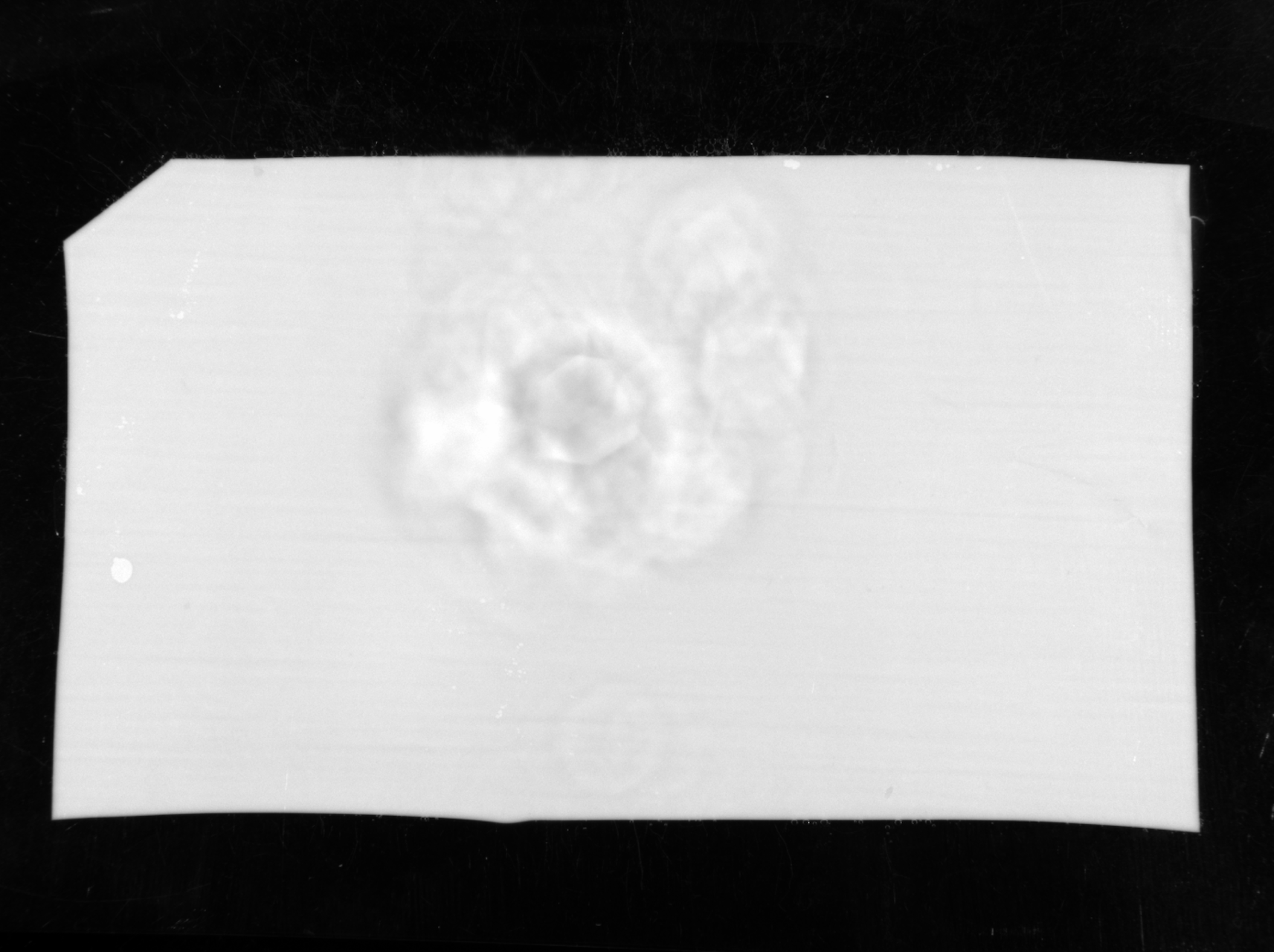

Supplement: Supplementary file 8 — Source data Fig. 1 [file 44320_2024_47_MOESM8_ESM.zip › Source Data for Figure 1/1B/1B WesternBlotImages/pCREB/Gel2/Image 2021-07-21 13hr 30min colorimetric.tif]

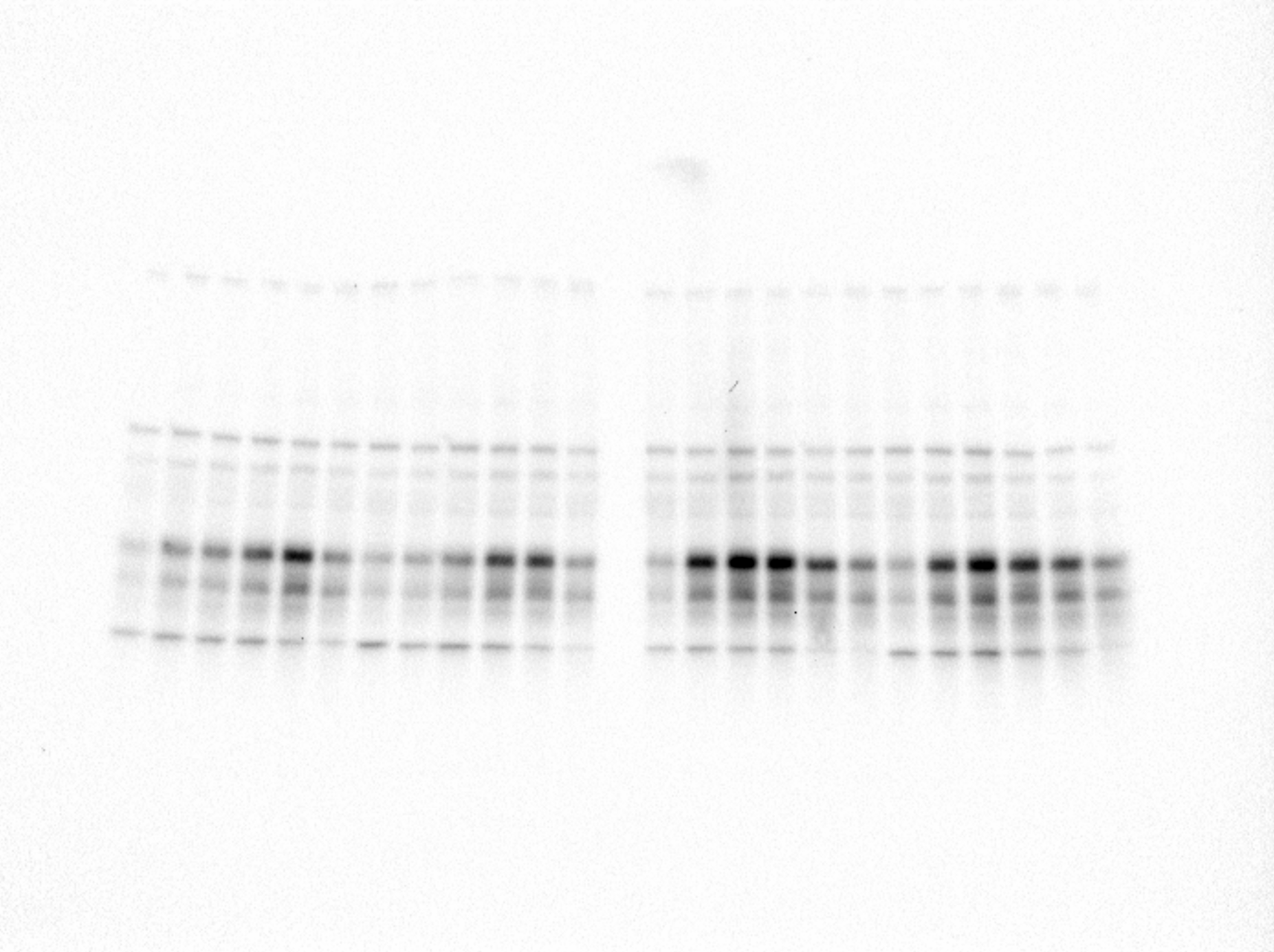

Supplement: Supplementary file 8 — Source data Fig. 1 [file 44320_2024_47_MOESM8_ESM.zip › Source Data for Figure 1/1B/1B WesternBlotImages/pCREB/Gel2/Image 2021-07-21 13hr 26min_Exposure_70.0sec.tif]

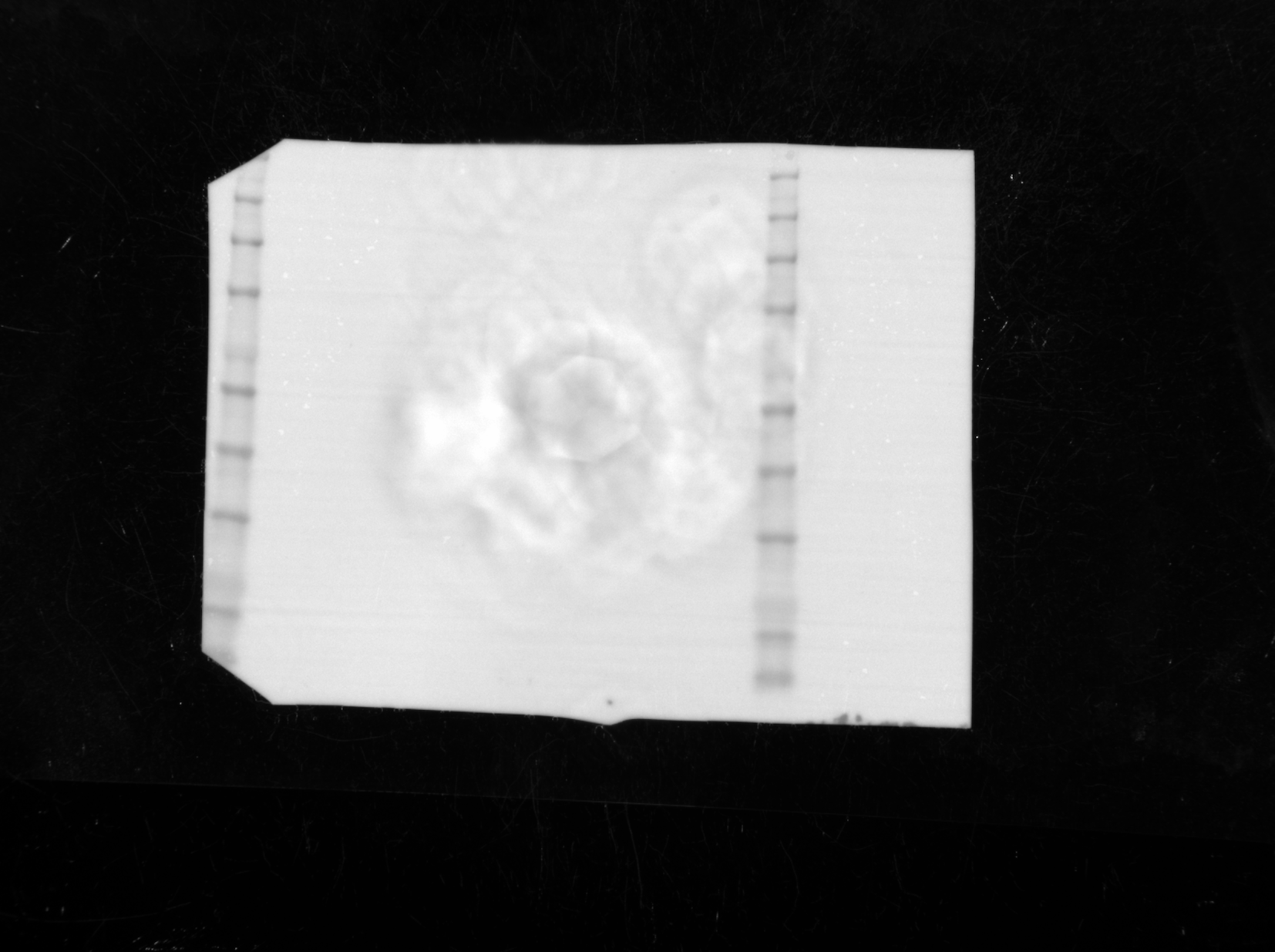

Supplement: Supplementary file 8 — Source data Fig. 1 [file 44320_2024_47_MOESM8_ESM.zip › Source Data for Figure 1/1B/1B WesternBlotImages/tubulin B/Gel1/Image 2021-07-24 16hr 12min colorimetric.tif]

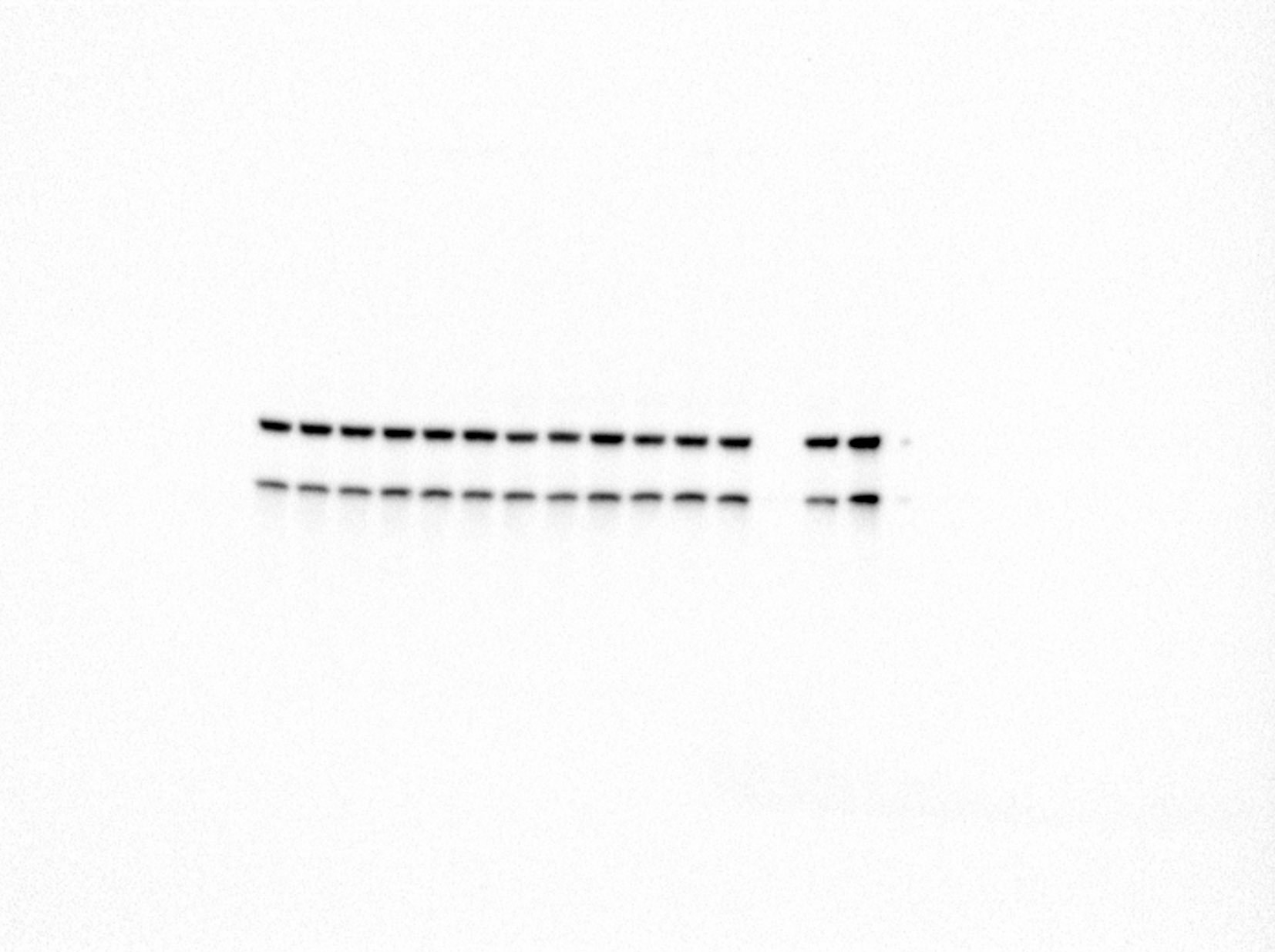

Supplement: Supplementary file 8 — Source data Fig. 1 [file 44320_2024_47_MOESM8_ESM.zip › Source Data for Figure 1/1B/1B WesternBlotImages/tubulin B/Gel1/Image 2021-07-24 16hr 13min_Exposure_90.0sec.tif]

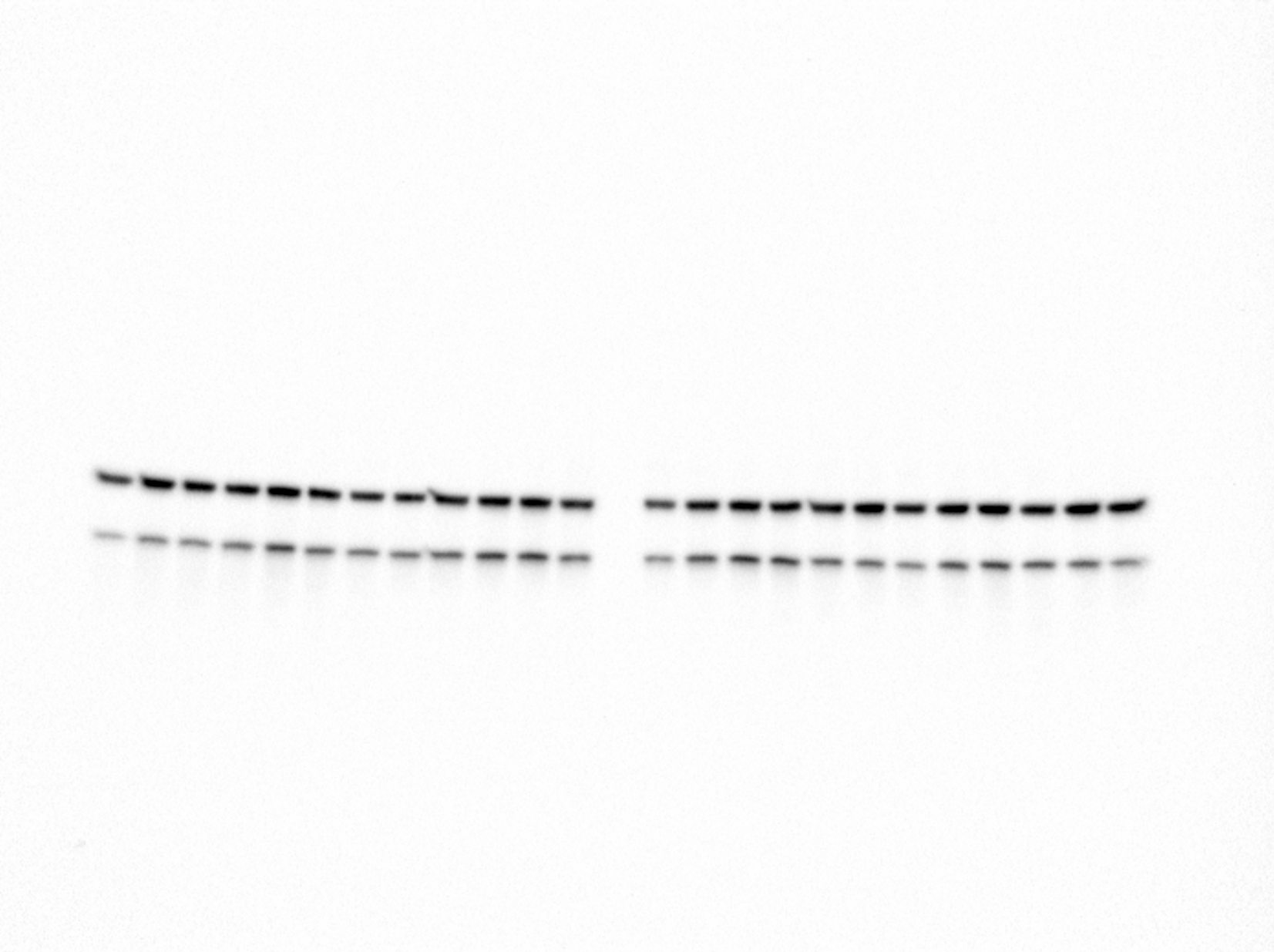

Supplement: Supplementary file 8 — Source data Fig. 1 [file 44320_2024_47_MOESM8_ESM.zip › Source Data for Figure 1/1B/1B WesternBlotImages/tubulin B/Gel2/Image 2021-07-24 16hr 17min_Exposure_70.0sec.tif]

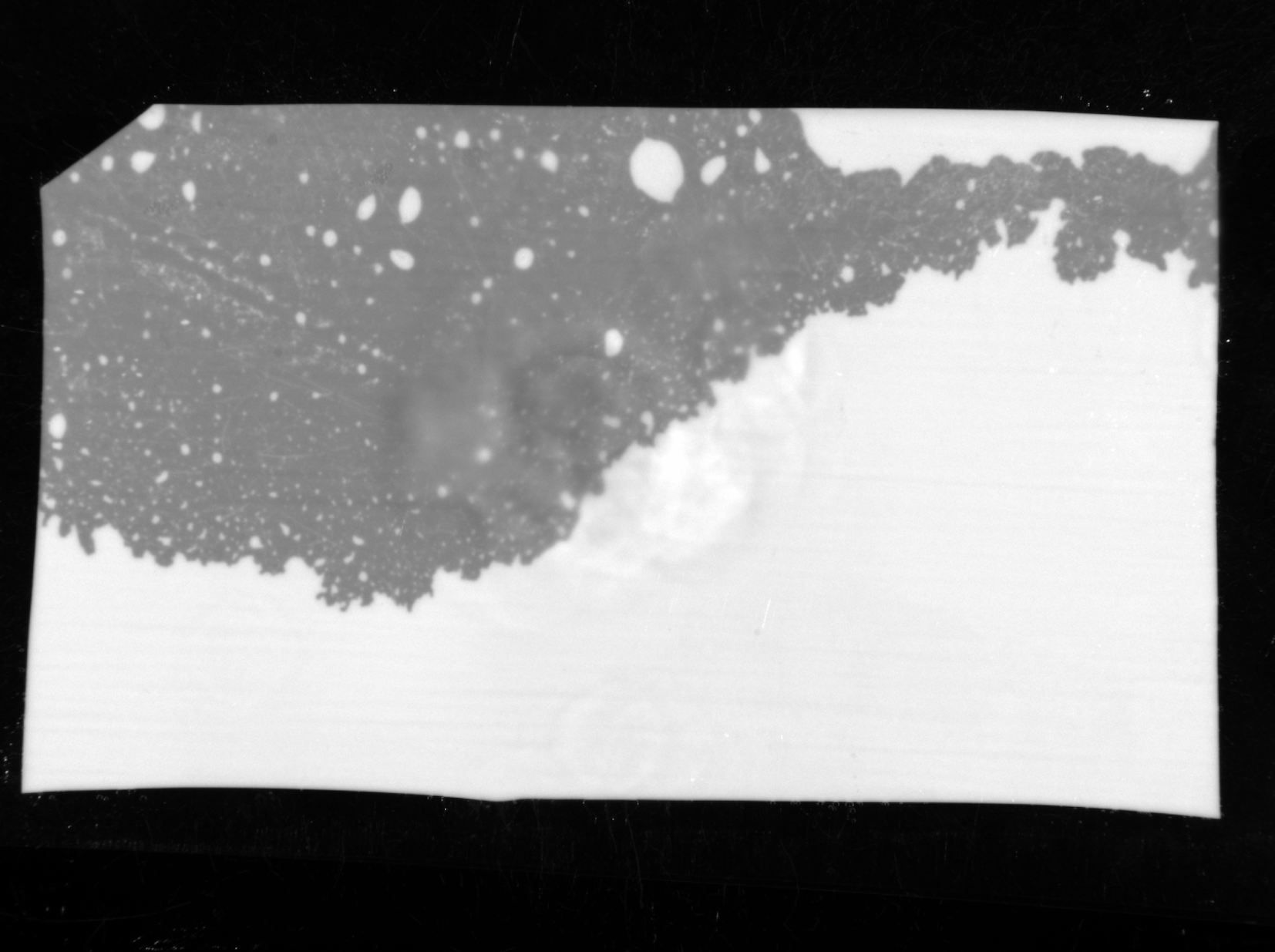

Supplement: Supplementary file 8 — Source data Fig. 1 [file 44320_2024_47_MOESM8_ESM.zip › Source Data for Figure 1/1B/1B WesternBlotImages/tubulin B/Gel2/Image 2021-07-24 16hr 21min colorimetric.tif]
