# Supplementary material for: Dynamical and combinatorial coding by MAPK p38 and NFκB in the inflammatory response of macrophages
Source: Mol Syst Biol. 2024 Jun 13;20(8):898–932. doi: 10.1038/s44320-024-00047-4 (PMC11297158; doi:10.1038/s44320-024-00047-4)
Supplement: Supplementary file 11 — Expanded View Figures [file 44320_2024_47_MOESM11_ESM.pdf]

Expanded View Figures

Figure EV1. Quality control of the experimental system for quantifying p38 and NFκB activity dynamics in murine macrophages.

(**A**) Gating strategy for FACS of mVenus-RelA+ p38-KTR-mCerulean+ hMPs. (**B**) mCerulean and mVenus expression by flow cytometry in WT hMPs, mVenus-Rela hMPs, and mVenus-Rela hMPs transduced with p38-KTR-mCerulean before FACS. (**C**) Signaling responses to three LPS doses over 2 h in mVenus-Rela p38-KTR-mCerulean hMPDMs compared to parent cell line by Western Blotting for phospho-p38, phospho -MK2, phospho-CREB, and IκBα protein levels. Quantification in Fig. 1B. (**D**) Bulk p-p38 and p-MK2 protein levels in hMPDMs measured by Western Blotting in response to indicated stimulations over 4 h. Quantification in Figs. 1E and EV1D. (**E**) Comparison of p38 activity over 4 h in hMPDMs measured by p38-KTR microscopy (mean of trajectories) and by bulk phospho-MK2 levels measured by Western Blotting in response to three doses of P3C4, CpG, LPS, or TNF (0.1, 1, 100 ng/ml for P3C4, LPS, TNF; 1, 10, 1000 nM for CpG). Western Blotting quantification: band intensities were background corrected, normalized to tubulin control, normalized across multiple membranes using an internal control sample, and baseline-deducted; depicting data from a single experiment (Western Blot membrane shown in Fig. EV1E). For microscopy, the mean of means of trajectories from two biological replicates is shown (in total: 923, 1171, 970, and 1055 cells included in the analysis for P3C4, CpG, LPS, and TNF, respectively; same data as in Fig. 1E). (**F**) Gating strategy for flow cytometry measurements of intracellular p-p38 levels in hMPDMs. (**G**) Example quantification of p-p38^{+} fraction of cells stimulated with indicated doses of LPS for 30 min (blue). The fluorescence signal of an unstimulated, unstained sample is used to define a cutoff between p-p38^{+} and p-p38^{-} cells (gray). Data from one experiment are displayed.

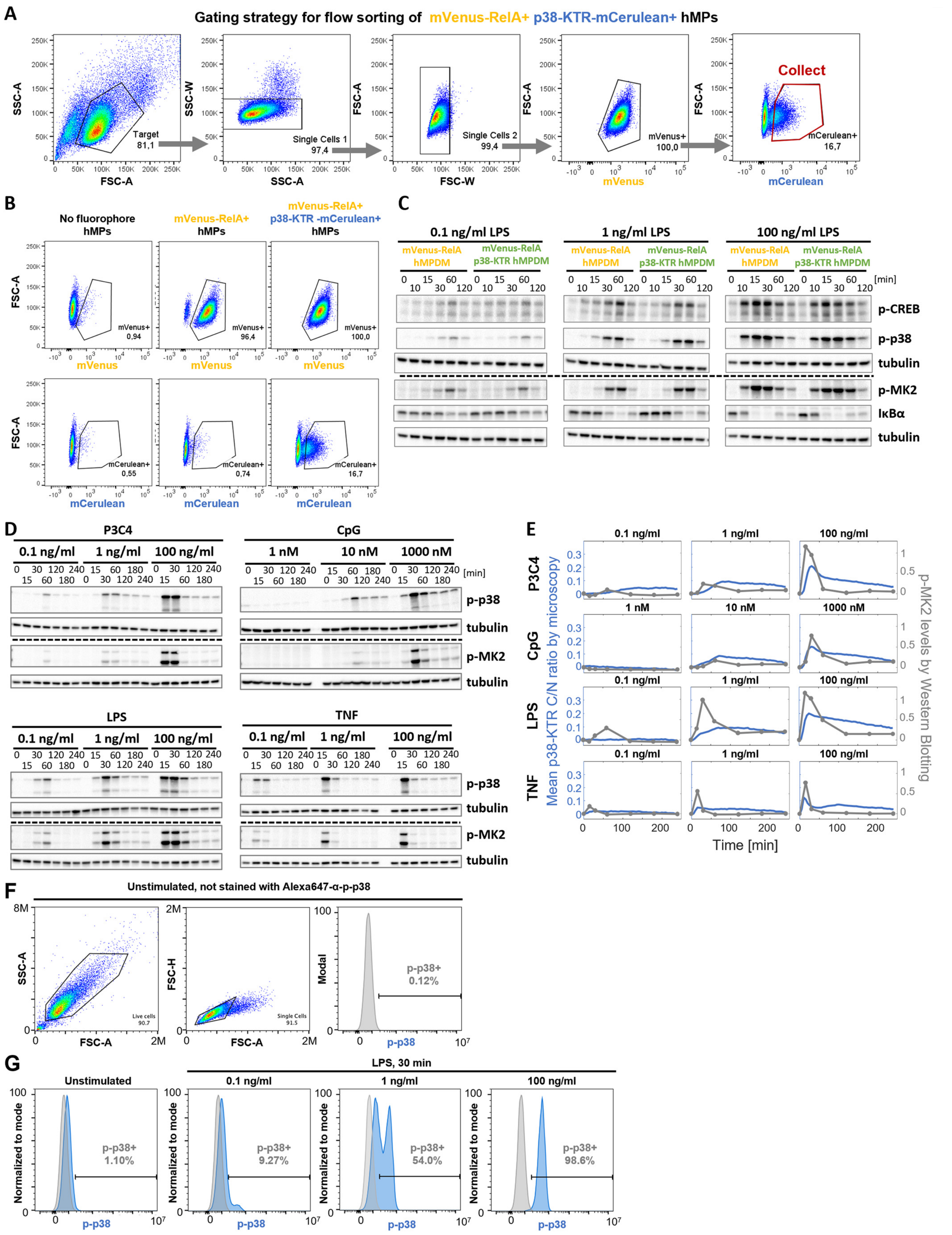
A
Gating strategy for flow sorting of mVenus-RelA+ p38-KTR-mCerulean+ hMPs
Target 81,1
Single Cells 1 97,4
Single Cells 2 99,4
mVenus+ 100,0
Collect
mCerulean+ 16,7
FSC-A
SSC-A
SSC-W
FSC-W
mVenus
mCerulean
B
No fluorophore hMPs
mVenus-RelA+ hMPs
mVenus-RelA+ p38-KTR -mCerulean+ hMPs
mVenus+ 0,94
mVenus+ 96,4
mVenus+ 100,0
mCerulean+ 0,55
mCerulean+ 0,74
mCerulean+ 16,7
C
0.1 ng/ml LPS
1 ng/ml LPS
100 ng/ml LPS
mVenus-RelA hMPDM
mVenus-RelA p38-KTR hMPDM
[min]
p-CREB
p-p38
tubulin
p-MK2
IκBα
tubulin
D
P3C4
CpG
LPS
TNF
0.1 ng/ml
1 ng/ml
100 ng/ml
1 nM
10 nM
1000 nM
[min]
p-p38
tubulin
p-MK2
tubulin
E
P3C4
CpG
LPS
TNF
Mean p38-KTR C/N ratio by microscopy
p-MK2 levels by Western Blotting
Time [min]
F
Unstimulated, not stained with Alexa647-α-p-p38
Live cells 90.7
Single Cells 91.5
p-p38+ 0.12%
SSC-A
FSC-H
Modal
FSC-A
p-p38
G
LPS, 30 min
Unstimulated
0.1 ng/ml
1 ng/ml
100 ng/ml
p-p38+ 1.10%
p-p38+ 9.27%
p-p38+ 54.0%
p-p38+ 98.6%
Normalized to mode
p-p38

 © The Author(s)

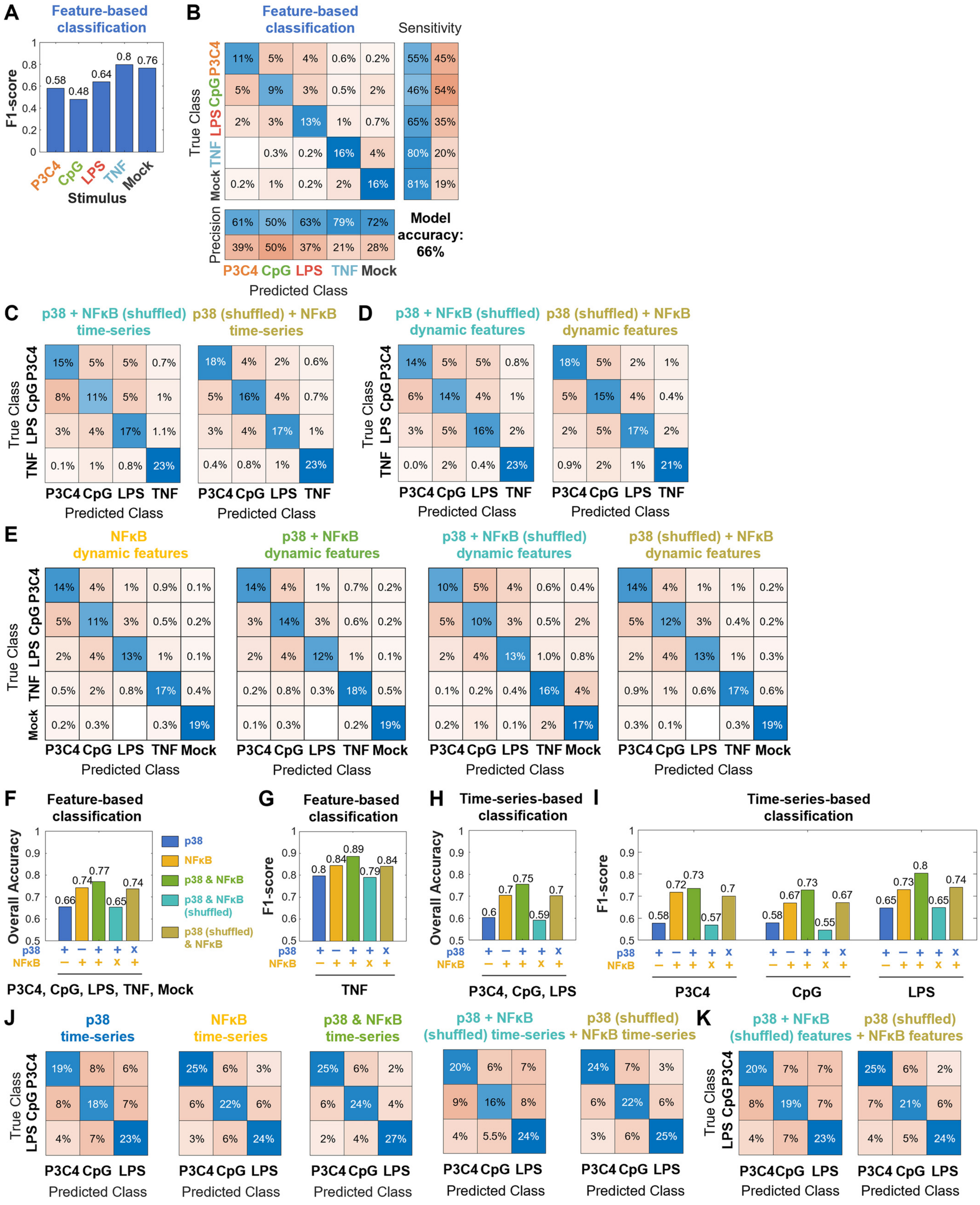
A
Feature-based classification
F1-score
0.58
0.48
0.64
0.8
0.76
P3C4 CpG LPS TNF Mock
Stimulus
B
Feature-based classification
Sensitivity
True Class
Predicted Class
Precision
Model accuracy: 66%
C
p38 + NFκB (shuffled) time-series
p38 (shuffled) + NFκB time-series
D
p38 + NFκB (shuffled) dynamic features
p38 (shuffled) + NFκB dynamic features
E
NFκB dynamic features
p38 + NFκB dynamic features
p38 + NFκB (shuffled) dynamic features
p38 (shuffled) + NFκB dynamic features
F
Feature-based classification
Overall Accuracy
p38
NFκB
p38 & NFκB
p38 & NFκB (shuffled)
p38 (shuffled) & NFκB
P3C4, CpG, LPS, TNF, Mock
G
Feature-based classification
F1-score
TNF
H
Time-series-based classification
Overall Accuracy
P3C4, CpG, LPS
I
Time-series-based classification
F1-score
P3C4
CpG
LPS
J
p38 time-series
NFκB time-series
p38 & NFκB time-series
p38 + NFκB (shuffled) time-series
p38 (shuffled) + NFκB time-series
K
p38 + NFκB (shuffled) features
p38 (shuffled) + NFκB features
True Class
Predicted Class

Figure EV2. Machine learning classifications of p38 and NFκB activities in response to four high-dose ligand stimulations or PAMP stimulations using shuffled activities as negative controls.

(**A**, **B**) F1 scores by class and confusion matrix of decision tree ensemble classification of p38 dynamic features in response to mock and high-dose P3C4, CpG, LPS, and TNF stimulations. Data from two pooled biological replicates are used. (**C**, **D**) Confusion matrices of machine learning classification of NFκB ("shuffled": incorrectly matched NFκB activities from cells randomly selected from all classes) + p38 (left) or NFκB + p38 ("shuffled": incorrectly matched p38 activities from cells randomly selected from all classes) (right) time-series (**C**) or dynamic features (**D**) in response to high-dose P3C4, CpG, LPS, and TNF stimulations. Data from two pooled biological replicates are used. (**E**) Confusion matrices of machine learning classification of decision tree ensemble classification of NFκB only, NFκB + p38, NFκB ("shuffled": incorrectly matched NFκB activities from cells randomly selected from all classes) + p38, or NFκB + p38 ("shuffled": incorrectly matched p38 activities from cells randomly selected from all classes) dynamic features in response to mock and high-dose P3C4, CpG, LPS, and TNF stimulations. Data from two pooled biological replicates are used. (**F**, **G**) Overall classification accuracy (**F**) and F1 score for TNF class (**G**) in machine learning classifications using p38 only, NFκB only, NFκB + p38, NFκB ("shuffled": incorrectly matched NFκB activities from cells randomly selected from all classes) + p38, NFκB + p38 ("shuffled": incorrectly matched p38 activities from cells randomly selected from all classes) dynamic features in response to mock and high-dose P3C4, CpG, LPS, and TNF stimulations. Data from two pooled biological replicates are used. (**H–J**) Overall classification accuracy (**H**), F1 scores for individual classes (**I**), and confusion matrices (**J**) for machine learning classifications of time series using p38 only, NFκB only, NFκB + p38, NFκB ("shuffled": incorrectly matched NFκB activities from cells randomly selected from all classes) + p38, or NFκB + p38 ("shuffled": incorrectly matched p38 activities from cells randomly selected from all classes) in response to high-dose P3C4, CpG, and LPS stimulations. Data from two pooled biological replicates are used. (**K**) Confusion matrices of machine learning classification of NFκB (shuffled among all cells in all classes) + p38 (left) or NFκB + p38 (shuffled among all cells in all classes) (right) dynamic features in response to high-dose P3C4, CpG, and LPS stimulations. Data from two pooled biological replicates are used.

 © The Author(s)

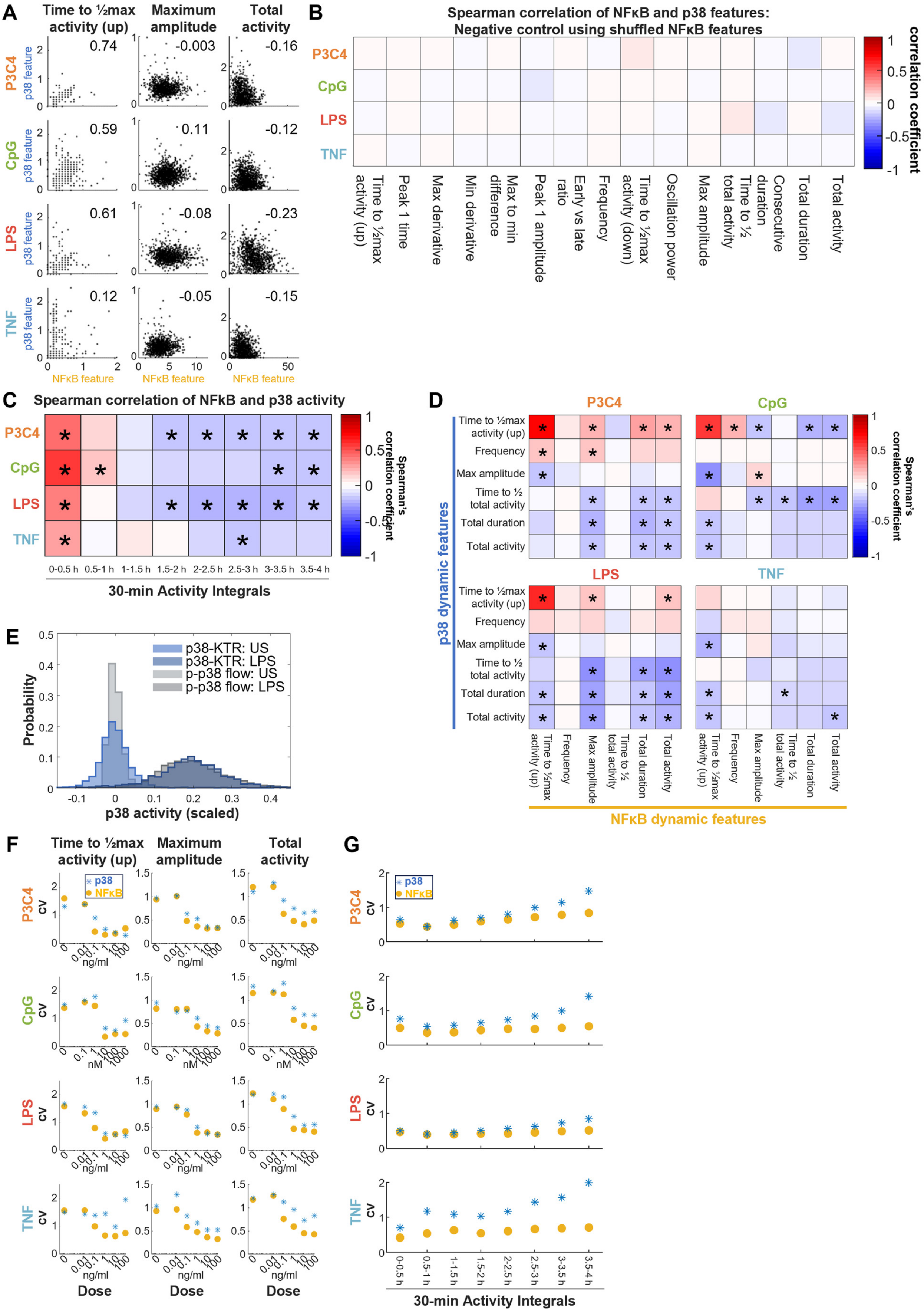
A
Time to ½max activity (up)
Maximum amplitude
Total activity
P3C4
CpG
LPS
TNF
p38 feature
NFκB feature
0.74
-0.003
-0.16
0.59
0.11
-0.12
0.61
-0.08
-0.23
0.12
-0.05
-0.15
B
Spearman correlation of NFκB and p38 features:
Negative control using shuffled NFκB features
Spearman's correlation coefficient
Time to ½max activity (up)
Peak 1 time
Max derivative
Min derivative
Max to min difference
Peak 1 amplitude
Early vs late ratio
Frequency
Time to ½max activity (down)
Oscillation power
Max amplitude
Time to ½ total activity
Consecutive duration
Total duration
Total activity
C
Spearman correlation of NFkB and p38 activity
0-0.5 h
0.5-1 h
1-1.5 h
1.5-2 h
2-2.5 h
2.5-3 h
3-3.5 h
3.5-4 h
30-min Activity Integrals
D
p38 dynamic features
Time to ½max activity (up)
Frequency
Max amplitude
Time to ½ total activity
Total duration
Total activity
NFκB dynamic features
E
p38-KTR: US
p38-KTR: LPS
p-p38 flow: US
p-p38 flow: LPS
Probability
p38 activity (scaled)
F
CV
ng/ml
nM
Dose
p38
NFκB
G
30-min Activity Integrals

Figure EV3. MAPK p38 and NFκB dynamic features are poorly correlated.

(**A**) Scatter plots of correlations of selected p38 vs. NFκB dynamic features (as in Fig. 5A) upon stimulation with high-dose P3C4, CpG, LPS, or TNF. Number indicates the Spearman correlation coefficient. Data from two pooled biological replicates are depicted. (**B**) Negative control: Spearman correlation coefficients (CCs) between indicated p38 dynamic features and corresponding NFκB dynamic feature using NFκB features randomly shuffled with respect to p38 features among cells within a stimulation condition for stimulation with high-dose P3C4, CpG, LPS, or TNF. The asterisk indicates a statistically significant correlation ($p < 0.05$) with $|CC| > 0.15$. Data from two pooled biological replicates are used. (**C**) Spearman correlation coefficients (CC) between integrals of p38 activity over 30 min from 0 to 4 h and corresponding NFκB integrals upon stimulation with high-dose P3C4, CpG, LPS, or TNF. The asterisk indicates a statistically significant correlation ($p < 0.05$) with $|CC| > 0.15$. Data from two pooled biological replicates are used. (**D**) Spearman correlation coefficients (CCs) between indicated p38 dynamic features and all indicated NFκB dynamic features upon stimulation with high-dose P3C4, CpG, LPS, or TNF. The asterisk indicates a statistically significant correlation ($p < 0.05$) with $|CC| > 0.15$. Data from two pooled biological replicates are used. (**E**) Comparison of heterogeneity of p38 activity measured by flow cytometry (gray) and p38-KTR microscopy (blue) in unstimulated cells and upon 30 min 100 ng/ml LPS stimulation. For each assay, the mean of the unstimulated sample is deducted from each distribution. The flow cytometry distributions are scaled using the distance between the peaks of lognormal fits to the unstimulated and the stimulated distributions. Microscopy: Data from two pooled biological replicates are used. Flow cytometry: Data from a single experiment are displayed. (**F**) Coefficient of variation of indicated p38 and NFκB dynamic features upon stimulation across dose range of P3C4, CpG, LPS, or TNF. Data from two pooled biological replicates are used. (**G**) Coefficient of variation of 30-min p38 and NFκB activity integrals upon stimulation with high-dose P3C4, CpG, LPS, or TNF. Data from two pooled biological replicates are used.

 © The Author(s)

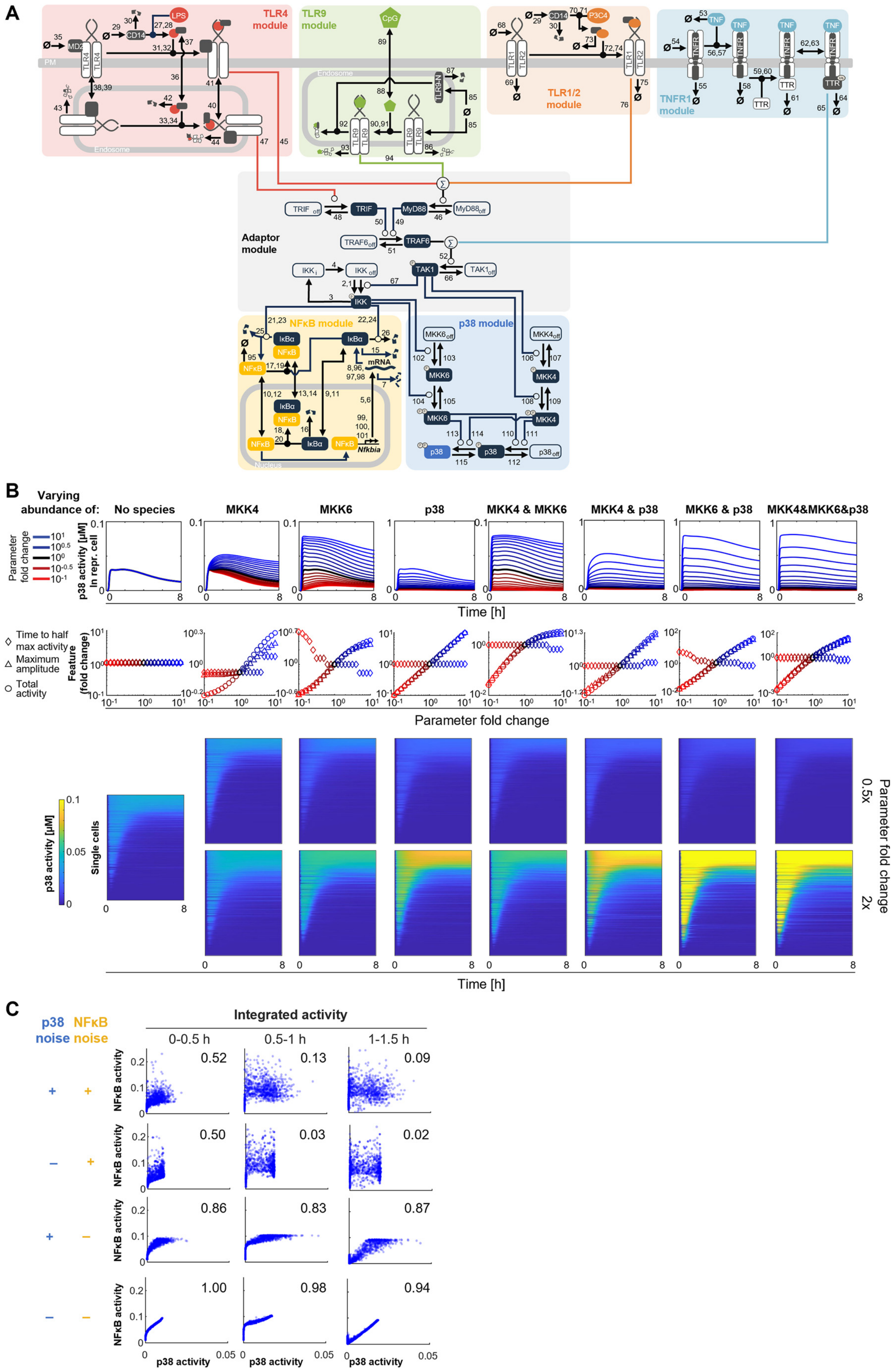
A
TLR4 module
TLR9 module
TLR1/2 module
TNFR1 module
Adaptor module
NFκB module
p38 module
B
Varying abundance of:
No species
MKK4
MKK6
p38
MKK4 & MKK6
MKK4 & p38
MKK6 & p38
MKK4&MKK6&p38
Parameter fold change
p38 activity [µM] In repr. cell
Time [h]
Time to half max activity
Maximum amplitude
Total activity
Feature (fold change)
Parameter fold change
p38 activity [µM]
Single cells
Parameter fold change
0.5x
2x
Time [h]
C
p38 noise
NFκB noise
Integrated activity
0-0.5 h
0.5-1 h
1-1.5 h
NFκB activity
p38 activity

© The Author(s)

Figure EV4. An integrated mathematical model of MAPK p38 and NFκB activation suggests sources of heterogeneity.

(**A**) Detailed schematic of mathematical model structure including reaction numbers (see also Dataset EV1). A model of p38 activation via IKK/Tpl2/MKK3/6 and TAK1/MKK4 (blue background) is integrated with established models of NFκB activation (yellow) downstream of TLR1/2 (orange), TLR9 (green), TLR4 (red), and TNF receptor (light blue) signaling (Adelaja et al, 2021; Luecke et al, 2023). (**B**) Parameter sensitivity analysis probes the effect of kinase abundances within the p38 module. Effect of variations of the abundance of indicated kinase(s) on p38 activity of a representative cell (top), p38 dynamic features in a representative cell (middle), and heterogeneous p38 activity trajectories in single cells upon simulated LPS stimulation (bottom). (**C**) Scatter plots of p38 vs. NFκB integrated activity over indicated 30 min intervals in simulated LPS stimulation with or without simulated molecular noise (i.e., using parameter distributions or fixed parameter values) in p38 and NFκB modules.

 © The Author(s)

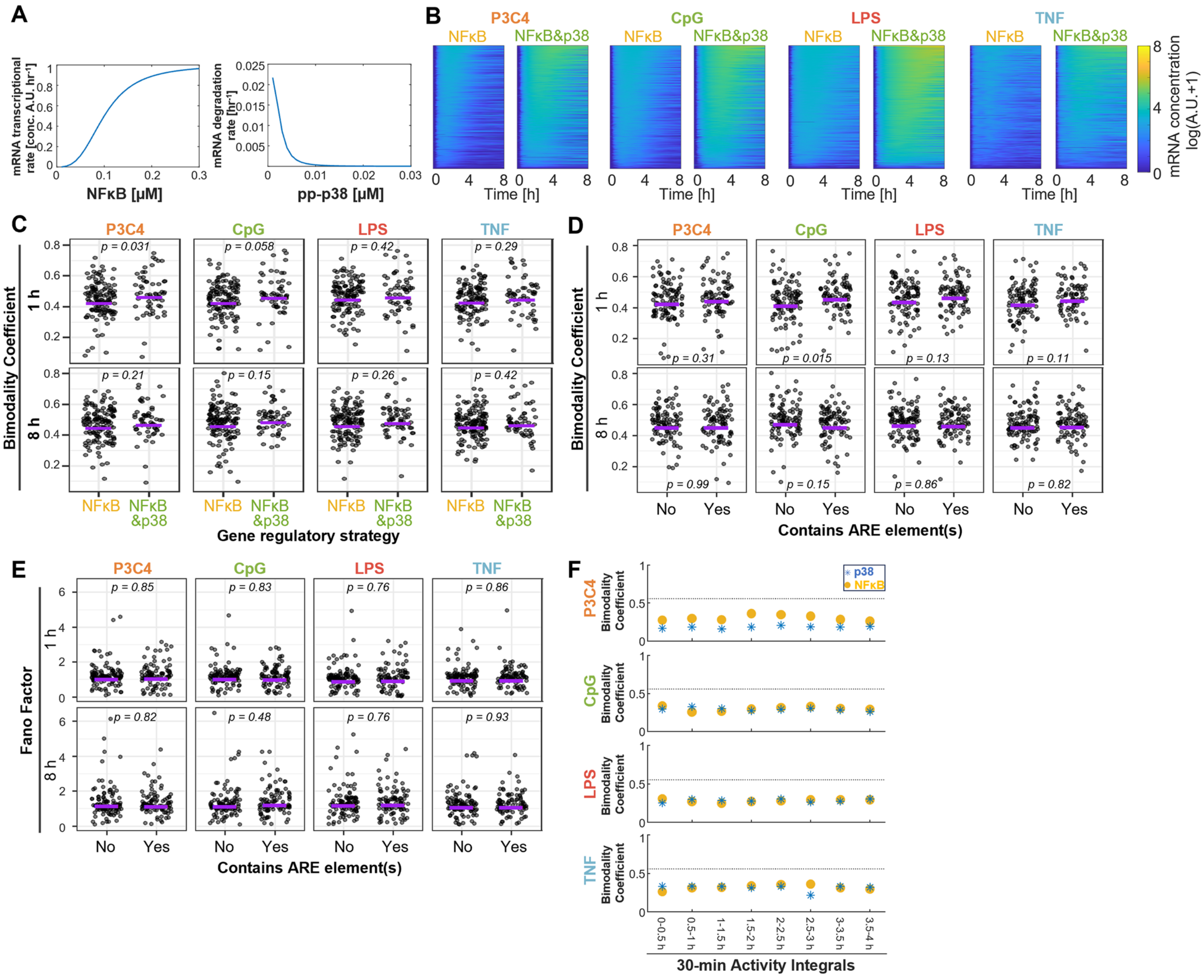

Figure EV5. MAPK p38 signaling contributes heterogeneity to macrophage gene expression responses.

(**A**) Effect of NFκB and p38 activity levels on simulated mRNA transcription and mRNA-degradation, respectively. (**B**) Simulated mRNA expression under control of NFκB only or NFκB&p38 over 8 h using experimentally determined NFκB and p38 activity upon high-dose P3C4, CpG, LPS, and TNF stimulation as input to the mathematical model. (**C**) Bimodality coefficient of all immune response genes regulated by NFκB-only (132 genes) or NFκB&p38 (59 genes) in single cells upon stimulation with P3C4, CpG, LPS, or TNF at 1 and 8 h. Purple line indicates mean. Statistical significance was determined using a permutation test for difference in means using 10000 permutations. Data from one experiment are depicted. (**D**, **E**) Bimodality coefficient (**D**) and Fano Factor (**E**) of all immune response genes in single cells categorized by absence or presence of ARE-element(s) upon stimulation with P3C4, CpG, LPS, or TNF at 1 and 8 h. Purple line indicates mean. Statistical significance was determined using a permutation test for difference in means using 10000 permutations. Data from one experiment are depicted. (**F**) Bimodality Coefficients of 30-min p38 and NFκB activity integrals upon stimulation with high-dose P3C4, CpG, LPS, or TNF determined by dual reporter macrophage imaging. Data from two pooled biological replicates are used.
